# Chemical and linguistic considerations for encoding Chinese characters: an embodiment using chain-end degradable sequence-defined oligourethanes created by consecutive solid phase click chemistry†

**DOI:** 10.1039/d3sc06189b

**Published:** 2024-03-11

**Authors:** Le Zhang, Todd B. Krause, Harnimarta Deol, Bipin Pandey, Qifan Xiao, Hyun Meen Park, Brent L. Iverson, Danny Law, Eric V. Anslyn

**Affiliations:** a Department of Chemistry, The University of Texas at Austin TX 78721 USA iversonb@austin.utexas.edu anslyn@austin.utexas.edu; b Department of Linguistics, The University of Texas at Austin TX 78721 USA dannylaw@austin.utexas.edu; c Linguistics Research Center, The University of Texas at Austin TX 78712 USA

## Abstract

Sequence-defined polymers (SDPs) are currently being investigated for use as information storage media. As the number of monomers in the SDPs increases, with a corresponding increase in mathematical base, the use of tandem-MS for *de novo* sequencing becomes more challenging. In contrast, chain-end degradation routines are truly *de novo*, potentially allowing very large mathematical bases for encoding. While alphabetic scripts have a few dozen symbols, logographic scripts, such as Chinese, can have several thousand symbols. Using a new *in situ* consecutive click reaction approach on an oligourethane backbone for writing, and a previously reported chain-end degradation routine for reading, we encoded/decoded a confucius proverb written in Chinese characters using two encoding schemes: Unicode and Zhèng Mă. Unicode is an internationally standardized arbitrary string of hexadecimal (base-16) symbols which efficiently encodes uniquely identifiable symbols but requires complete fidelity of transmission, or context-based inferential strategies to be interpreted. The Zhèng Mă approach encodes with a base-26 system using the visual characteristics and internal composition of Chinese characters themselves, which leads to greater ambiguity of encoded strings, but more robust retrievability of information from partial or corrupted encodings. The application of information-encoded oligourethanes to two different encoding systems allowed us to establish their flexibility and versatility for data storage. We found the oligourethanes immensely adaptable to both encoding schemes for Chinese characters, and we highlight the expected tradeoff between the efficiency and uniqueness of Unicode encoding on the one hand, and the fidelity to a scripts' particular visual characteristics on the other.

## Abstract 2

The information (a proverb from The Analects of Confucius) is stored in chain-end degradable sequence-defined oligourethanes, sequenced with liquid chromatography-mass spectrometry (LC-MS) under suitable conditions and decrypted with in-house Python scripts.

## Graphical abstract

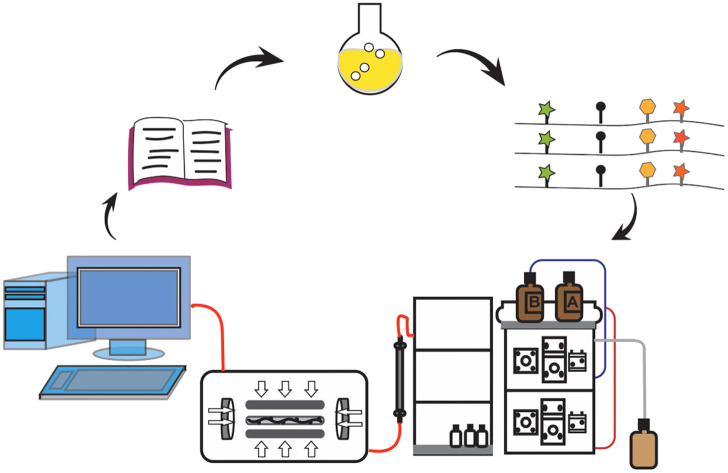

## Introduction

**Fig. 1:**
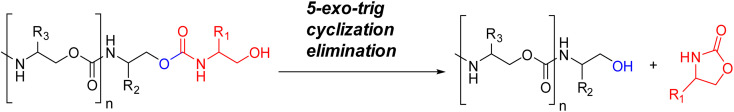
Sequencing oligourethanes with 5-*exo-trig* cyclization.

tSequenced-defined polymers (SDPs), such as polyureas, nucleic acids and peptides, have been used in applications as catalysts, foldamers, self-assembled materials, and biomaterials.^1,2^ Inspired by DNA, which stores the genetic blueprint for life on earth based upon four monomers (A, T, C, G),^3^ SDPs have also attracted attention as durable and dense information storage media.^4–6^ Lutz, Du Prez, and others^7–10^ have introduced several designs of abiotic SDPs to store information precisely and efficiently. In most cases the decoding process requires tandem Mass Spectra (MS) analysis, analogous to its use in proteomics.^11,12^ However, *de novo* sequencing using MS/MS becomes increasingly challenging as the number of monomers increases. In proteomics, one typically knows the sequences being sought, and they are identified by comparison to a database.^13,14^ In contrast, chain-end degradation sequencing routines, such as Edman degradation,^15^ are entirely *de novo*. By analogy, our lab developed a chain-end degradation routine for sequencing oligourethanes (OUs) that can be readily performed using liquid chromatography-mass spectroscopy (LC-MS).^3,16–18^ The method sequentially and incrementally eliminates monomers *via* a 5-*exo-trig* cyclization from the O-terminus (Fig. 1).^19^ Using this method, we reported the encoding of a text passage from Jane Austen's Mansfield Park with a hexadecimal symbolic-code (base-16), which could be read independently without prior knowledge of the information stored.^20^ Further, due to the simple deconvolution process, we showed that eight 10-mer OUs can be decoded simultaneously by the use of mass-tags that sort the mixtures of OUs.^21^ However, the labor involved in the synthesis of each monomer, one at a time, prior to incorporation into the polymer *via* solid-phase synthesis, limits the number of monomers that can be incorporated. Thus, the ability to expand the palette of encoding monomers to any base required for a particular encoding scheme *via* monomer synthesis during polymer synthesis would be a major advance for the field.^22–24^

The prior use of SDPs for information encoding has focused on text passages in languages written in alphabetic systems.^20,25^ Expanding the palette of encoding monomers available allows an exploration of novel strategies for encoding different writing systems. Binary encodings are natural in the context of computers' recognition of a simple on/off distinction. Alphabetic scripts typically consist of a small number of characters and little meaningful information based on visual similarities between those characters. However, many East Asian writing systems are logographic, where the symbols can represent whole words. The characters in such systems often number in the tens of thousands. Morpho-syllabic Chinese characters each represent a syllable with distinct meanings, but also contain visual elements that meaningfully relate those characters to other visually similar characters. Further, the symbols historically cue aspects of the character's meaning or pronunciation, and in some cases visually disambiguate words that have the same pronunciation (homophones). Different methods of encoding and decoding Chinese characters make different decisions about what meaningful aspects of these visual relations between characters are encoded or ignored.^27^

**Fig. 2:**
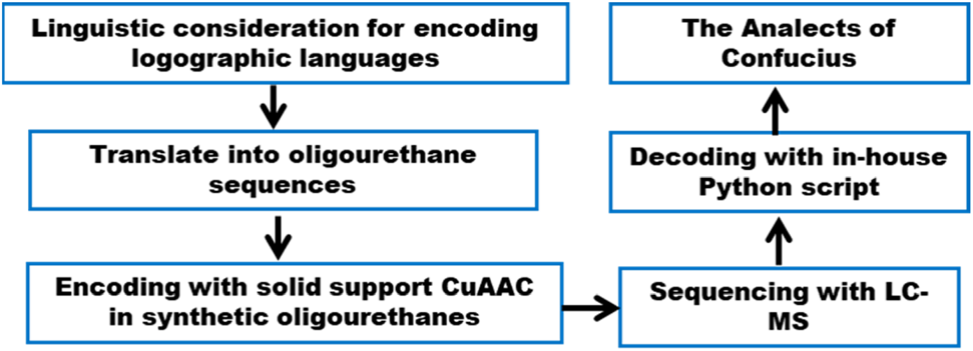
The workflow of this project.

Here, we apply our chemical methods to two existing encoding schemes that are attuned to different characteristics of logographic writing systems^26–30^ to establish the SDPs' adaptability, by encoding and decoding a confucius proverb (Fig. 2). In advance of creating SDPs for encoding Chinese characters, we reviewed several linguistic approaches and selected two representative encoding schemes: Unicode and the Zhèng Mă (ZM) method, which privilege informational efficiency and visual fidelity, respectively.

### Encoding and linguistic considerations

Currently, Unicode provides the most commonly employed character encoding scheme.^31^ This system encodes the symbols from common alphabets and syllabaries employed in the Americas, Europe, Africa, and parts of Asia, as well as the logographic symbols used traditionally, and, in some cases, currently, in several East Asian countries like China, Japan, and North and South Korea, as well as ancient world scripts such as Egyptian Hieroglyphs. Unicode posits a code space divided into myriad cells; each cell receives a unique index, a hexadecimal number known as a code point. A given cell may contain a symbol or remain (as yet) unoccupied: when occupied, the symbol in the cell is uniquely identified by the cell's code point. This schema treats alphabetic and logographic symbols equivalently – as Unicode characters. For example, the lowercase letter *z* of the Roman alphabet receives the identifier U+007A (where the prefix ‘U+’ is followed by the hexadecimal code point for a Unicode character), while the Mandarin symbol 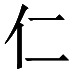 (rén, benevolence) corresponds to U+4EC1.^26^ Distinct identifiers represent distinct symbols in all instantiations of Unicode.^32^

Unicode intends to identify individual characters uniquely and efficiently across all major World scripts. Though issues surrounding an original symbol's subsequent variation in distinct milieus persist in Unicode, its adoption presents a notable expansion beyond the more limited ASCII-based encoding used in our previous work.^19^ However, Unicode does not encode visual information about characters or their internal composition, but instead arbitrarily assigns codes within a particular range. Thus, similar codes rarely imply similar characters, and *vice versa*. If even one element of the code point is lost or corrupted, an incorrectly identified character will be unrelated to the intended character. By contrast, a system based on the visual composition of characters encodes meaningful information with each element of the code string, so that mistakes in the encoding or decoding process may still yield characters similar to the intended target. While earlier work explored efficiency, *e.g.*, through Huffman encoding,^19^ the present work seeks instead to explore the range of encoding styles supported by SDPs in an effort to spur novel approaches to preserving unique characteristics among World writing systems.

To explore SDPs' range of applicability using the same monomers, we sought a character encoding scheme capturing visual characteristics and internal composition of Chinese characters. Historically several such schemes have been used. Among the earliest, the Four-Corner (FC) method,^33^ devised in the 1920s, distinguishes 10 basic stroke shapes. It encodes each character by 4 digits recording the stroke shapes in a character's four corners. However, the resulting codes are far from unique: the FC method's conflict code rate (CCR, roughly the percentage of Chinese characters whose code corresponds to more than one character) approaches 85%.^34^ Thus, we felt the FC method was not optimal for using SDPs, where the symbol's context is unknown.

**Fig. 3:**
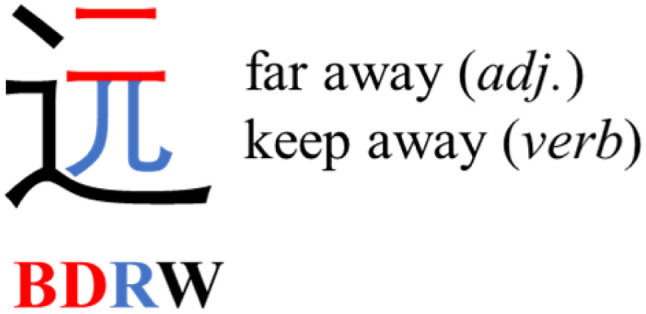
Example of encoding a Chinese character with the Zhèng Mă (ZM) method. The method decomposes the character 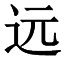 into 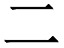 (èr, code: BD) in red, 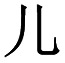 (ér, code: RD) in blue, and 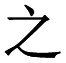 (zhī, code: W or WA) in black. Using the code W or WA for 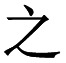 and interpreting this as the bottom (*i.e.* last) element, this yields the code BD + R(D) + W(A) = BDRW for the entire character. But writing 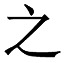 as W and interpreting this as the leftmost (*i.e.* first) element, the same components yield the code W + B(D) + RD = WBRD.

Several approaches reduce such ambiguities.^29,35^ The ZM method, from the early 1990s, reduces ambiguities^36^ to a CCR of just over 9%.^34^ In contrast to the FC method, ZM foregrounds characters' internal structure and maps Chinese characters to the standard QWERTY keyboard. ZM decomposes a character into distinct compositional elements (similar but not identical to traditional ‘radicals’), known as roots: groups of strokes that always appear as a unit, whether as a standalone character, or as a component repeated within numerous other characters. ZM divides roots into two classes, primary and secondary (Fig. 3). It maps primary roots to 1-letter strings of the QWERTY keyboard (the 26 letters of the english alphabet), and secondary roots to 2-letter strings. The method then decomposes a character into a sequence of primary and secondary roots in left-to-right, top-to-bottom order, and encodes the character by the sequence of strings corresponding to the roots. But ZM also imposes a set of rules to stipulate that no complete character code exceed 4 letters on the keyboard.^36^ With a list of the predefined correspondences between QWERTY letters and primary or secondary roots, a user can generate the 4-letter ZM code for any Chinese character. Thus, with 4 elements over a 26-symbol base, this allows 26^4^ = 456 976 potential codes, roughly 10 times the current number of Chinese characters. This makes the ZM method a visually attuned system for encoding Chinese characters which can be stored as individual 4-mer oligourethanes using a base-26 encoding capacity.

Because, unlike Unicode, ZM does not achieve total uniqueness (∼9% CCR),^34^ a single Chinese character might not correspond to a single code, and *vice versa*. Some individual characters correspond to a variety of codes simply due to ambiguity in the order for listing the roots comprising the character: *e.g.*, 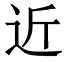 (jìn, be near) corresponds to ZM codes PDW and WPD. Considering this potential for ambiguity, one benefit of an encoding scheme motivated by the visual layout of a character is that incorrectly identified characters will likely be visually similar to the intended character. Thus, while errors are more likely using ZM than Unicode, ZM errors will plausibly involve visually similar characters, rather than an entirely unrelated (and possibly not even Chinese) character, as might be the case with a Unicode error. We selected a quote (see below) to illustrate these redundancies, and we explore different heuristics needed to incorporate ZM into a viable SDP data storage workflow using OUs as the example.

### Oligourethane considerations

Given the different strengths and challenges of Unicode and the ZM methods for encoding Chinese characters, we set out to design an oligourethane (OU) encoding (writing) and chain-end sequencing (reading) technique for Chinese characters adaptable to both methods, where individual OUs would code for a specific logographic character. First, in both Unicode and ZM schemes, each OU would require only four to five monomers to represent a single character, and hence the OUs could be quite short. Second, while we have already demonstrated the ability to write in hexadecimal,^37^ as needed for Unicode, the ZM method uses 26 symbols and therefore would require us to synthesize 26 unique monomers. Thus, we turned to exploring *in situ* on-resin synthetic methods (see below) to avoid having to create unique monomers. With this strategy, we eliminate the synthesis and purification steps for each individual monomer, and further, a consecutive on-resin monomer synthesis allows for the generation of a large library of masses that will dramatically enhance the encoding capacity in the future.^22–24^

To demonstrate our synthetic approach and its ability to encode the complexity of a logographic writing system, we chose an eight-character proverb from the Analects of confucius: 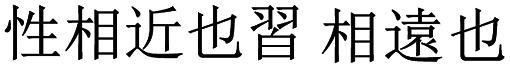,^38,39^ roughly “By nature [people] are near each other; by habitual action they become farther apart”.^40^ To further probe the versatility and adaptability of the approach, we encoded the proverb in both traditional and simplified Chinese characters (Table 1). The former appear in manuscripts through the centuries, but also find current use in Hong Kong, Taiwan, and other diaspora communities; the latter stem in part from earlier informal writing practices but were formalized over the 20th century into a system streamlined for modern writing needs in the People's Republic of China. While either system could in theory encode either script, we utilized the ZM method to encode the more recent simplified characters and Unicode for the more numerous traditional characters.

**Table 1:** Chinese symbols to be encoded, traditional and simplified, pinyin (pronunciation), and english translations

Traditional	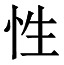	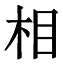	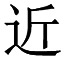	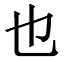	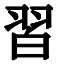	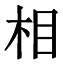	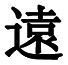	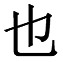
Simplified	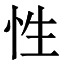	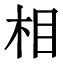	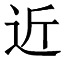	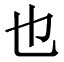	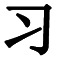	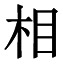	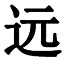	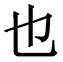
Pinyin	Xìng	Xiāng	Jìn	Yě	Xí	Xiāng	Yuăn	Yě
English	Natures	Mutually	Are close	Also	Practices	Mutually	Are far	Also

## Results and discussion

### Chemical results and advances

**Fig. 4:**
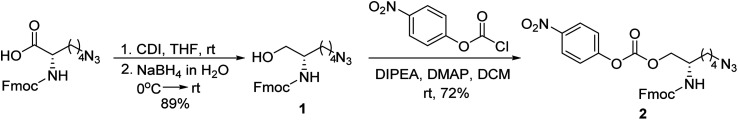
Synthetic route for monomer 2.

Our previous work using oligourethanes for encoding introduced each monomer serially using solid phase synthesis.^20^ This synthetic approach requires individual monomers, each of which is synthesized independently in an O-terminus activated and N-terminus protected form. As alluded to above, we envisioned creating different monomers concurrently with the oligomer synthesis, adding side chains of varying mass to a common monomer. In order to fulfill this vision, we screened several reactions – Diels Alder,^41^ Suzuki couplings,^42,43^ thia Michael additions,^44^ and copper catalyzed azide–alkyne click (CuAAC) chemistry^45^ – for their efficiency or reaction on resin, each of which are well-known to give high yields in solution. We found that only the copper catalyzed click was compatible with the resin we were using for the solid phase synthesis of the OUs, giving ∼95% yields, while the other reactions furnished low yields, or the resins were damaged by the reaction conditions. Therefore, we moved forward with CuAAC to achieve our goal. Our synthesis commenced with the reduction of Fmoc-l-azidolysine to furnish compound 1, followed by activation of 1 with 4-nitrophenyl chloroformate, to furnish the monomer 2 in good yield (Fig. 4). We utilized l-phenylalaninol loaded 2-chlorotrityl polystyrene resin and l-alaninol loaded 2-chlorotrityl polystyrene resin as the solid support to start the synthesis, and hence one of either of these two monomers is consistently on the O-terminus of the resulting oligourethanes.

**Fig. 5:**
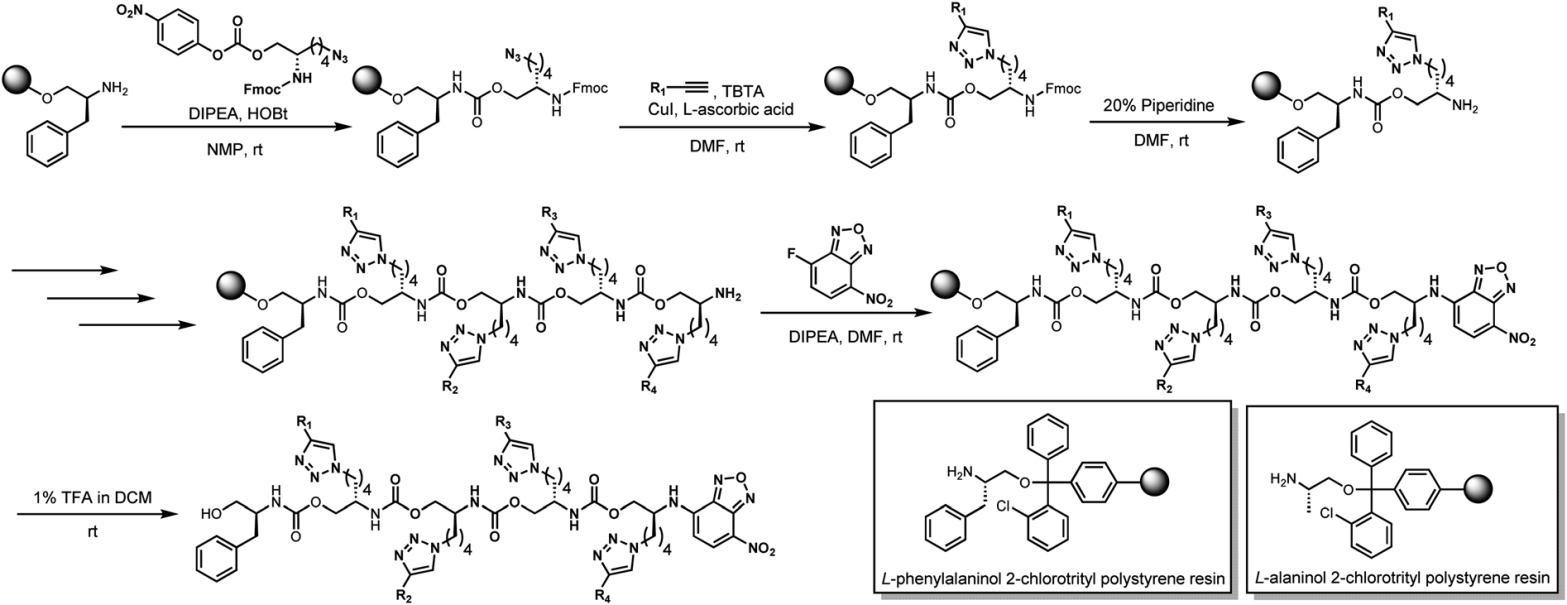
Synthesis of novel oligourethanes *via* consecutive solid phase click chemistry.

Starting with our published conditions for oligourethane synthesis,^19^ monomer 2 was first appended to the resins. However, instead of deprotecting the Fmoc to then add another monomer, we exposed the resin (1 eq.) to 0.25 equivalent CuI, 0.5 equivalent sodium ascorbate and 0.5 equivalent tri(benzyltriazolylmethyl)amine (TBTA) for CuAAC click, along with 5 equivalents of the specific alkyne desired for encoding (see below). Then, following Fmoc deprotection, a second monomer 2 was coupled, and so on (Fig. 5), until completing the synthesis of the entire oligomer. In this manner, we could achieve an oligourethane capable of carrying as many different R groups as necessary for the mathematical base we are writing in (base-16 for Unicode, base-26 for ZM). Considering the number of mass differentiated terminal alkynes that exist in the chemistry world, the mathematical base can be substantially increased, which is a significant advance for the field of digital polymers and information encoding, because larger bases allow for denser information storage.

In our very first test of the CuAAC click reaction on resin, an acceptable yield of 94% was achieved. However, our biggest concern was that accumulation of CuI and ascorbic acid over multiple cycles of organic solvents and reagents would damage the resin, possibly *via* Fenton-type chemistry. Because we needed to run several consecutive click reactions to have multiple different R groups on the oligourethane string, we anticipated that several exposures to CuI and ascorbic acid in conjunction with repeated swelling and shrinking of the resin throughout the steps could lead to loss of function. However, based on our results, the resins are robust enough to tolerate the repeated exposures, highlighting the power and utility of the CuAAC click chemistry.^46^ At the end of the synthesis, as we have previously published, the chromophore NBD was appended for analysis by LC-MS.

With the reaction condition described above, we successfully synthesized 12 urethane-based oligomers (2 dimers, 2 trimers and 8 tetramers). The initial synthesis step (both coupling and click reaction) consistently proceeds smoothly. We attribute the high conversion to the absence of inorganic salt accumulation and the ready accessibility of the short chain on the resin. Generally, the conversions decrease as the number of steps increases while some truncated oligomers are observed. The conversions of the 12 oligomers ranged from 38% to 90%. Out of the 12 synthesized oligomers, 9 yielded more than 60% conversion, while only 3 urethane oligomers resulted in less than 50% conversion, which, unsurprisingly, were all tetramers. However, one of the tetramers (oligomer 2) yielded an 81% conversion, which is notably close to the conversions observed for dimers and trimers. This illustrates that the reactivity of different click reaction partners (alkynes) influences the conversions, in addition to the number of steps. As this is a consecutive reaction without stepwise purification needed in the process, the stepwise conversions are not calculated. It's worth noting that only small amounts of materials (<1 mg) are required for sequencing step after the target oligomers are made. Hence, we do not collect the entire sample from the HPLC, nor do we calculate a yield because the resin loading is often variable and imprecise, just as with solid-phase peptide synthesis where yields are routinely not reported.

**Fig. 6:**
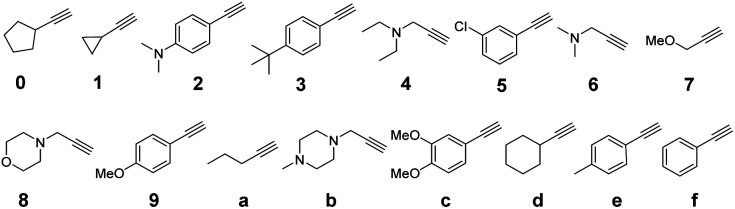
Library of 16 terminal alkynes for click chemistry for Unicode.

We first used Unicode for traditional Chinese characters. Molecular-level encoding in hexadecimal required that each symbol be represented by appending a single alkyne, of sixteen, as a coupling partner on the azido side chain of a monomer along the oligomer backbone. Therefore, a library of sixteen different commercially available mass-separated terminal alkynes was identified (Fig. 6). Two chemical principles were used to guide library design. First, the masses of all the terminal alkynes differed by at least 2 atomic mass units to enable robust differentiation by LC-MS. Second, no reactive nucleophilic functional groups were present, thereby avoiding side-reactions during urethane coupling. During the building of this library, it was quite easy to identify 32, 64, and 128 commercially available alkynes that fit our criteria, which speaks to the future possibilities for highly dense information storage using this approach to writing.

Table 2 shows the hexadecimal Unicode code points for the traditional Chinese characters of the proverb discussed above. The individual hexadecimal symbols were assigned in 1-to-1 fashion to sixteen different alkynes (Fig. 6). After assigning monomer to code points, we successfully synthesized the required eight oligomers (see ESI III(d)(1)†) *via* a combination of consecutive solid phase CuAAC clicks and urethane coupling reactions, followed by prep-HPLC for purification. The O-terminus of each OU starts with the resin preloaded alaninol (labeled with # in the sequence) or phenylalaninol (labeled with * in the sequence), which we have reported acts as a convenient indexing tool (Ala_index_ or Phe_index_) to start reading of the mass spectra.^19^

**Table 2:** Unicode code points assigned for traditional Chinese characters in the proverb

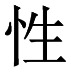	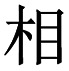	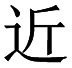	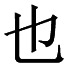	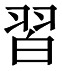	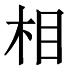	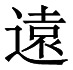	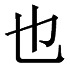
6027	76f8	8fd1	4e5f	7fd2	76f8	9060	4e5f

**Fig. 7:**
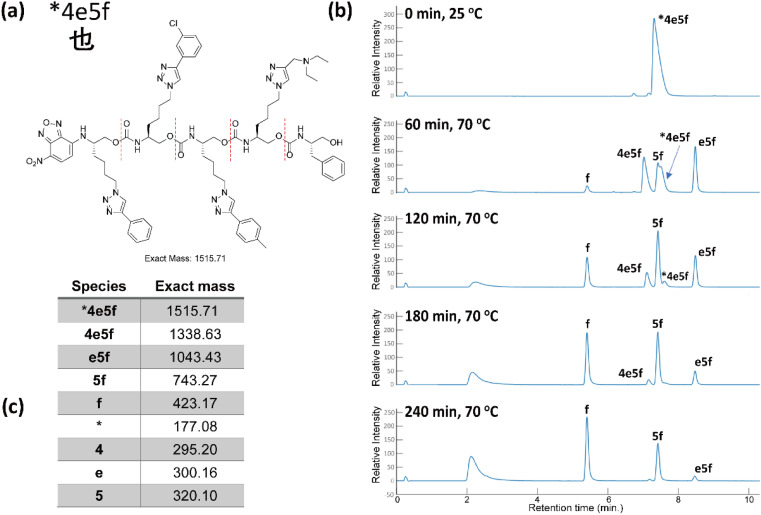
(a) The Unicode code point for the corresponding Chinese character, and the associated oligourethane. (b) The LC trace of sequencing oligourethanes with K_3_PO_4_, reaction was heated at 70 °C in a microwave. (c) The corresponding exact masses of the oligomer each truncated oligomer (see corresponding mass spectra in ESI†).

The eight oligomers were sequenced in a 2 : 1 MeOH/H_2_O mixture with K_3_PO_4_ at 70 °C and submitted to LC-MS analysis at specific intervals for a period of 4 h. As a representative example, Fig. 7 shows that chain end degradation removes each monomer from the O-terminus, thus truncating the oligomers iteratively. 27 out of 32 masses were observed clearly and distinctly. The precursor 4 mers #8fd1, #7fd2, #9060 overlapped with one of their truncated oligomers in the low-resolution LC-MS conditions due to their similar polarity. It is worth noting that the length of the truncated oligomers does not correlate with the polarity, resulting in disordered retention times for each moiety from an LC trace. However, one can easily identify which LC peaks grew and diminished in sequence over time. Using mass spectrometry, we could easily observe +1 and +2 charged moieties, facilitating identification of all the moieties by intensity difference of oligomers/truncated oligomers and the mass differences (see ESI III(c)†).

Having thus decoded the stored Unicode code points, we notate the hexadecimal codes in a Python list. We then feed this to a short Python function in a Jupyter notebook developed in house which prints the characters corresponding to the Unicode code points, reconstructing the original text with no errors nor any biased foreknowledge of the proverb, as in our previous work.^19,20^

**Fig. 8:**
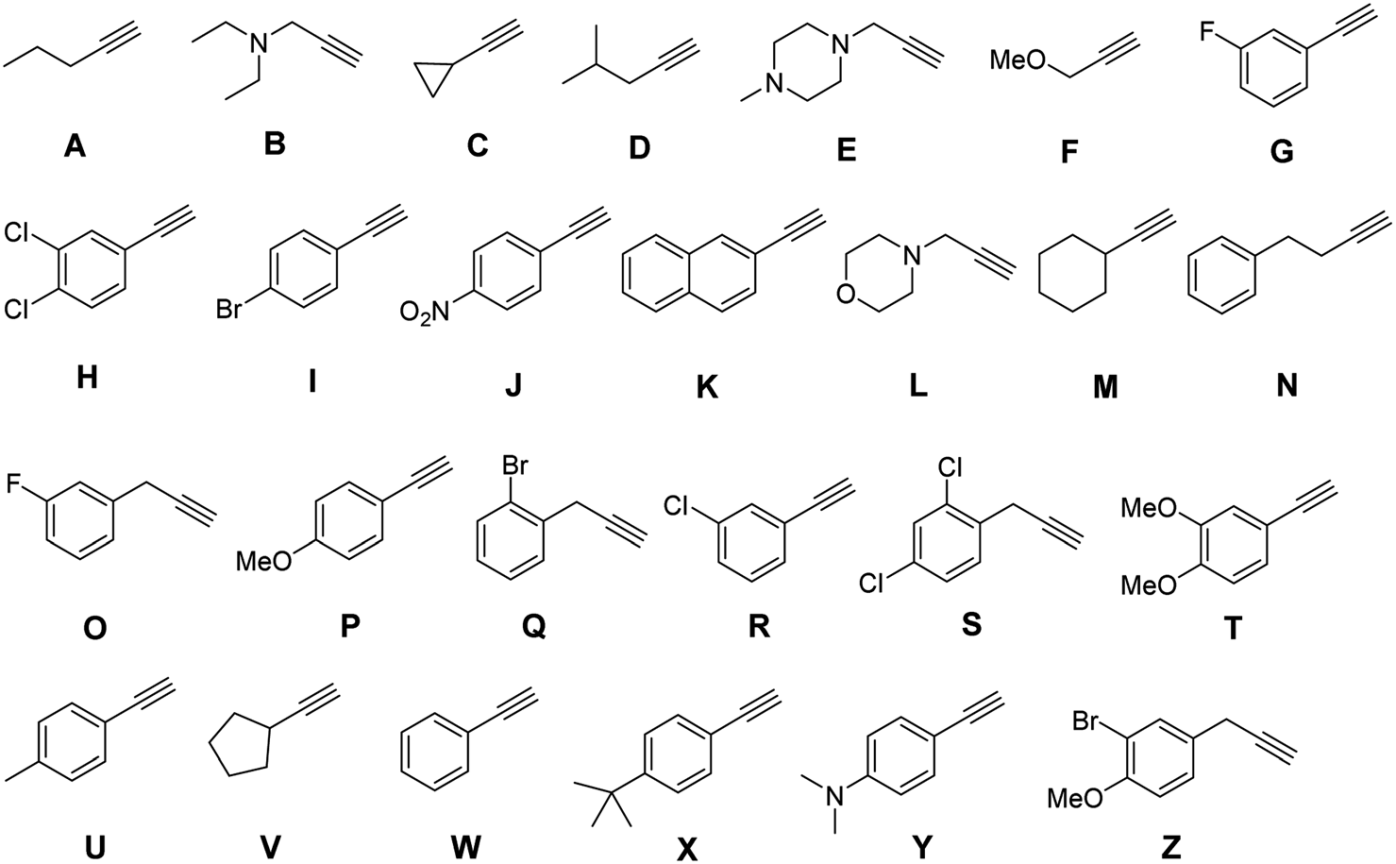
Library of 26 terminal alkynes for click chemistry for the Zhèng Mă encoding.

With the success of our encoding of traditional Chinese characters with Unicode, we moved to encoding the same proverb in simplified Chinese characters with the ZM method.^47^ Thus, the proverb was converted to a base-26 symbolic system simply by increasing our library to 26 terminal alkynes (Fig. 8). In addition, ZM only requires four letters as a maximum code length but permits shorter codes. This provides opportunities for employing single and short string oligourethanes (*e.g.*, 2-mers, 3-mers, or 4-mers) to encode a single character. When including the indexing monomer, this led to three 2-mers, two 3-mers and three 4-mers (Table 3), corresponding to the eight simplified Chinese characters (see ESI III(d)(2)†). The synthesis of the OUs was performed as for Unicode encoding, by iterative couplings, deprotections, solid phase CuAAC clicks, and capping with NBD. Cleavage from the resin was performed with 1% trifluoroacetic acid (TFA) in dichloromethane (DCM) for 10 min. Purification with HPLC was performed before sequencing.

**Table 3:** Zhèng Mă code assigned for simplified Chinese characters in the proverb

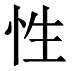	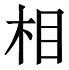	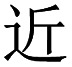	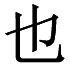	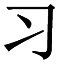	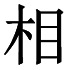	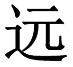	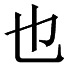
UMC	FLVV	PDW	YI	YT	FLVV	BDRW	YI

**Fig. 9:**
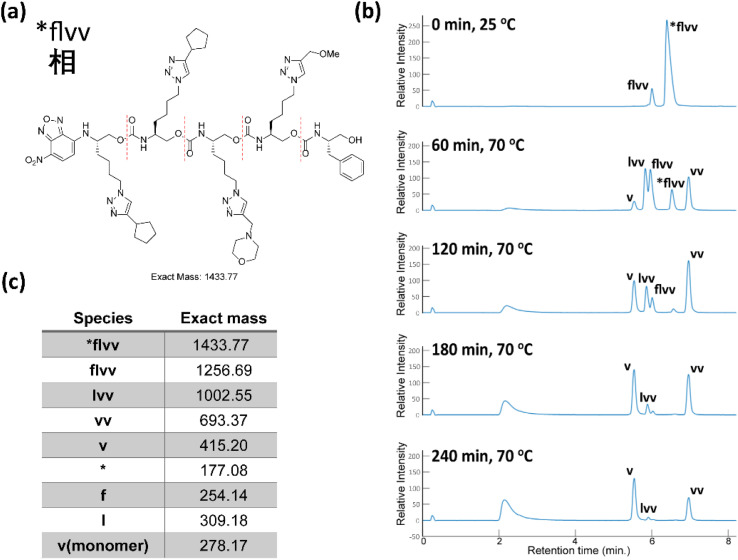
(a) The ZM code and corresponding Chinese character, and the associated oligourethane. (b) The LC trace of sequencing oligourethanes with K_3_PO_4_, reaction was heated at 70 °C in a microwave. (c) The corresponding exact masses of the oligomer and each truncated oligomer (see corresponding mass spectra in ESI†).

As with the Unicode oligomers, we sequenced these oligomers concurrently *via* chain-end degradation in a 2 : 1 MeOH/H_2_O mixture with K_3_PO_4_ at 70 °C in a heated shaker. These reactions were monitored by LC-MS every 60 min for 4 h. 23 of 24 masses (three 2-mers, two 3-mers and three 4-mers) were observed clearly and distinctly in the 470 nm channel under the generalized low-resolution LC-MS conditions (Fig. 9). The precursor 4-mer #bdrw overlapped with one of its truncated oligomers. As we discussed above, a lack of resolution between the precursor and a truncated oligomer does not cause any issues. We ran the decoding process with the in-house software to uncover the information stored within the oligourethanes. Specifically, the resulting ZM codes were passed to a Python list and fed to a specific function in the Jupyter notebook to render the appropriate Chinese characters, with additional heuristics described below to deal with ambiguous or non-unique ZM codes. Once again, the workflow returned the proper Chinese text with no errors with no foreknowledge of the proverb.

### Associated software for the linguistic considerations

As mentioned above, to assist the encoding and decoding phases of the chemical procedures, we created simple routines (*i.e.*, functions) in Python, available not only *via* a command-line script, but also *via* a Jupyter notebook to facilitate portability and transparency (see the Zhengmadification (https://github.com/LingResCtr/zhengmadification) repository on GitHub: https://github.com/LingResCtr/zhengmadification). The system imports the RIME correspondences between Chinese characters and their ZM codes to create a database in memory. A function then reads in a text string containing the desired phrase, isolates the individual characters, and converts each of these to the corresponding ZM code in the database. These codes are then assigned the monomers and their sequence in a corresponding OUs over a 26-character base, thus storing the text chemically. Decoding follows a similar procedure. Readout from OU chain-end degradation produces a collection of alphabetic codes up to 4 letters long, and another Python routine takes these codes as inputs and outputs the corresponding Chinese characters from the ZM database. The accompanying programs also include similar routines for encoding and decoding Unicode, though these are vastly simpler because Python works natively with Unicode and already includes many helper functions to support such encoding and decoding.

A principal motivation of our foray into the ZM encoding was to open the door to applying visually based encoding systems to information storage in a chemical modality, irrespective of the use of oligourethanes. In this regard, ZM's occasional lack of uniqueness provides a novel challenge to chemical encoding. To overcome the obstacles posed and create a straightforward map from text to chemical storage and back, we explored the use of heuristics. Where a single code does not uniquely specify a single Chinese character, the redundancy can derive from the encoding of multiple-character phrases. We therefore only chose single-character correspondences, omitting multiple-character strings. And when a single character corresponds to more than one code, this often derives from “shortcuts”: *i.e.*, additional shorter codes to represent a character. We therefore restricted consideration to the longest code available for any given character: *e.g.*, BRW, WBR, and BDRW can all represent 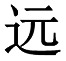 (yuăn, be far), and so we choose the longest, BDRW. This remained practical because the oligourethane synthesis routine is so simple. Nevertheless, ZM also retains same-length ambiguities such as PDW and WPD for 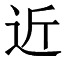 (jìn, be near), and BDRW and WBRD for 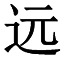 (yuăn, be far). Resolution of such cases required an additional heuristic, applying alphabetical order and choosing the first code: thus, we chose BDRW over WBRD for 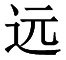 (yuăn, be far).

With these heuristics, we succeeded in closing the encoding loop: text is input and converted uniquely to ZM codes, which are then converted to unique OUs. Conversely, upon chain-end degradation a sequence of ZM codes arises, these codes are then converted to unique Chinese characters to reproduce the original text (harken back to Fig. 2). While our heuristics allowed correct identification of all characters, the lack of uniqueness introduces the possibility of incorrect identification of characters in the decoding process. But a distinct advantage of a visually based encoding scheme like ZM is that such errors will often visually approximate the target character: considering the code BDRW for 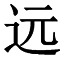 (yuăn, be far), if we had misread the final W as D, we would have obtained BDRD for 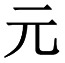 (yuán, first); or misreading the final W as G gives BDRG for 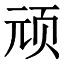 (wán, obstinate), containing the same central element 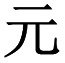 present in 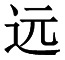. Thus, if the wrong character is selected, that character may share visual similarities with the target character, helping a competent reader to infer the correct intended character (though, as Table 3 shows with codes YI and YT, respectively 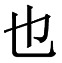 and 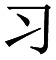, this similarity has limits). Finally, we note that the Python scripts automated the process of sifting through correspondence tables, matching Chinese characters with the corresponding Unicode or ZM codes; this step could be performed manually, avoiding the computer's binary altogether. Only the Unicode and ZM encodings are inherent to the procedure.

## Conclusions

Sequence-defined oligourethanes (OUs) were specifically designed to encode Chinese characters. Exploring the affordances of OUs for the encoding/decoding of Chinese characters required a collaborative effort between chemists and linguists. This led us to explore two different encoding schemas, allowing us to establish the flexibility and versatility of our group's use of OUs (or SDPs generally) for data storage using current industry-standard character encodings (Unicode), but also to explore their potential to store character encodings that preserve visual details of the data encoded in novel, and more application-specific, ways (ZM). Visually motivated encoding schemes like ZM fall short of Unicode in terms of uniqueness, but because every element of a ZM code is motivated by the visual character being encoded, each element provides information about the character's shape, potentially allowing greater flexibility for information retrieval in situations of corrupted or incomplete encoding. The ZM method required an expansion to base-26, and therefore we developed an *in situ* synthetic method that generates the monomers on-the-fly during the oligomer synthesis, and which could readily be expanded to much larger mathematical bases in the future. The information-encoded oligourethanes were generated, sequenced by liquid chromatography mass spectroscopy (LC-MS), and deciphered using our in-house developed software, coupled with various heuristics to sort out ZM coding redundancies. The workflow of software to design the OU sequences for the Unicode and ZM methods, chemical synthesis and sequencing, and software deciphering of the MS data with blind foreknowledge of encoded message, returned the proverb with no errors for each encoding method. Thus, we found the oligourethanes immensely adaptable to both encoding schemes.

## Data availability

Detailed experimental procedures, sequencing experiments, supplementary data, and spectral data for all new compounds. Detailed instructions for the interpretation and user manual of Python scripts are available in ESI.†

## Author contributions

LZ, TBK, QX, BLI, DL and EVA designed research, LZ, TBK, HD, BP, QX and HMP performed research, LZ, TBK, HD, BP, QX, HMP, DL and EVA analyzed data, LZ, TBK, QX, BLI, DL and EVA wrote the paper.

## Conflicts of interest

There are no conflicts to declare.

## Supplementary Material

SC-015-D3SC06189B-s001
