# Supplementary material for: Chemical and linguistic considerations for encoding Chinese characters: an embodiment using chain-end degradable sequence-defined oligourethanes created by consecutive solid phase click chemistry
Source: Chem Sci. 2024 Mar 11;15(14):5284–93. doi: 10.1039/d3sc06189b (PMC10988576; doi:10.1039/d3sc06189b)
Supplement: SC-015-D3SC06189B-s001 [file SC-015-D3SC06189B-s001.pdf]

Electronic Supplementary Material (ESI) for Chemical Science.
This journal is © The Royal Society of Chemistry 2024

Chemical and Linguistic Considerations for Encoding Chinese Characters: An Embodiment Using Chain-End Degradable Sequence-Defined Oligourethanes Created by Consecutive Solid Phase Click Chemistry

Le Zhang,[a] Todd B. Krause,[c] Harnimarta Deol,[a] Bipin Pandey,[a] Qifan Xiao,[a] Hyun Meen Park,[a] Brent L. Iverson,[a,]* Danny Law,[b,c,]* and Eric V. Anslyn[a,]*

a Department of Chemistry, The University of Texas at Austin, TX 78712, United States

b Department of Linguistics, The University of Texas at Austin, TX 78712, United States

c Linguistics Research Center, The University of Texas at Austin, TX 78712, Unites States

Emails: iversonb@austin.utexas.edu, dannylaw@austin.utexas.edu, anslyn@austin.utexas.edu

TABLE OF CONTENTS

I. General procedure and equipment 1

II. Synthesis and characterization 2

III. Sequencing experiments 8

IV. User manual for Python scripts 77

I. General procedure and equipment

All materials used in the synthesis of each compound and related tests, were purchased from Sigma-Aldrich Chemical Co., Acros Organics, Tokyo Chemical Industry, Chem Impex International, etc. and used without further purification. Solvents (DCM, NMP, chloroform, DMSO, DMF, MeOH, MeCN, Isopropanol) were of reagent grade or HPLC grade quality and purchased from Fischer Scientific. NMR solvents ($CDCl_3$-*d*, MeOD-d_4) were purchased from Cambridge Isotope Laboratories.

Column chromatography was performed using silica gel 60 (230 ± 400 mesh, 0.040 ± 0.063 mm) from Dynamic Adsorbents.

TLC analyses were carried out using Silica TLC Plates Glass Backing 20 by 20 cm sheet UV active at 254 nm.

Reverse phase column chromatography and **HPLC** purifications were performed on Shimadzu Prominence HPLC system equipped with Zorbax SB-C18 preparatory column (21.2 x 250 mm) with 7.0 µm packing material. Analytical HPLC traces were also carried out using a Zorbax SB-C18 analytical column (4.6 x 250 mm) with 5.0 µm packing material. 5-95% gradient elution (MeCN/H_2O with 0.1% formic acid). Hydrophobic urethanes utilized 30-95% gradient elution (MeCN/H_2O with 0.1% formic acid).

^{1}H and **^{13}C** spectra were recorded on Bruker AVIII with a BBFO Prodigy liquid nitrogen CryoProbe 600 MHz NMR spectrometers. The NMR spectra were referenced to solvent and the spectroscopic solvents were purchased from Cambridge Isotope Laboratories.

Liquid Chromatography/**Mass spectra** were recorded on an Agilent Technologies 6120 Single Quadrupole or 6125B Single Quadrupole mass spectrometer interfaced with an Agilent 1200 series liquid chromatography system equipped with a diode-array detector. Column: Agilent ZORBAX Eclipse Plus S2 C18 narrow bore column; 2.1 mm internal diameter; 50 mm length; 5 micron particle size; P.N. 959746-902. Resulting spectra were analysed using Agilent LC/MSD ChemStation. Separations were achieved

with a gradient elution from 5 to 95% organic, using MeCN and Water w/ 50 mM ammonium acetate as the eluents. **High resolution mass spectrometry** was performed by the UT-Austin Mass Spectrometry Facility using an Agilent Technologies 6530 Accurate-Mass Q-TOF (G6530A) with an Agilent Technologies Jet Stream ESI source, interfaced with an Agilent Technologies 1260 Infinity liquid chromatography system (G1312B). An Agilent Technologies 6546 Accurate-Mass Q-TOF (G6546A) with an Agilent Technologies Dual Jet Stream ESI source, interfaced with an Agilent Technologies 1260 Infinity II liquid chromatography system (G7112B), was used.

II. Synthesis and characterization

II(a). Synthesis and characterization of the monomer

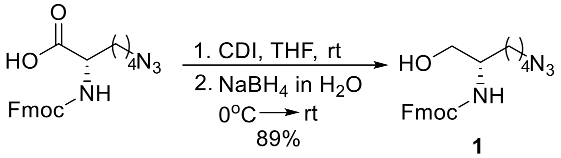

1

To a stirred solution of Fmoc-*L*-azidolysine (1.972 g, 5 mmol) in anhydrous THF (19 mL) was added N,N-carbonyldiimidazole (1.08 g, 6.7 mmol) at room temperature. The reaction stirred for at least 10 minutes and was then cooled to 0 °C. Next, a solution of $NaBH_4$ (311.7 mg, 8.24 mmol) in H_2O (8.32 mL) was added. The solution was stirred for at least 30 minutes, up to 1.5 hours. The reaction was quenched by addition of 1M HCl and extracted with EtOAc (3 x 65 mL). The combined organics were washed 1x with brine, dried over Na_2SO_4, and concentrated under vacuum. The crude product was purified by silica gel chromatography (1:1 Hexanes:EtOAc) to furnish a white solid (1.69 g, 89%). ^{1}H NMR (600 MHz, MeOD-d_4) δ 7.79 (d, *J* = 12 Hz, 2H), 7.66 (d, *J* = 12 Hz, 2H), 7.38 (t, *J* = 12 Hz, 2H), 7.30 (t, *J* = 12 Hz, 2H), 4.40-4.43 (m, 1H), 4.30-4.34 (m, 1H), 4.21 (t, *J* = 6 Hz, 1H), 3.56-3.59 (m, 1H), 3.43-3.50 (m, 2H), 3.27 (t, *J* = 6 Hz, 2H), 1.55-1.64 (m, 3H), 1.35-1.49 (m, 3H). ^{13}C NMR (125 MHz, MeOD-d_4) δ 159.13, 145.70, 145.62, 142.93, 129.05, 128.42, 126.52, 126.47, 121.21, 67.82, 65.70, 54.45, 52.65, 49.83, 32.02, 30.03, 24.56. HRMS +ESI: calculated ($C_{21}H_{24}N_4O_3Na^+$) 403.1746, found 403.1741. [M^+Na].

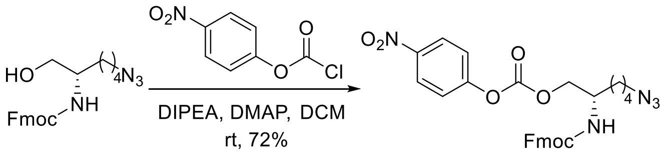

1 **2**

To a stirring solution of **1** (1.69 g, 4.45 mmol) in anhydrous DCM (22 mL) was added pyridine (378 µL, 4.67 mmol) dropwise. Next, 4-nitrophenyl chloroformate (1.08 g, 6.67 mmol) was added, and the reaction left to stir overnight. Reaction was monitored by TLC (2:1 Hexanes:EtOAc) and upon consumption of the starting material, was diluted excessively in DCM and transferred to a separatory funnel. The organic layer was washed with 1M $NaHSO_4$ (2x), then 1M Na_2CO_3 (3x), and finally brine. The organic layer was dried over Na_2SO_4, filtered, and concentrated in vacuo. The product was purified by silica gel chromatography (2:1 Hexanes:EtOAc) and isolated as a white solid (1.75 g, 3.20 mmol). ^{1}H NMR (600 MHz, $CDCl_3$-*d*) δ 8.25 (d, *J* = 12 Hz, 2H), 7.78 (d, *J* = 6 Hz, 2H), 7.59 (d, *J* = 12 Hz, 2H), 7.41 (t, *J* = 12 Hz, 2H), 7.36 (d, *J* = 12 Hz, 2H), 7.31 (t, *J* = 12 Hz, 2H), 4.82 (d, *J* = 6 Hz, 1H), 4.43-4.50 (m, 2H), 4.31-4.34 (m, 1H), 4.21-4.26 (m, 2H), 3.99-4.05 (m, 1H), 3.30 (t, *J* = 6 Hz, 2H), 1.44-1.67 (m, 6H). ^{13}C NMR (125 MHz, $CDCl_3$-*d*) δ 156.17, 155.50, 152.62, 145.60, 143.92, 143.88, 141.50, 127.93, 127.22, 125.50, 125.11, 125.08, 121.89, 120.20, 70.78, 66.89, 51.26, 50.02, 47.40, 31.05, 28.64, 23.19. HRMS +ESI: calculated ($C_{28}H_{27}N_5O_7Na^+$) 568.1808, found 568.1799. [M^+Na].

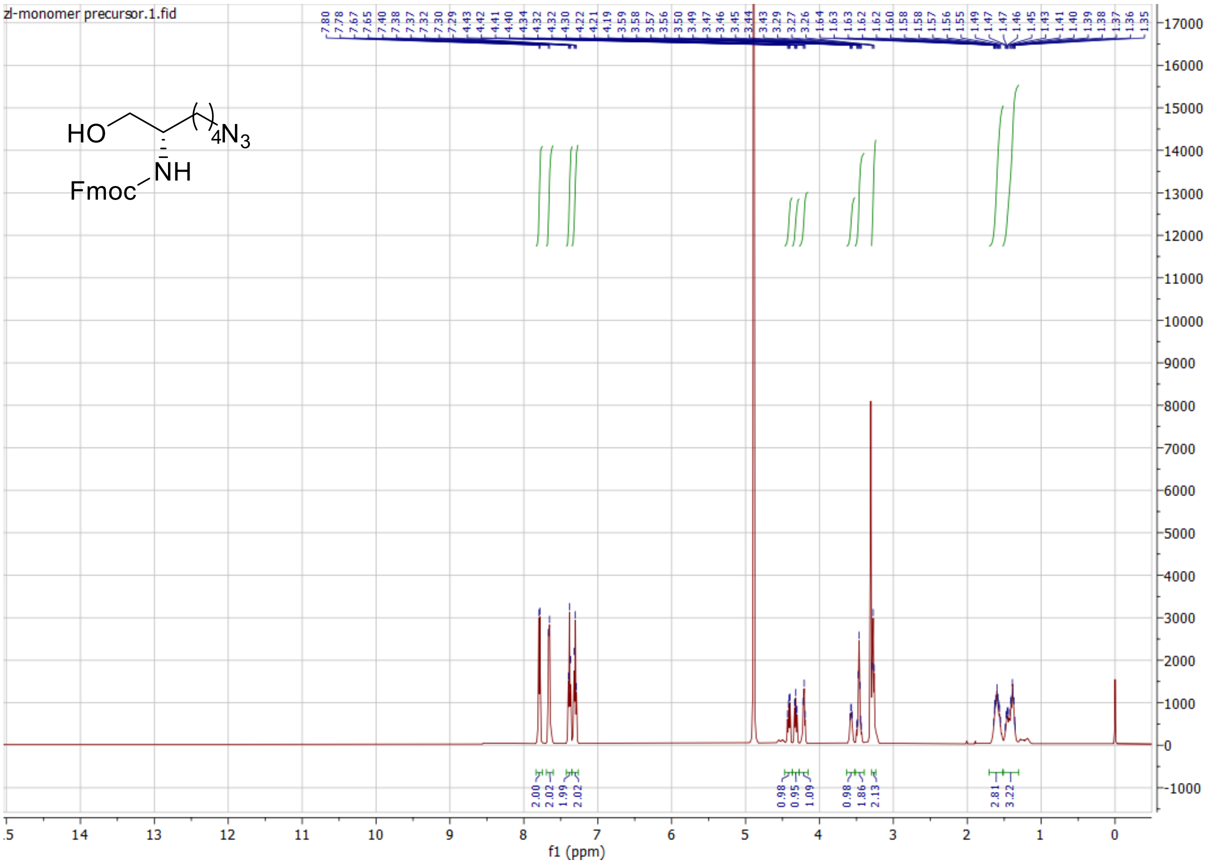
zl-monomer precursor.1.fid
HO
4N3
NH
Fmoc
2.00 2.02 1.99 2.02
0.98 0.95 1.09
0.98 1.86 2.13
2.81 3.22
f1 (ppm)

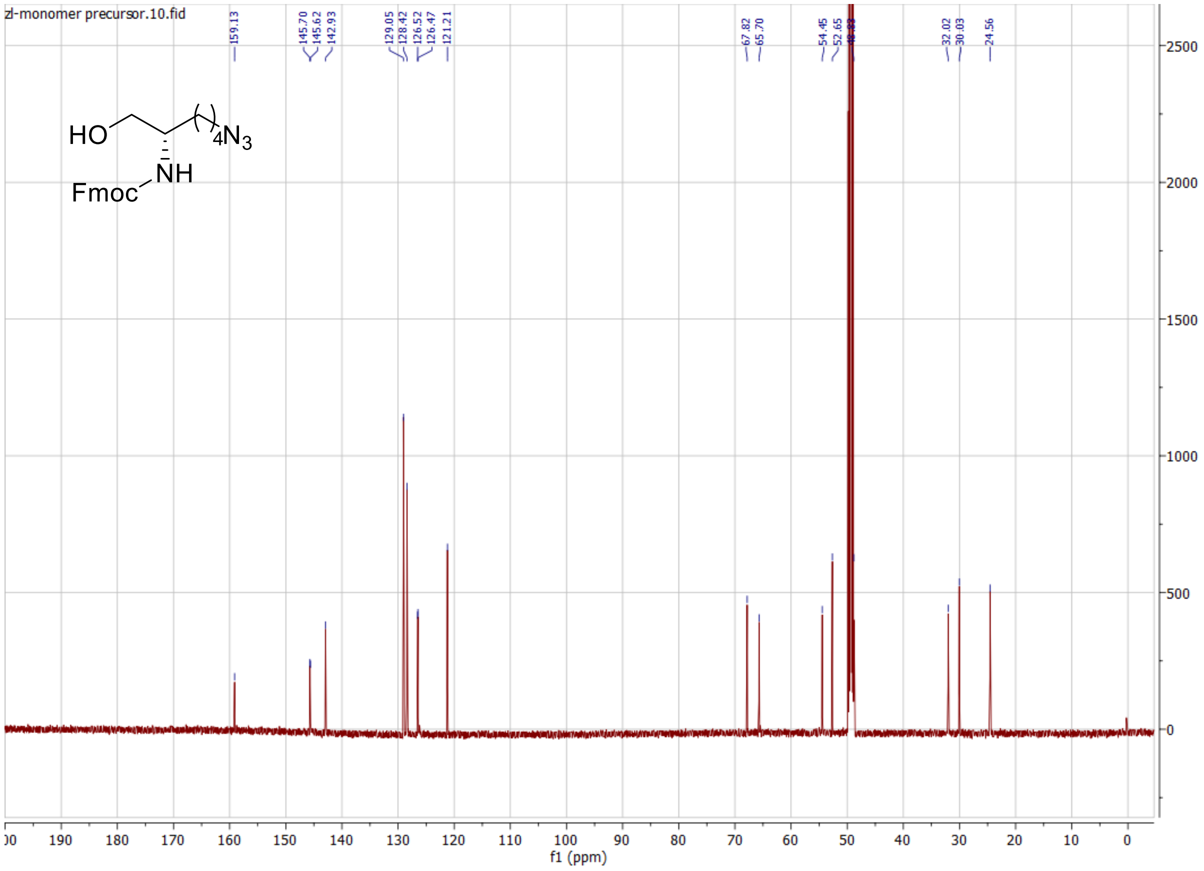
zl-monomer precursor.10.fid
159.13
145.70 145.62 142.93
129.05 128.42 126.52 126.47 121.21
67.82 65.70
54.45 52.65
32.02 30.03
24.56
HO
4N3
NH
Fmoc
f1 (ppm)

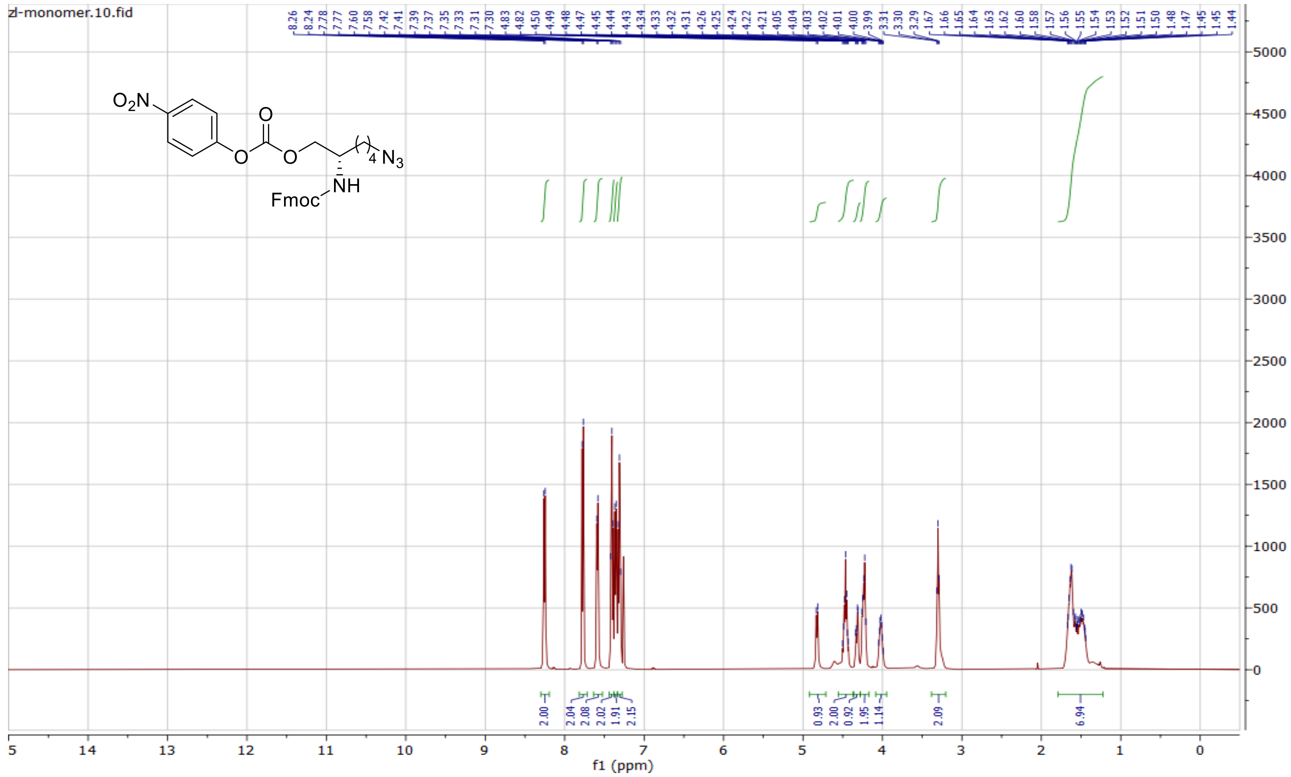
zl-monomer.10.fid
8.26 8.24 7.78 7.77 7.60 7.58 7.42 7.41 7.39 7.37 7.35 7.33 7.31 7.30 4.83 4.82 4.50 4.49 4.48 4.47 4.45 4.44 4.43 4.34 4.33 4.32 4.31 4.26 4.25 4.24 4.22 4.21 4.05 4.04 4.03 4.02 4.01 4.00 3.99 3.31 3.30 3.29 1.67 1.66 1.65 1.64 1.63 1.62 1.60 1.58 1.57 1.56 1.55 1.54 1.53 1.52 1.51 1.50 1.48 1.47 1.45 1.45 1.44
O2N
O
O
O
4
N3
NH
Fmoc
2.00 2.04 2.08 2.02 1.91 2.15 0.93 2.00 0.92 1.95 1.14 2.09 6.94
f1 (ppm)

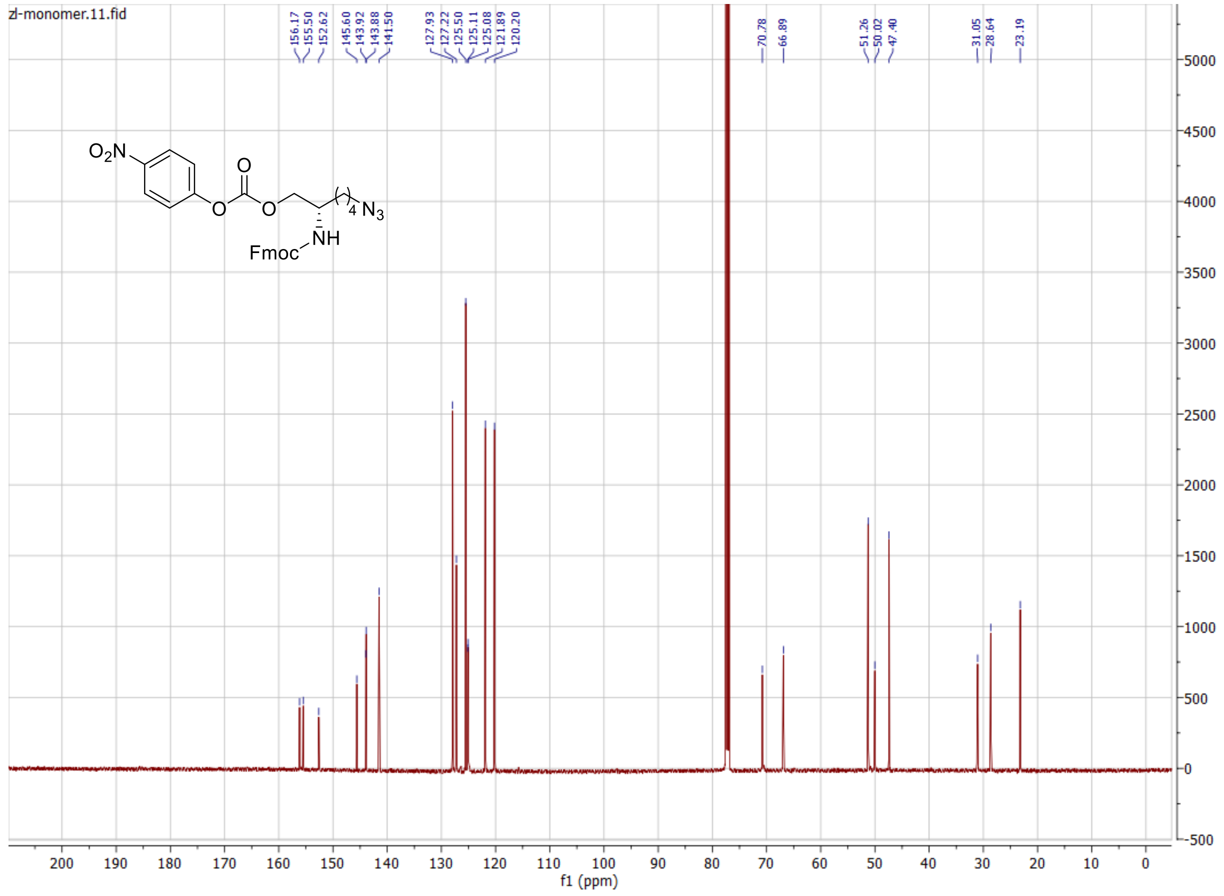
zl-monomer.11.fid
156.17 155.50 152.62 145.60 143.92 143.88 141.50 127.93 127.22 125.50 125.11 125.08 121.89 120.20 70.78 66.89 51.26 50.02 47.40 31.05 28.64 23.19
O2N
O
O
O
4
N3
NH
Fmoc
f1 (ppm)

II(b). General procedure for the solid-supported synthesis of oligourethanes

Coupling: Phenyalaninol loaded (0.44 mmol/gram, 200-400 mesh) 2-chlorotrityl polystyrene resin (40 mg, 0.0176 mmol) was added to a small fritted solid phase synthesis apparatus (5mL). The apparatus was then evacuated and backfilled with argon. The resin was suspended in 0.5 mL of anhydrous N-methyl-2-pyrrolidinone (NMP) and left to swell for 10 minutes. Next, Hunig's base (4.4 µL, 0.025 mmol) was added, followed by hydroxybenzotriazole (13.4 mg, 0.10 mmol). The suspension was swirled for 30 seconds until everything was dissolved. Finally, the activated amino alcohol **2** (33.2 mg, 0.05 mmol) was added. Reaction was shaken for 8 hours on a shaker at room temperature. Resin was washed with NMP (5x3 mL), then DCM (5x3 mL), and finally Et_2O (3x2 mL). Resin was dried overnight under vacuum. Test cleavages were effected with 1% TFA in DCM (5x0.2 mL) for 20 seconds each. Coupling efficiency was checked by LC/MS. NOTE: depending on the cleavage times and amounts of TFA used, the trifluoroacetic ester (presumably on the terminal alcohol) was observed by LC/MS. This ester was readily hydrolyzed by dissolving the sample in DCM and shaking with saturated $NaHCO_3$.

Solid phase CuAAC click chemistry: To the oligourethanes on the resin (about 40 mg resin, 0.0176 mmol) in the 5 mL fritted solid phase synthesis apparatus, a terminal alkyne (5.0 eq.), sodium ascorbate (1.0 eq.), TBTA (1.0 eq.) and copper iodide (0.5 eq.) were added along with 0.7 mL DMF. The solid phase synthesis apparatus was sealed with a septum and purged with N_2 gas for 15 minutes. After degassing, parafilm was used to seal the septum on the solid phase synthesis apparatus to prevent oxygen from going into the apparatus. The reaction was placed on the shaker and agitated for 18 hours at room temperature. The resin was then collected by vacuum filtration and washed with NMP (5x3 mL), then DCM (5x3 mL), and finally Et_2O (3x2 mL).

Deprotection: Resin loaded with terminal Fmoc-protected oligocarbamates (0.0176 mmol) suspended in 20% piperidine in DMF (3 mL) and shaken for 2 hours at room temperature. Cleavage of the dibenzofulvene-piperidine adduct was calculated by absorbance at 301 nm using Beer's Law. The resin was washed with DMF (5x3 mL), DCM (5x3 mL), Et_2O (3x2 mL), and dried overnight under vacuum.

General procedure for labelling of the terminal amine with a NBD-Fluoride (on resin): To a fritted reaction vessel was added the oligomer (dimer, trimer or tetramer) on resin (0.0176 mmol). Vessel was evacuated and backfilled with argon. The resin was suspended and swelled in anhydrous DMF (2.4 mL) for 5 minutes. Next, DIPEA (41.76 µL, 0.24 mmol) was added, followed by 4-fluoro-7-nitrobenzofurazan (33 mg, 0.179 mmol). Reaction was left to shake overnight. Resin was washed with DMF (5x16 mL), DCM (5x16 mL), and Et_2O (3x8 mL).

Cleavage procedure: Cleavages were effected with 1% TFA in DCM (5x1 mL) for 20 seconds each at room temperature. Resin was filtered off, and cleaved product was concentrated. Oligomer was purified by reverse-phase preparatory HPLC (5-95% MeCN/H_2O, 0.1% formic acid gradient elution).

II(c). Determination of Conversions of Synthesized Oligourethanes

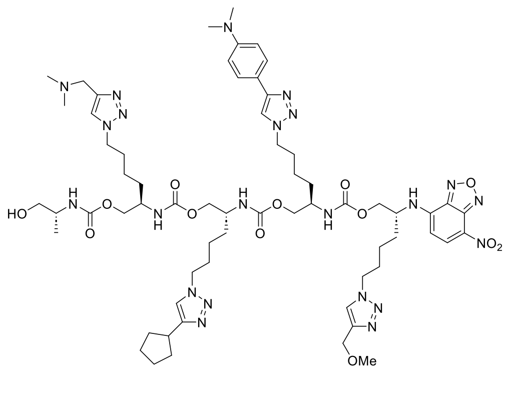

Oligomer 1: #6027

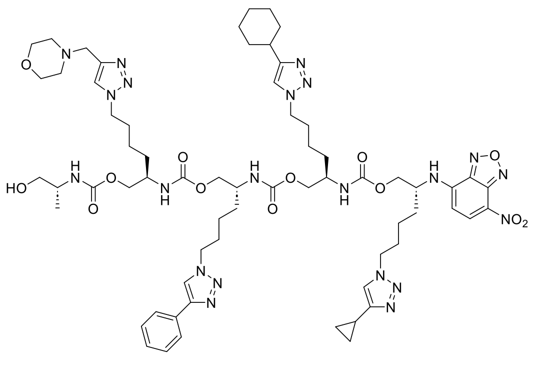

Oligomer 2: #8fd1

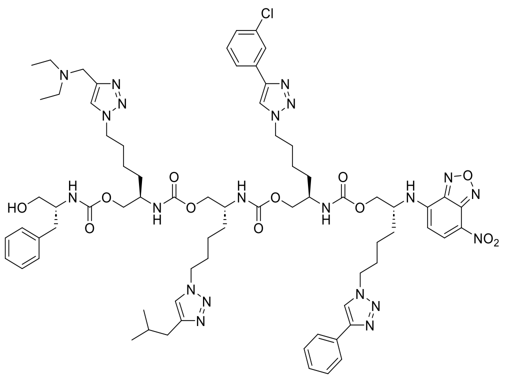

Oligomer 3: *BDRW

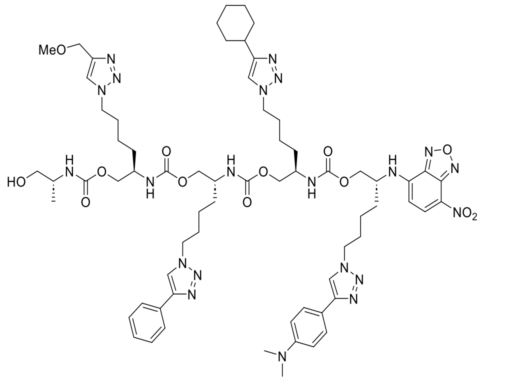

Oligomer 4: #7fd2

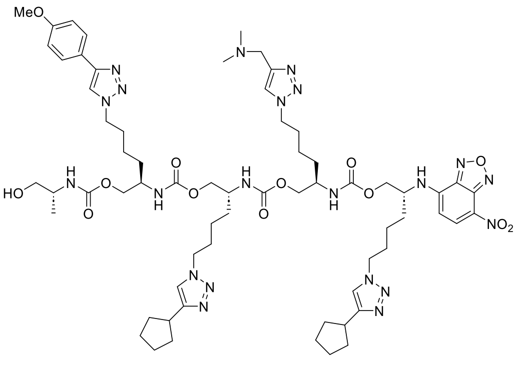

Oligomer 5: #9060

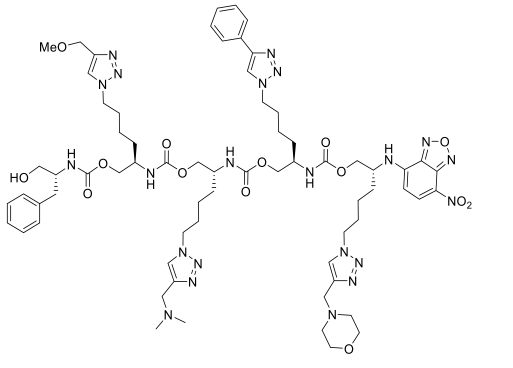

Oligomer 6: *76f8

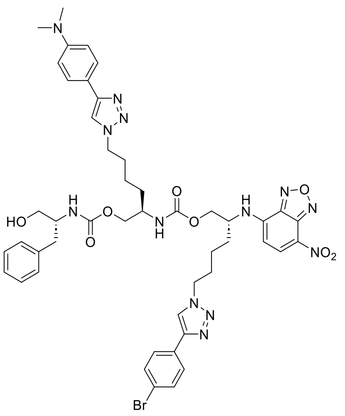

Oligomer Z1: *YI

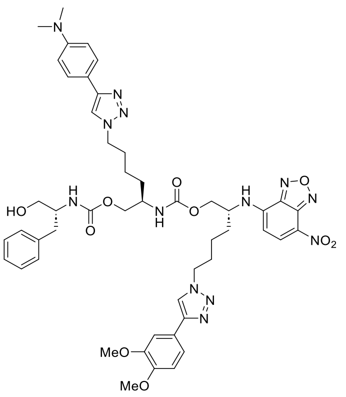

Oligomer Z2: *YT

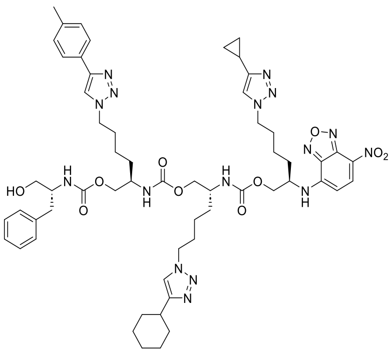

Oligomer Z3: *UMC

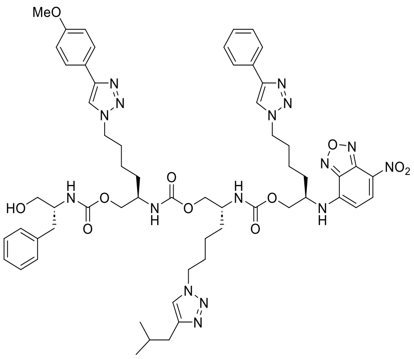

Oligomer Z4: *PDW

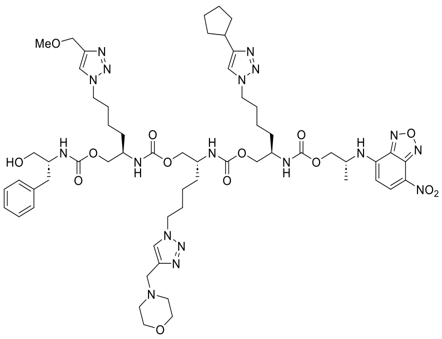

Oligomer Z5: *FLVV

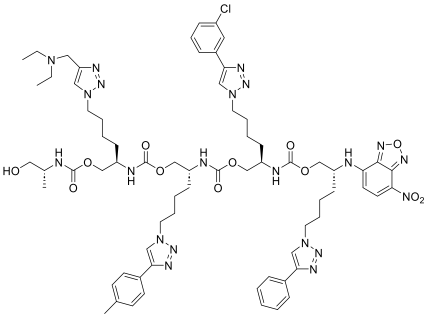

Oligomer Z6_1: #4e5f

Table 1. The conversions of 12 synthesized oligourethanes

Oligourethane	Conversion[a]	Oligourethane	Conversion[a]
1	38%	Z1	80%
2	81%	Z2	90%
3	63%	Z3	83%
4	77%	Z4	65%
5	55%	Z5	65%
6	48%	Z6	68%

[a]Conversions were determined by the ratio of the peak area of product to the total peak area in LC trace.

III. Sequencing experiments

III(a). General procedure for self-degradation (sequencing)

The sequencing procedure is adapted from previous work.[1] The oligomer (measured to be at a final concentration between 0.5-1 mM) was dissolved in methanol and added to a vial. Next, potassium phosphate tribasic monohydrate was dissolved in water and then added to the reaction solution. The final concentration of base was approximately 30 mM. The final ratio of methanol and water was 1:2.5, respectively. Before placing the vial on the heated shaker, the reaction was sampled for LC/MS by taking 50 µL of the reaction mixture and diluted into 50 µL of a 1:1 methanol: water mixture. The reaction was ramped quickly to 70 °C in the heated shaker and held at the temperature. The reaction was sampled every 60 minutes for 240 minutes.

III(b). Oligomer Sequencing Data:

Oligomer 1: #6027

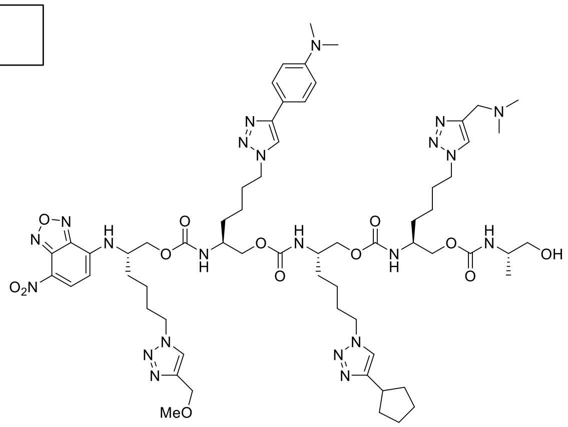

Exact Mass: 1366.74

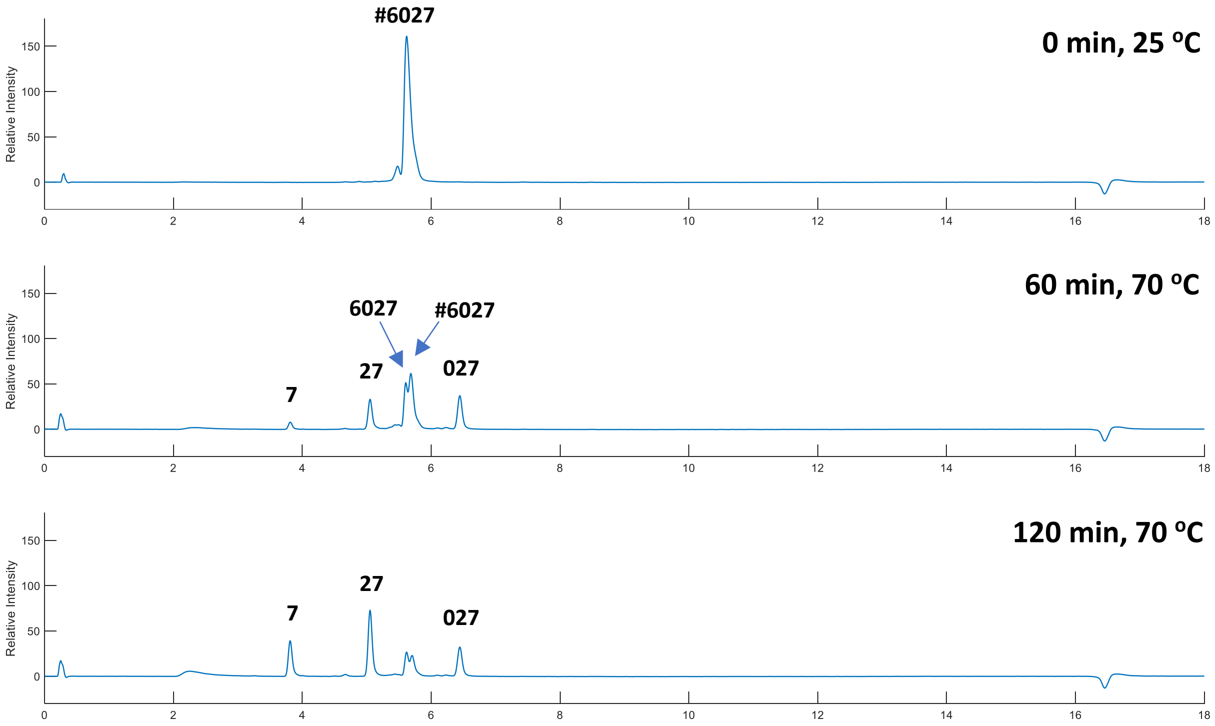

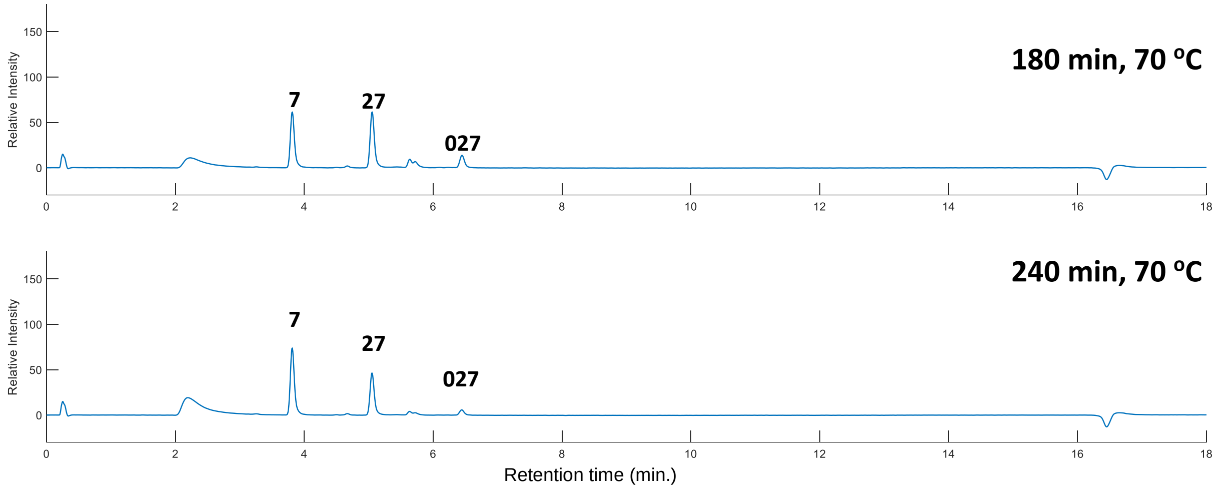

After 120 minutes of sequencing, all the following molecules are observed in the solution and analyzed via High-Res MS.

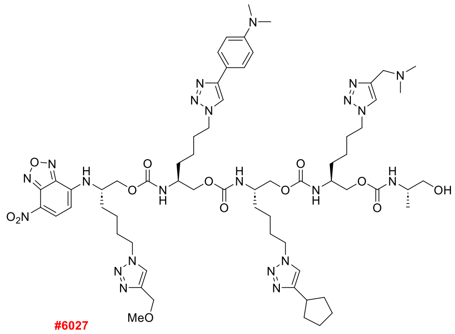

#6027

Chemical Formula: $C_{63}H_{94}N_{22}O_{13}$
Exact Mass: 1366.74

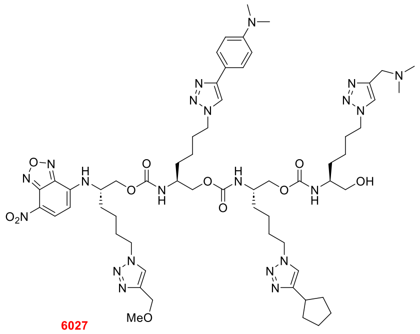

6027

Chemical Formula: $C_{59}H_{87}N_{21}O_{11}$
Exact Mass: 1265.69

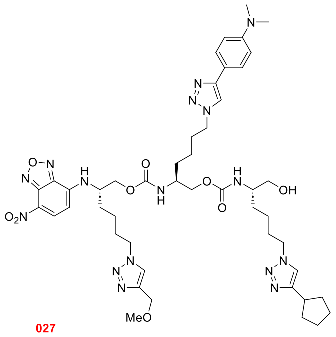

027

Chemical Formula: $C_{47}H_{66}N_{16}O_{9}$
Exact Mass: 998.52

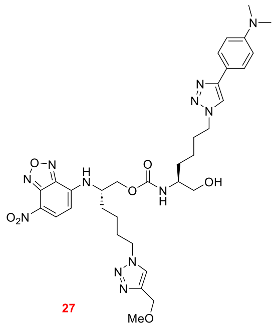

27

Chemical Formula: $C_{33}H_{44}N_{12}O_{7}$
Exact Mass: 720.35

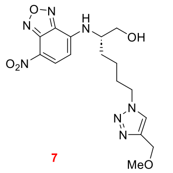

7

Chemical Formula: $C_{16}H_{21}N_{7}O_{5}$
Exact Mass: 391.16

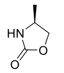

Chemical Formula: $C_{4}H_{7}NO_{2}$
Exact Mass: 101.05

#

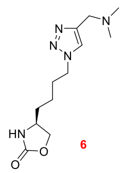

6

Chemical Formula: $C_{12}H_{21}N_{5}O_{2}$
Exact Mass: 267.17

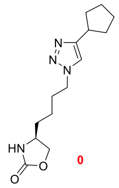

0

Chemical Formula: $C_{14}H_{22}N_{4}O_{2}$
Exact Mass: 278.17

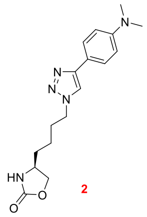

2

Chemical Formula: $C_{17}H_{23}N_{5}O_{2}$
Exact Mass: 329.19

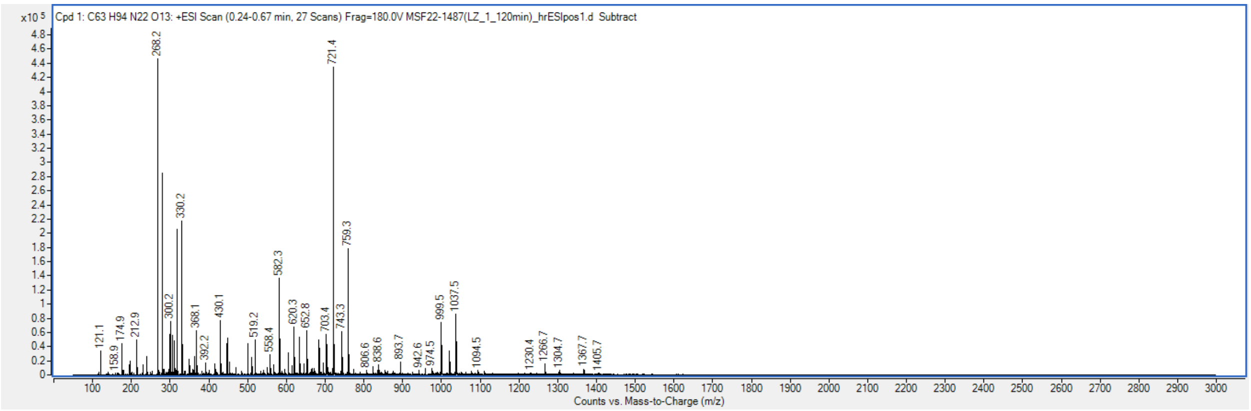

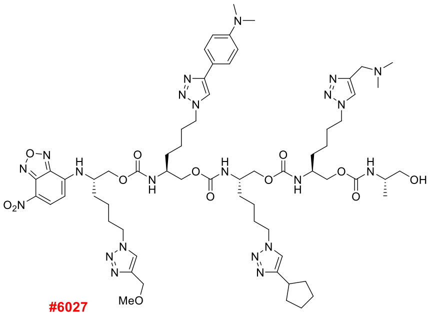

#6027

Chemical Formula: $C_{63}H_{94}N_{22}O_{13}$
Exact Mass: 1366.74

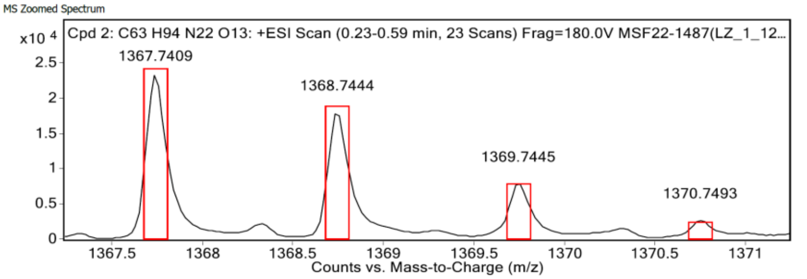

MS Spectrum Peak List

Obs. m/z	Calc. m/z	Charge	Abundance	Formula	Ion Species	Tgt Mass Error (ppm)
268.1767			1041490			
1367.7409	1367.7443	1	23400	C63H94N22O13	(M+H)+	2.52
1368.7444	1368.7471	1	18146	C63H94N22O13	(M+H)+	1.94
1369.7445	1369.7497	1	8036	C63H94N22O13	(M+H)+	3.83
1370.7493	1370.7523	1	2845	C63H94N22O13	(M+H)+	2.22
1371.7174	1371.7548	1	1251	C63H94N22O13	(M+H)+	27.26

--- End Of Report ---

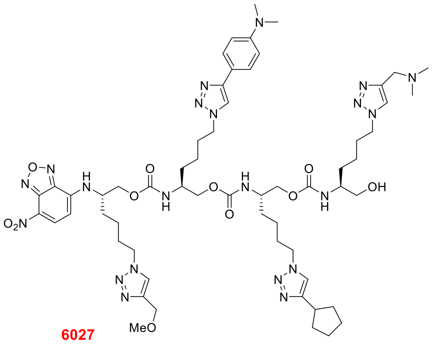

6027

Chemical Formula: $C_{59}H_{87}N_{21}O_{11}$
Exact Mass: 1265.69

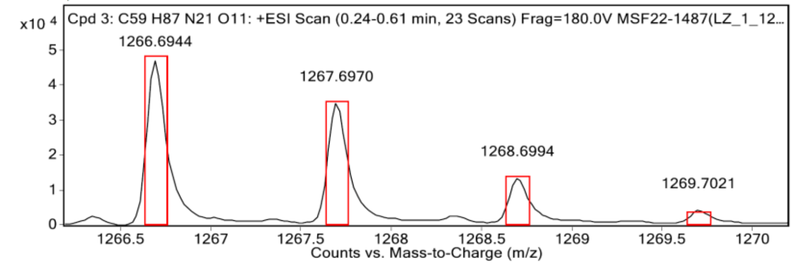

MS Spectrum Peak List

Obs. m/z	Calc. m/z	Charge	Abundance	Formula	Ion Species	Tgt Mass Error (ppm)
268.1767			1053964			
1266.6944	1266.6967	1	47200	C59H87N21O11	(M+H)+	1.78
1267.6970	1267.6994	1	35159	C59H87N21O11	(M+H)+	1.93
1268.6994	1268.7020	1	13714	C59H87N21O11	(M+H)+	2.07
1269.7021	1269.7046	1	4512	C59H87N21O11	(M+H)+	1.98
1270.7107	1270.7071	1	1400	C59H87N21O11	(M+H)+	-2.81

--- End Of Report ---

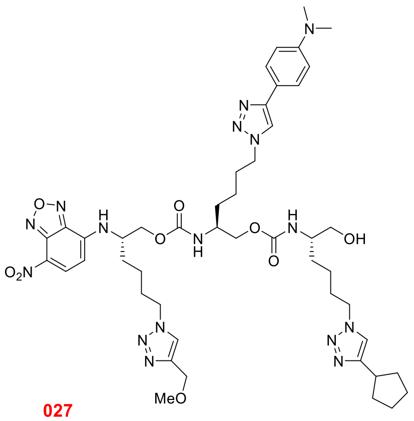

027

Chemical Formula: $C_{47}H_{66}N_{16}O_9$
Exact Mass: 998.52

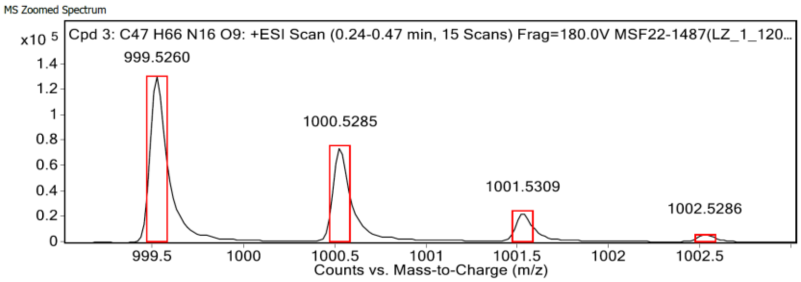

MS Spectrum Peak List

Obs. m/z	Calc. m/z	Charge	Abundance	Formula	Ion Species	Tgt Mass Error (ppm)
721.3529			771717			
999.5260	999.5271	1	130544	C47H66N16O9	(M+H)+	1.18
1000.5285	1000.5299	1	74346	C47H66N16O9	(M+H)+	1.37
1001.5309	1001.5325	1	23107	C47H66N16O9	(M+H)+	1.63
1002.5286	1002.5351	1	5701	C47H66N16O9	(M+H)+	6.43
1003.5350	1003.5376	1	1579	C47H66N16O9	(M+H)+	2.57

--- End Of Report ---

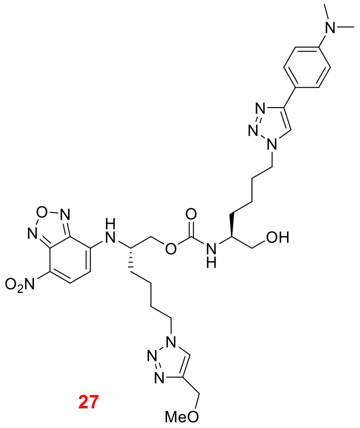

27

Chemical Formula: $C_{33}H_{44}N_{12}O_7$
Exact Mass: 720.35

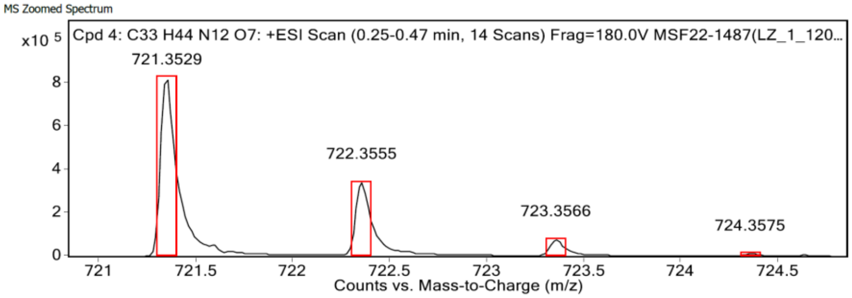

MS Spectrum Peak List

Obs. m/z	Calc. m/z	Charge	Abundance	Formula	Ion Species	Tgt Mass Error (ppm)
721.3529	721.3529	1	824867	C33H44N12O7	(M+H)+	-0.09
722.3555	722.3556	1	343106	C33H44N12O7	(M+H)+	0.11
723.3566	723.3581	1	78562	C33H44N12O7	(M+H)+	2.05
724.3575	724.3606	1	12158	C33H44N12O7	(M+H)+	4.25
725.3664	725.3630	1	1865	C33H44N12O7	(M+H)+	-4.7
726.3640	726.3654	1	489	C33H44N12O7	(M+H)+	1.81

--- End Of Report ---

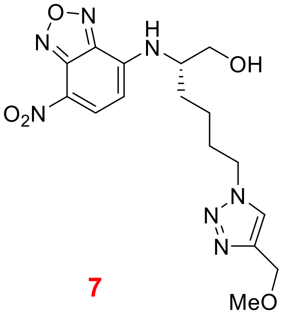

7

Chemical Formula: $C_{16}H_{21}N_7O_5$
Exact Mass: 391.16

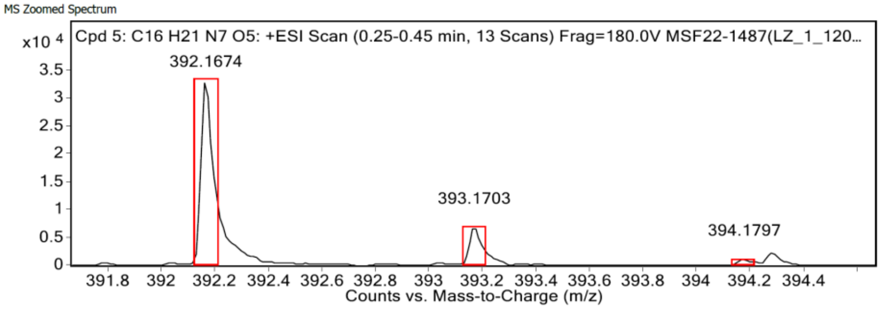

MS Spectrum Peak List

Obs. m/z	Calc. m/z	Charge	Abundance	Formula	Ion Species	Tgt Mass Error (ppm)
392.1674	392.1677	1	33332	C16H21N7O5	(M+H)+	0.65
393.1703	393.1703	1	6838	C16H21N7O5	(M+H)+	-0.01
394.1797	394.1725	1	1093	C16H21N7O5	(M+H)+	-18.26
721.3529			875111			

--- End Of Report ---

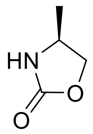

Chemical Formula: $C_4H_7NO_2$
Exact Mass: 101.05

#

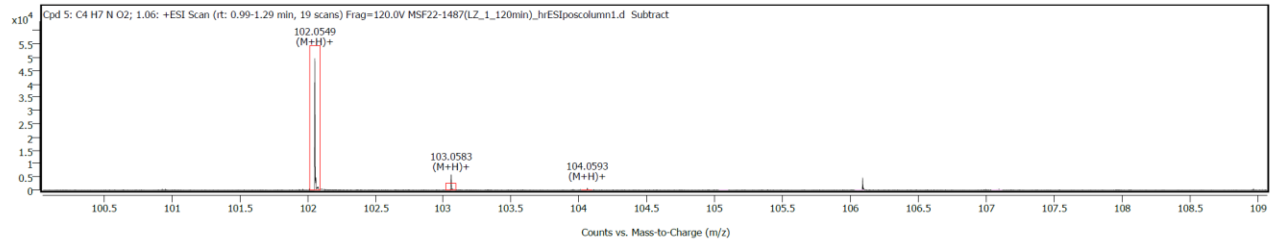

Spectrum Peaks

Obs. m/z	Calc. m/z	Charge	Abund	Formula	Ion Species	Tgt Mass Error (PPM)
102.0549	102.0550	1	50756	C4H7NO2	(M+H)+	-0.76
103.0583	103.0579	1	5789	C4H7NO2	(M+H)+	3.83
104.0593	104.0595	1	629	C4H7NO2	(M+H)+	-2.17

MassHunter Qual 10.0
(End of Report)

MS Zoomed Spectrum

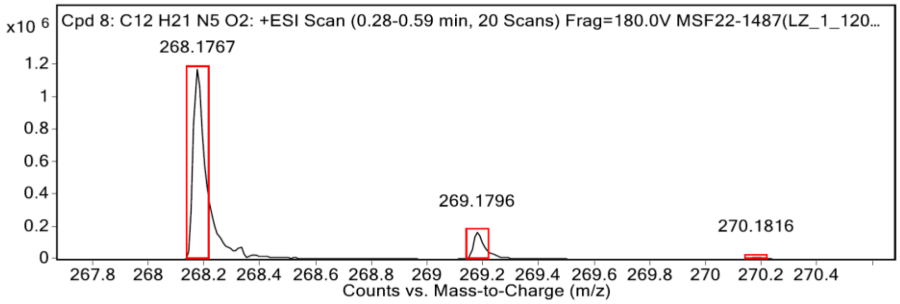

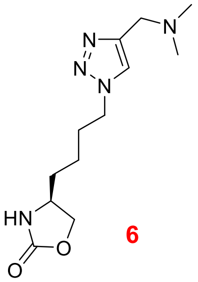

6

Chemical Formula: $C_{12}H_{21}N_5O_2$
Exact Mass: 267.17

MS Spectrum Peak List

Obs. m/z	Calc. m/z	Charge	Abundance	Formula	Ion Species	Tgt Mass Error (ppm)
268.1767	268.1768	1	1189627	C12H21N5O2	(M+H)+	0.35
269.1796	269.1794	1	172121	C12H21N5O2	(M+H)+	-0.64
270.1816	270.1818	1	16955	C12H21N5O2	(M+H)+	0.49
271.1826	271.1841	1	1973	C12H21N5O2	(M+H)+	5.51

--- End Of Report ---

MS Zoomed Spectrum

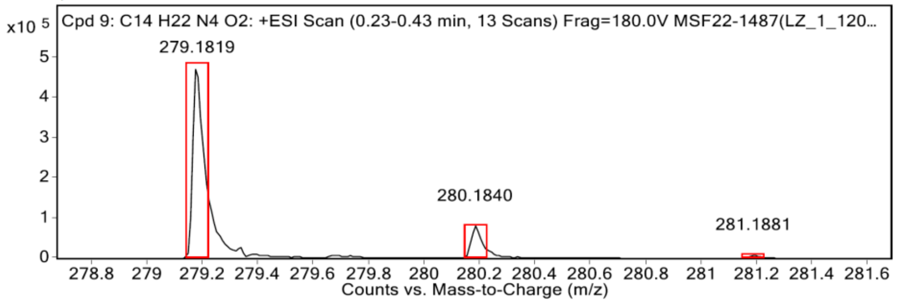

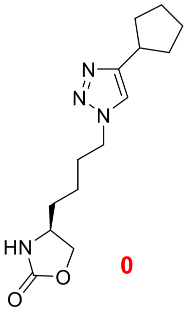

0

Chemical Formula: $C_{14}H_{22}N_4O_2$
Exact Mass: 278.17

MS Spectrum Peak List

Obs. m/z	Calc. m/z	Charge	Abundance	Formula	Ion Species	Tgt Mass Error (ppm)
268.1766			990696			
279.1819	279.1816	1	485039	C14H22N4O2	(M+H)+	-1.17
280.1840	280.1844	1	81153	C14H22N4O2	(M+H)+	1.49
281.1881	281.1869	1	8885	C14H22N4O2	(M+H)+	-4.25

--- End Of Report ---

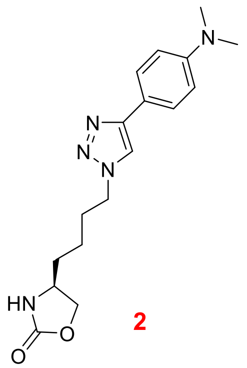

2

Chemical Formula: $C_{17}H_{23}N_5O_2$
Exact Mass: 329.19

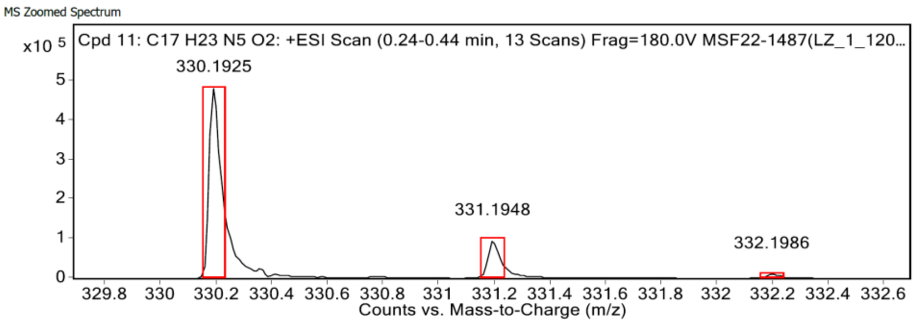

MS Spectrum Peak List

Obs. m/z	Calc. m/z	Charge	Abundance	Formula	Ion Species	Tgt Mass Error (ppm)
268.1766			1147487			
330.1925	330.1925	1	483892	C17H23N5O2	(M+H)+	-0.29
331.1948	331.1953	1	97276	C17H23N5O2	(M+H)+	1.41
332.1986	332.1979	1	12985	C17H23N5O2	(M+H)+	-2.29

--- End Of Report ---

Oligomer 2: #8fd1

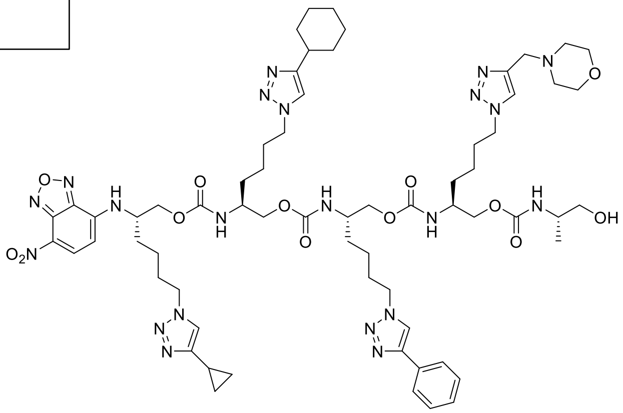

Exact Mass: 1375.73

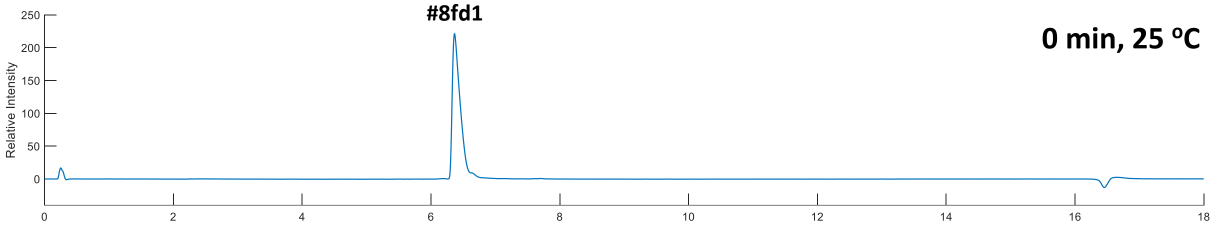

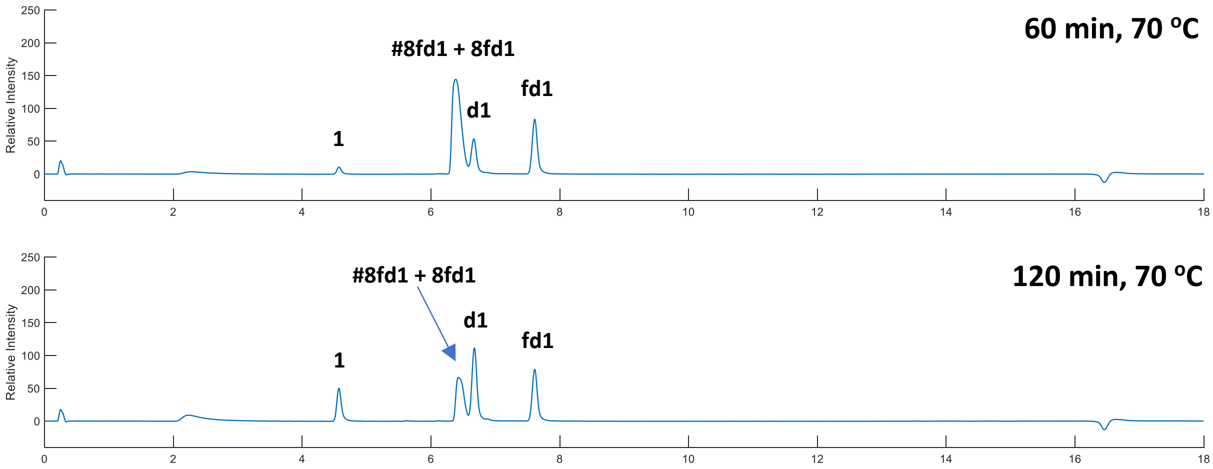

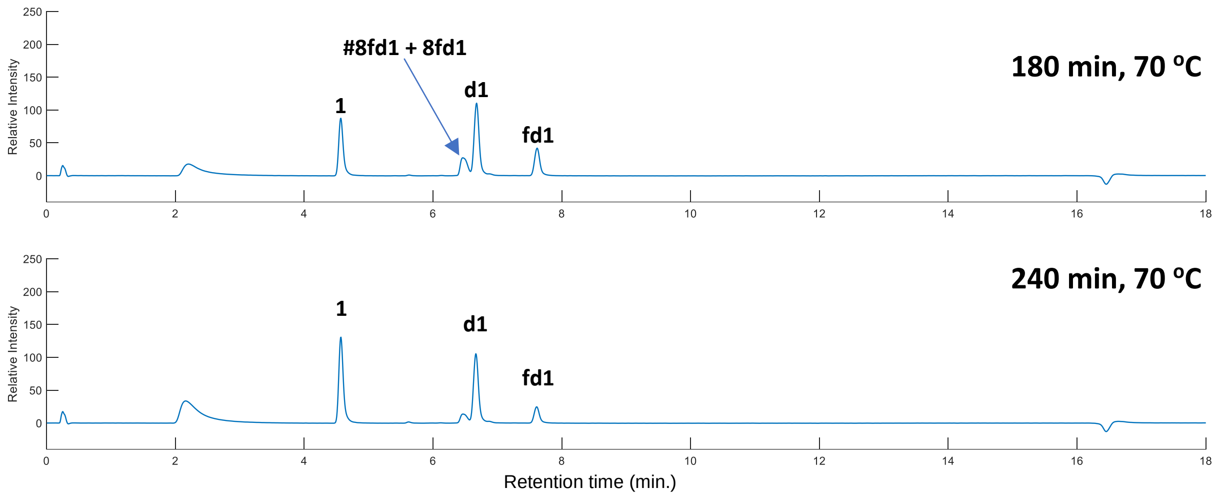

After 120 minutes of sequencing, all the following molecules are observed in the solution and analyzed via High-Res MS.

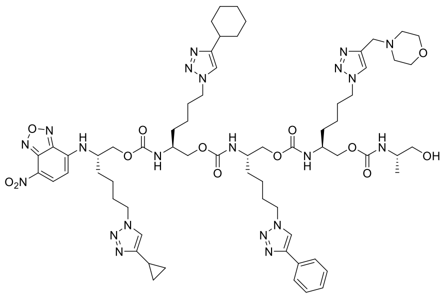

#8fd1 Chemical Formula: $C_{65}H_{93}N_{21}O_{13}$
Exact Mass: 1375.73

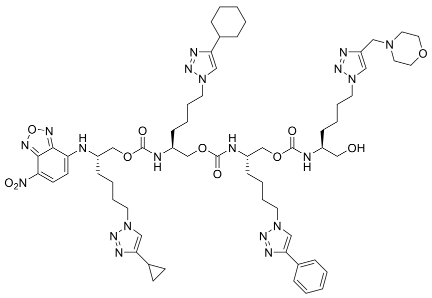

8fd1 Chemical Formula: $C_{61}H_{86}N_{20}O_{11}$
Exact Mass: 1274.68

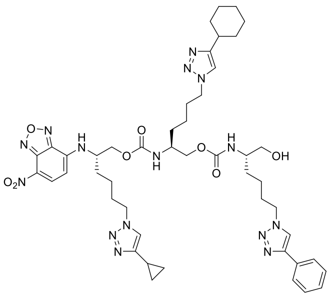

fd1 Chemical Formula: $C_{47}H_{63}N_{15}O_{8}$
Exact Mass: 965.50

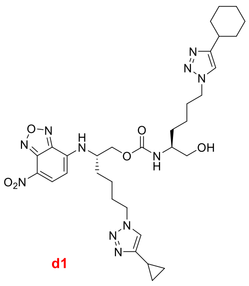

d1

Chemical Formula: $C_{32}H_{45}N_{11}O_{6}$
Exact Mass: 679.36

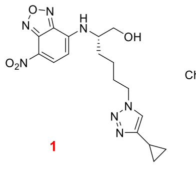

1

Chemical Formula: $C_{17}H_{21}N_{7}O_{4}$
Exact Mass: 387.17

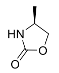

Chemical Formula: $C_{4}H_{7}NO_{2}$
Exact Mass: 101.05

#

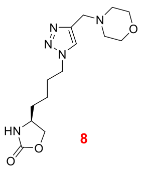

8

Chemical Formula: $C_{14}H_{23}N_{5}O_{3}$
Exact Mass: 309.18

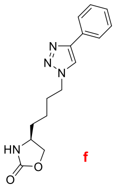

f

Chemical Formula: $C_{15}H_{18}N_{4}O_{2}$
Exact Mass: 286.14

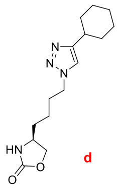

d

Chemical Formula: $C_{15}H_{24}N_{4}O_{2}$
Exact Mass: 292.19

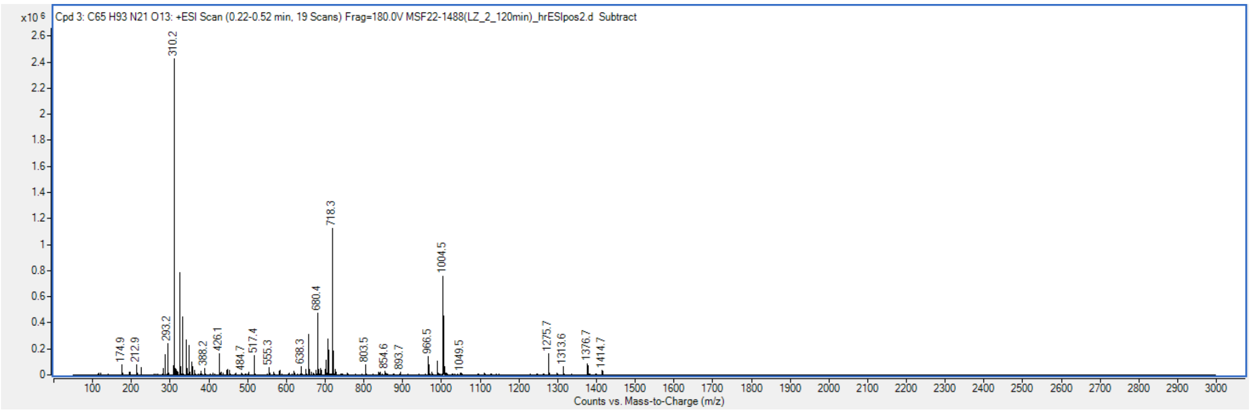

MS Zoomed Spectrum

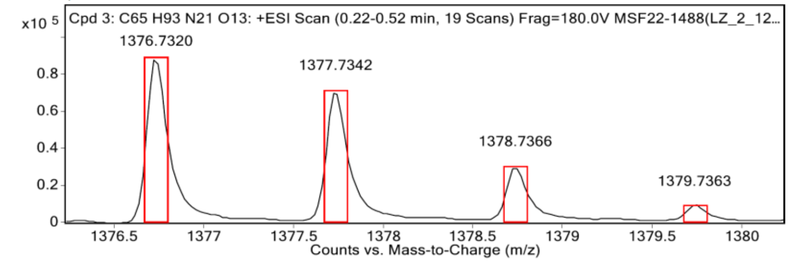

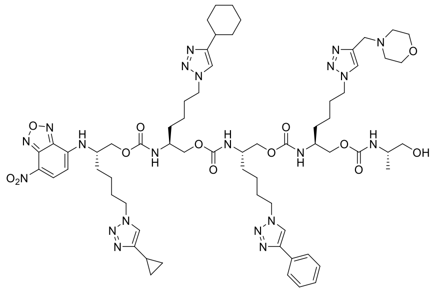

#8fd1 Chemical Formula: $C_{65}H_{93}N_{21}O_{13}$
Exact Mass: 1375.73

MS Spectrum Peak List

Obs. m/z	Calc. m/z	Charge	Abundance	Formula	Ion Species	Tgt Mass Error (ppm)
310.1872			2446754			
1376.7320	1376.7334	1	89269	C65H93N21O13	(M+H)+	1.03
1377.7342	1377.7362	1	71284	C65H93N21O13	(M+H)+	1.52
1378.7366	1378.7389	1	30031	C65H93N21O13	(M+H)+	1.68
1379.7363	1379.7415	1	8537	C65H93N21O13	(M+H)+	3.78
1380.7370	1380.7441	1	2244	C65H93N21O13	(M+H)+	5.15
1381.7188	1381.7466	1	769	C65H93N21O13	(M+H)+	20.12

--- End Of Report ---

MS Zoomed Spectrum

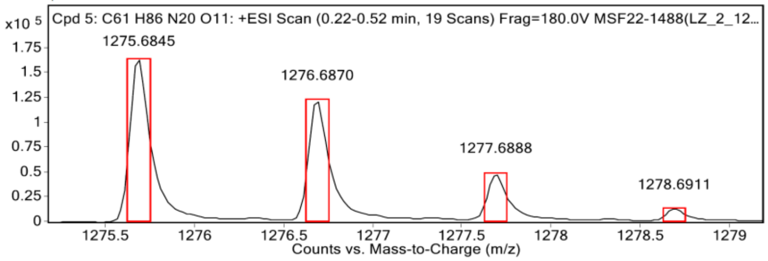

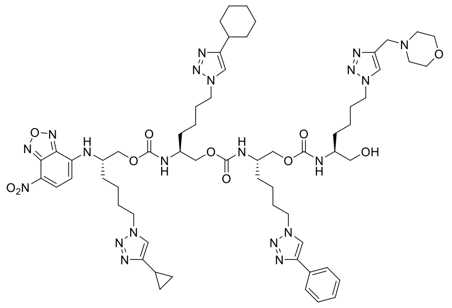

8fd1 Chemical Formula: $C_{61}H_{86}N_{20}O_{11}$
Exact Mass: 1274.68

MS Spectrum Peak List

Obs. m/z	Calc. m/z	Charge	Abundance	Formula	Ion Species	Tgt Mass Error (ppm)
310.1872			2446588			
1275.6845	1275.6858	1	164259	C61H86N20O11	(M+H)+	0.99
1276.6870	1276.6886	1	121823	C61H86N20O11	(M+H)+	1.24
1277.6888	1277.6912	1	48473	C61H86N20O11	(M+H)+	1.89
1278.6911	1278.6938	1	11885	C61H86N20O11	(M+H)+	2.15
1279.6918	1279.6964	1	3160	C61H86N20O11	(M+H)+	3.58
1280.6949	1280.6989	1	757	C61H86N20O11	(M+H)+	3.09

--- End Of Report ---

MS Zoomed Spectrum

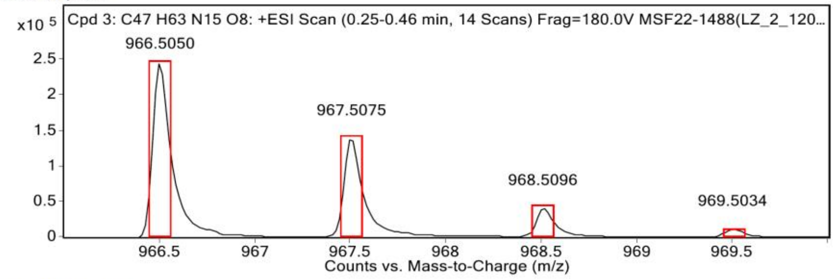

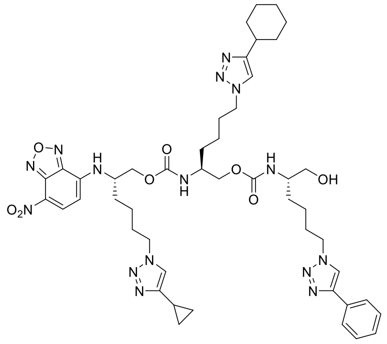

fd1 Chemical Formula: $C_{47}H_{63}N_{15}O_{8}$
Exact Mass: 965.50

MS Spectrum Peak List

Obs. m/z	Calc. m/z	Charge	Abundance	Formula	Ion Species	Tgt Mass Error (ppm)
310.1870			2305930			
966.5050	966.5057	1	246291	C47H63N15O8	(M+H)+	0.66
967.5075	967.5085	1	140196	C47H63N15O8	(M+H)+	1.02
968.5096	968.5111	1	41736	C47H63N15O8	(M+H)+	1.62
969.5034	969.5137	1	12513	C47H63N15O8	(M+H)+	10.61
970.4985	970.5162	1	4219	C47H63N15O8	(M+H)+	18.32

--- End Of Report ---

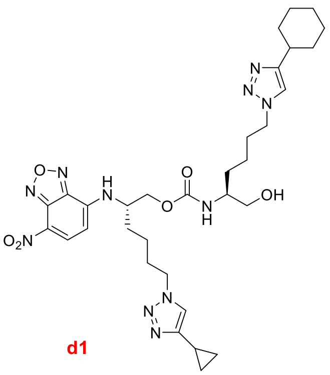

d1

Chemical Formula: $C_{32}H_{45}N_{11}O_6$
Exact Mass: 679.36

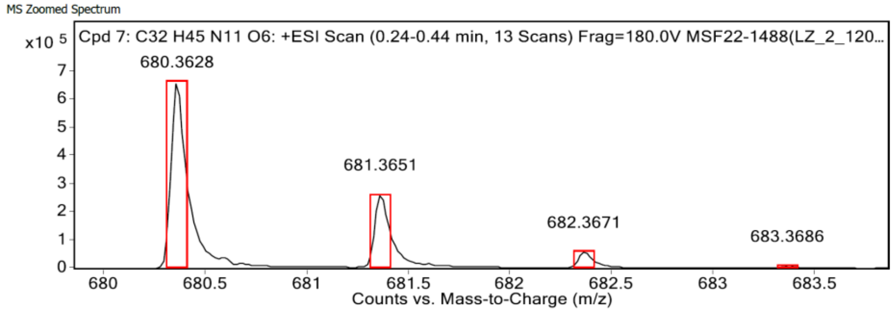

MS Spectrum Peak List

Obs. m/z	Calc. m/z	Charge	Abundance	Formula	Ion Species	Tgt Mass Error (ppm)
310.1870			3191834			
680.3628	680.3627	1	661522	C32H45N11O6	(M+H)+	-0.07
681.3651	681.3655	1	262285	C32H45N11O6	(M+H)+	0.45
682.3671	682.3680	1	57962	C32H45N11O6	(M+H)+	1.35
683.3686	683.3705	1	8507	C32H45N11O6	(M+H)+	2.72

--- End Of Report ---

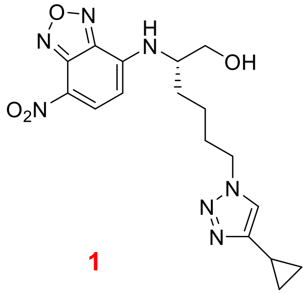

1

Chemical Formula: $C_{17}H_{21}N_7O_4$
Exact Mass: 387.17

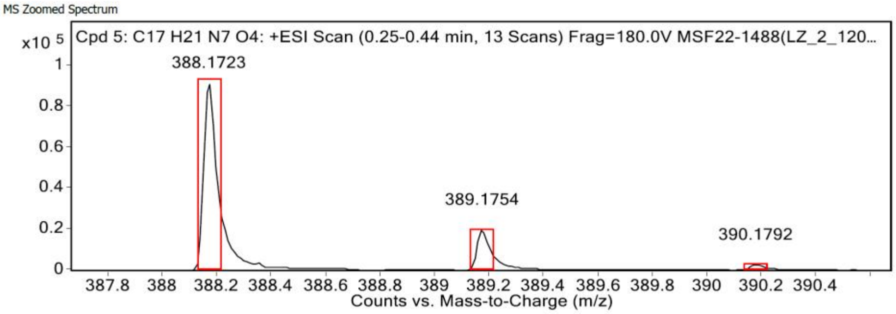

MS Spectrum Peak List

Obs. m/z	Calc. m/z	Charge	Abundance	Formula	Ion Species	Tgt Mass Error (ppm)
310.1870			2367508			
388.1723	388.1728	1	92331	C17H21N7O4	(M+H)+	1.2
389.1754	389.1754	1	20048	C17H21N7O4	(M+H)+	-0.07
390.1792	390.1777	1	2938	C17H21N7O4	(M+H)+	-3.77

--- End Of Report ---

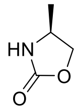

Chemical Formula: $C_4H_7NO_2$
Exact Mass: 101.05

#

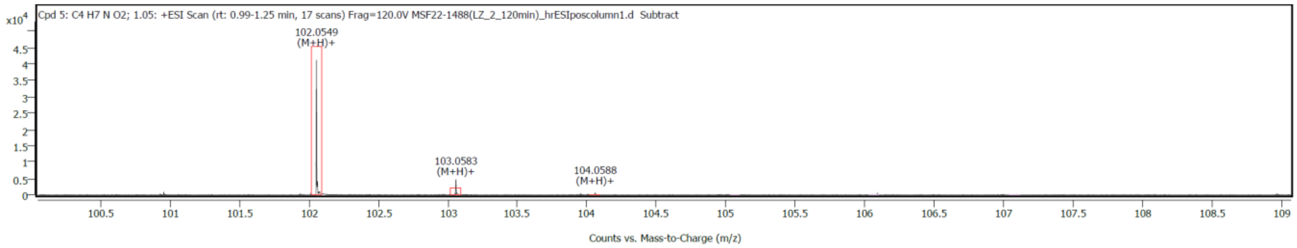

Spectrum Peaks

Obs. m/z	Calc. m/z	Charge	Abund	Formula	Ion Species	Tgt Mass Error (PPM)
102.0549	102.0550	1	42443	C4H7NO2	(M+H)+	-0.82
103.0583	103.0579	1	4632	C4H7NO2	(M+H)+	3.39
104.0588	104.0595	1	531	C4H7NO2	(M+H)+	-6.40

MassHunter Qual 10.0
(End of Report)

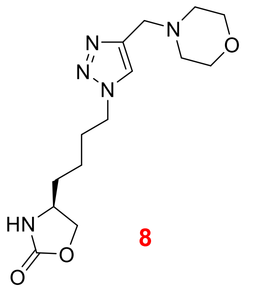

8

Chemical Formula: $C_{14}H_{23}N_5O_3$
Exact Mass: 309.18

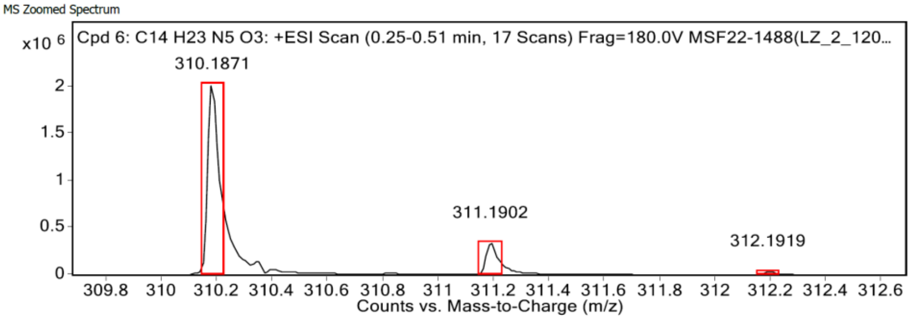

MS Spectrum Peak List

Obs. m/z	Calc. m/z	Charge	Abundance	Formula	Ion Species	Tgt Mass Error (ppm)
310.1871	310.1874	1	2034225	C14H23N5O3	(M+H)+	0.78
311.1902	311.1901	1	341235	C14H23N5O3	(M+H)+	-0.45
312.1919	312.1924	1	39422	C14H23N5O3	(M+H)+	1.67
313.1938	313.1948	1	3118	C14H23N5O3	(M+H)+	3.32

--- End Of Report ---

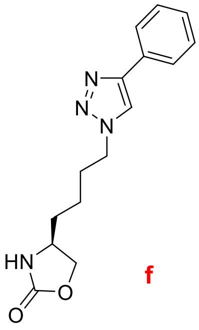

f

Chemical Formula: $C_{15}H_{18}N_4O_2$
Exact Mass: 286.14

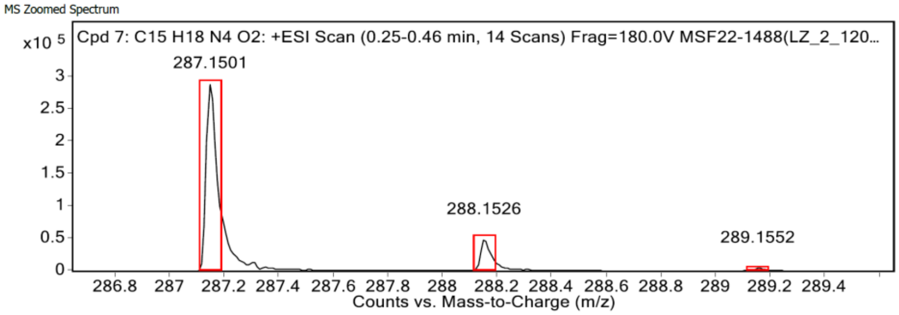

MS Spectrum Peak List

Obs. m/z	Calc. m/z	Charge	Abundance	Formula	Ion Species	Tgt Mass Error (ppm)
287.1501	287.1503	1	293256	C15H18N4O2	(M+H)+	0.57
288.1526	288.1531	1	50002	C15H18N4O2	(M+H)+	1.97
289.1552	289.1557	1	5938	C15H18N4O2	(M+H)+	1.7
290.1221	290.1581	1	333	C15H18N4O2	(M+H)+	124.14
310.1870			2299386			

--- End Of Report ---

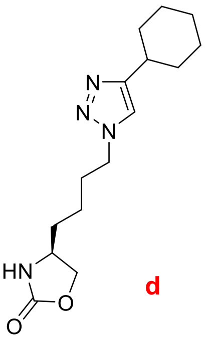

d

Chemical Formula: $C_{15}H_{24}N_4O_2$
Exact Mass: 292.19

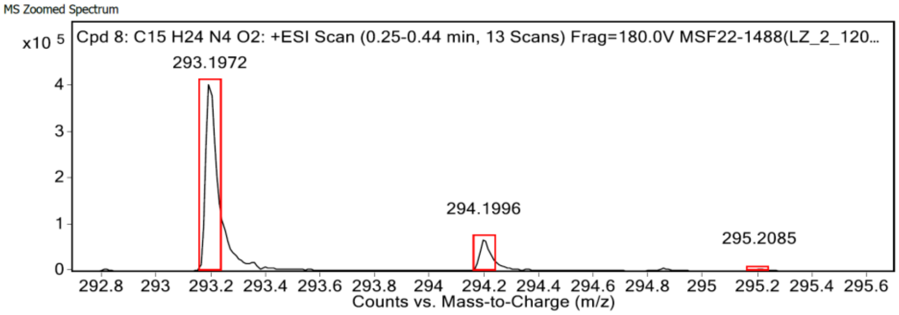

MS Spectrum Peak List

Obs. m/z	Calc. m/z	Charge	Abundance	Formula	Ion Species	Tgt Mass Error (ppm)
293.1972	293.1972	1	412036	C15H24N4O2	(M+H)+	-0.03
294.1996	294.2001	1	69917	C15H24N4O2	(M+H)+	1.67
295.2085	295.2026	1	5729	C15H24N4O2	(M+H)+	-19.74
296.1461	296.2051	1	370	C15H24N4O2	(M+H)+	199.05
310.1870			2368973			

--- End Of Report ---

Oligomer 3: #bdrw

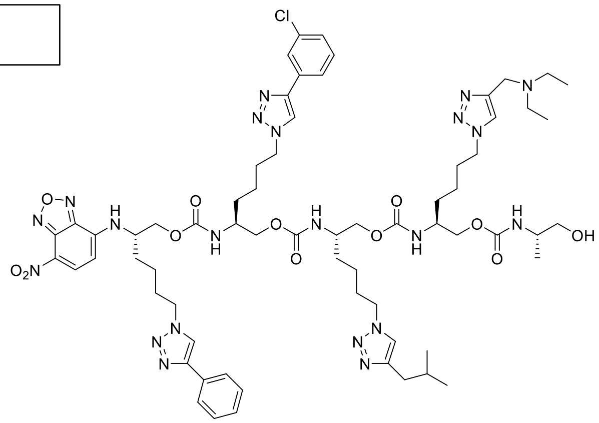

Exact Mass: 1405.69

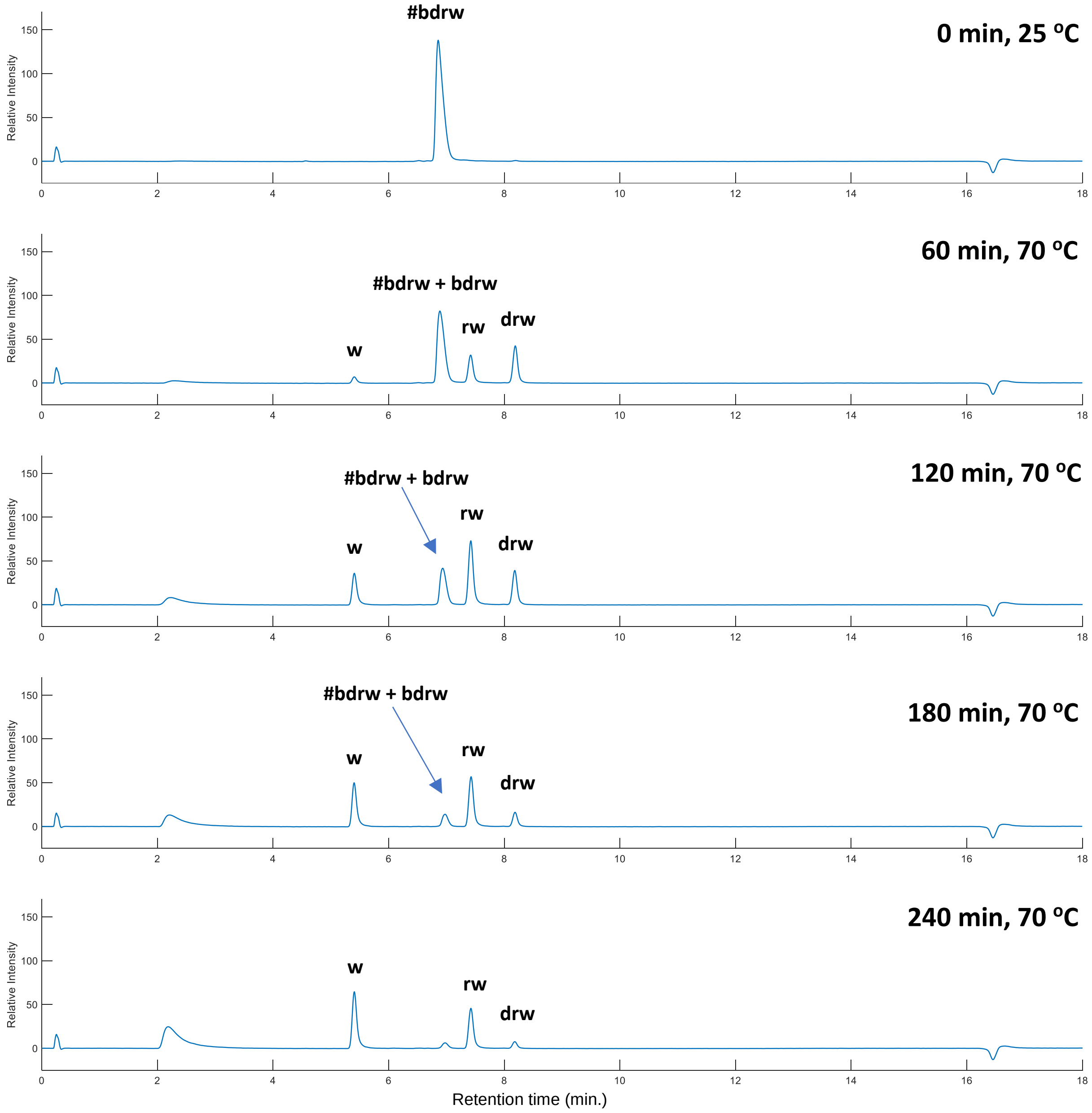

After 120 minutes of sequencing, all the following molecules are observed in the solution and analyzed via High-Res MS.

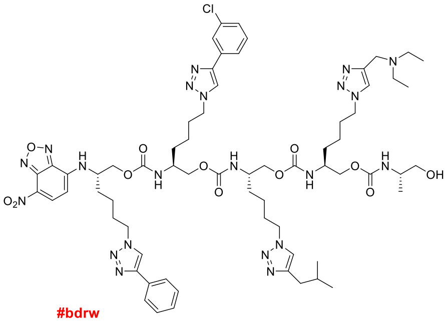

#bdrw

Chemical Formula: $C_{66}H_{92}ClN_{21}O_{12}$
Exact Mass: 1405.69

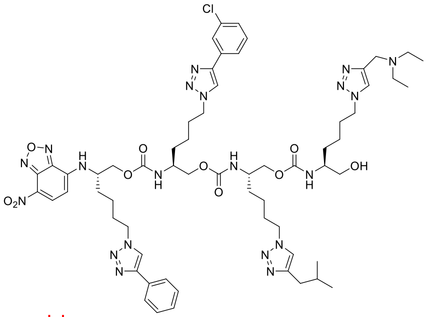

bdrw

Chemical Formula: $C_{62}H_{85}ClN_{20}O_{10}$
Exact Mass: 1304.64

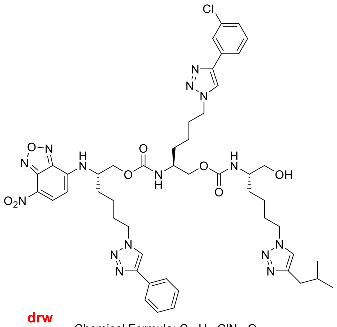

drw

Chemical Formula: $C_{48}H_{60}ClN_{15}O_{8}$
Exact Mass: 1009.44

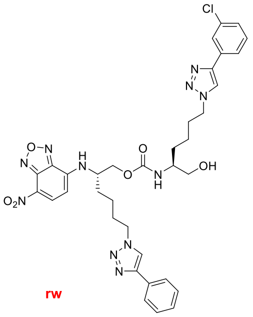

rw

Chemical Formula: $C_{35}H_{38}ClN_{11}O_{6}$
Exact Mass: 743.27

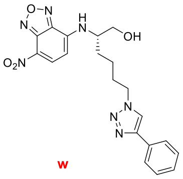

w

Chemical Formula: $C_{20}H_{21}N_{7}O_{4}$
Exact Mass: 423.17

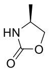

Chemical Formula: $C_4H_7NO_2$
Exact Mass: 101.05

#

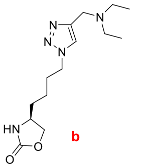

b

Chemical Formula: $C_{14}H_{25}N_{5}O_{2}$
Exact Mass: 295.20

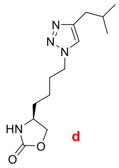

d

Chemical Formula: $C_{13}H_{22}N_{4}O_{2}$
Exact Mass: 266.17

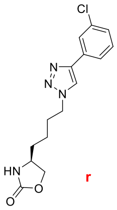

r

Chemical Formula: $C_{15}H_{17}ClN_{4}O_{2}$
Exact Mass: 320.10

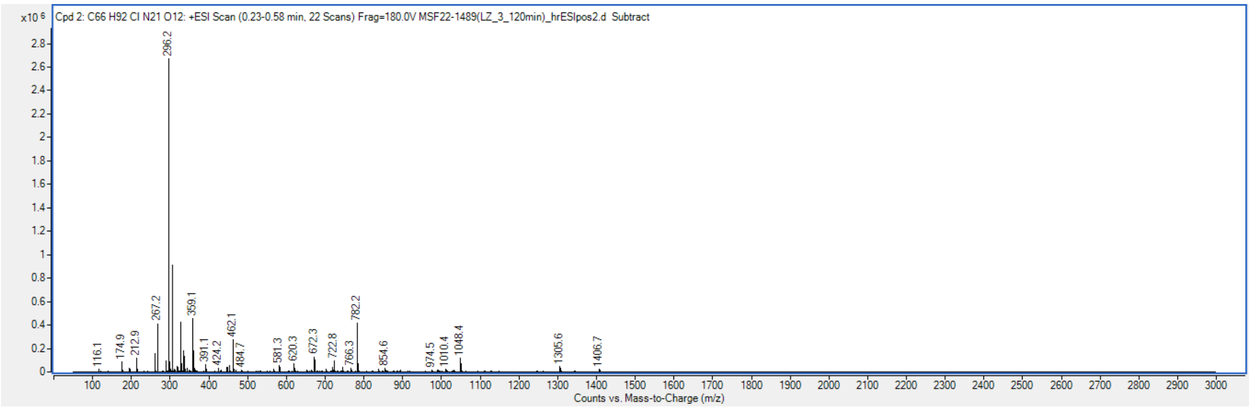

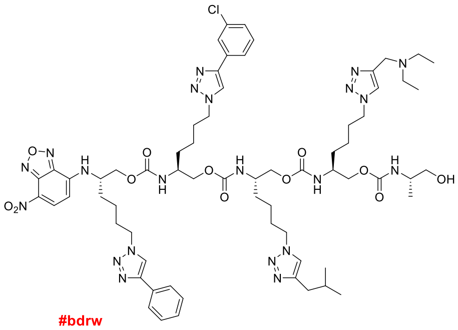

#bdrw

Chemical Formula: $C_{66}H_{92}ClN_{21}O_{12}$
Exact Mass: 1405.69

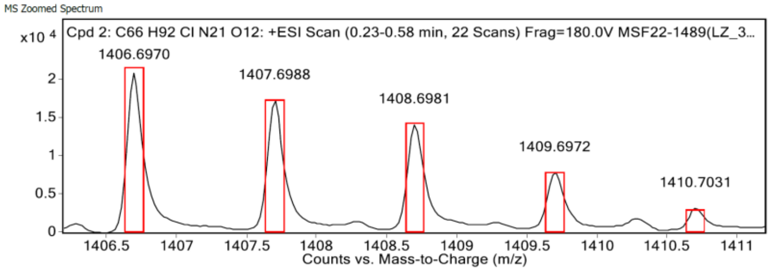

MS Spectrum Peak List

Obs. m/z	Calc. m/z	Charge	Abundance	Formula	Ion Species	Tgt Mass Error (ppm)
296.2078			2679292			
1406.6970	1406.6996	1	20946	C66H92ClN21O12	(M+H)+	1.85
1407.6988	1407.7024	1	17367	C66H92ClN21O12	(M+H)+	2.51
1408.6981	1408.7010	1	14207	C66H92ClN21O12	(M+H)+	2.03
1409.6972	1409.7018	1	7920	C66H92ClN21O12	(M+H)+	3.25
1410.7031	1410.7036	1	3249	C66H92ClN21O12	(M+H)+	0.34

--- End Of Report ---

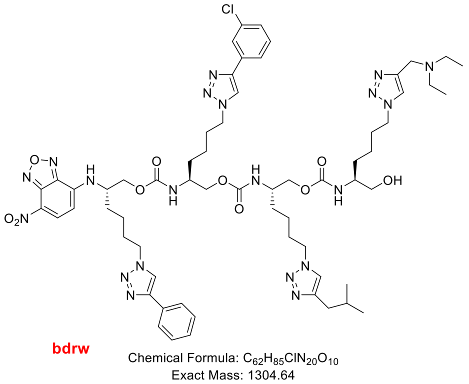

bdrw

Chemical Formula: $C_{62}H_{85}ClN_{20}O_{10}$
Exact Mass: 1304.64

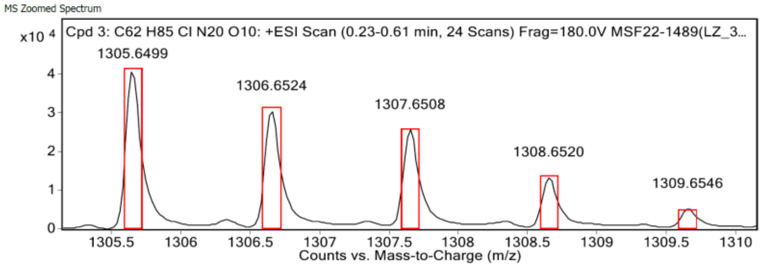

MS Spectrum Peak List

Obs. m/z	Calc. m/z	Charge	Abundance	Formula	Ion Species	Tgt Mass Error (ppm)
296.2079			2522124			
1305.6499	1305.6519	1	41104	C62H85ClN20O10	(M+H)+	1.49
1306.6524	1306.6547	1	30654	C62H85ClN20O10	(M+H)+	1.74
1307.6508	1307.6530	1	25815	C62H85ClN20O10	(M+H)+	1.72
1308.6520	1308.6539	1	13524	C62H85ClN20O10	(M+H)+	1.46
1309.6546	1309.6557	1	5413	C62H85ClN20O10	(M+H)+	0.89
1310.6685	1310.6579	1	1761	C62H85ClN20O10	(M+H)+	-8.07

--- End Of Report ---

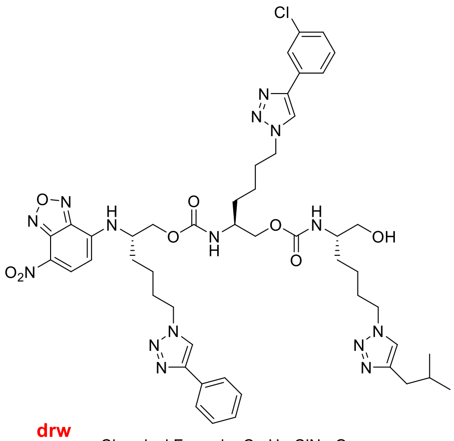

drw

Chemical Formula: $C_{48}H_{60}ClN_{15}O_8$
Exact Mass: 1009.44

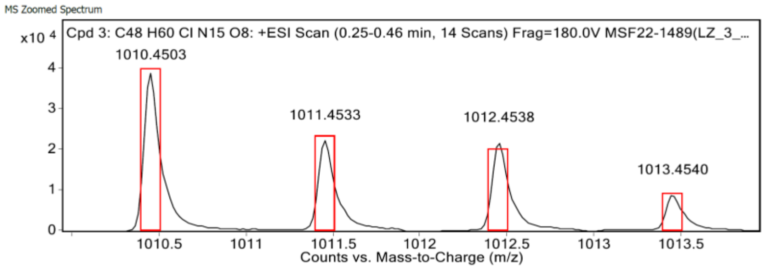

MS Spectrum Peak List

Obs. m/z	Calc. m/z	Charge	Abundance	Formula	Ion Species	Tgt Mass Error (ppm)
296.2081			1284984			
1010.4503	1010.4511	1	38946	C48H60ClN15O8	(M+H)+	0.73
1011.4533	1011.4539	1	22411	C48H60ClN15O8	(M+H)+	0.51
1012.4538	1012.4512	1	21849	C48H60ClN15O8	(M+H)+	-2.62
1013.4540	1013.4524	1	8944	C48H60ClN15O8	(M+H)+	-1.57

--- End Of Report ---

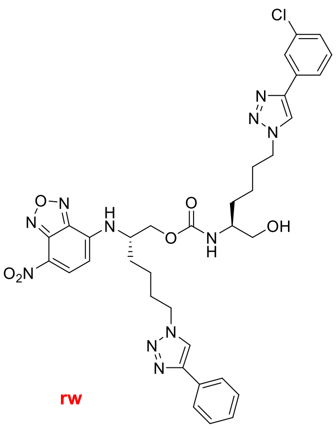

rw

Chemical Formula: $C_{35}H_{38}ClN_{11}O_6$
Exact Mass: 743.27

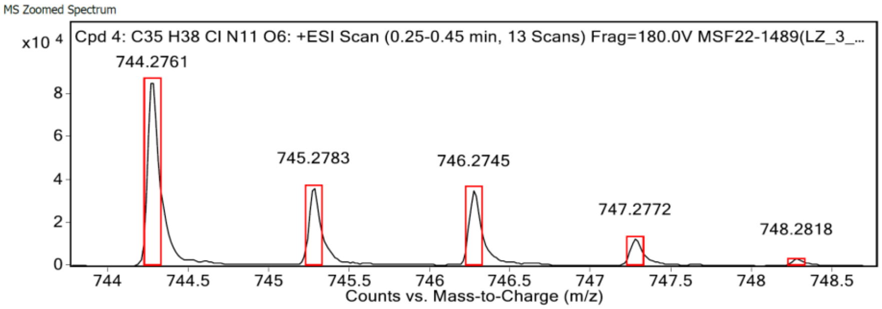

MS Spectrum Peak List

Obs. m/z	Calc. m/z	Charge	Abundance	Formula	Ion Species	Tgt Mass Error (ppm)
296.2081			1166541			
744.2761	744.2768	1	87262	C35H38ClN11O6	(M+H)+	0.91
745.2783	745.2796	1	36653	C35H38ClN11O6	(M+H)+	1.68
746.2745	746.2758	1	35366	C35H38ClN11O6	(M+H)+	1.79
747.2772	747.2775	1	12712	C35H38ClN11O6	(M+H)+	0.43
748.2818	748.2798	1	3413	C35H38ClN11O6	(M+H)+	-2.74
749.2948	749.2821	1	804	C35H38ClN11O6	(M+H)+	-16.85

--- End Of Report ---

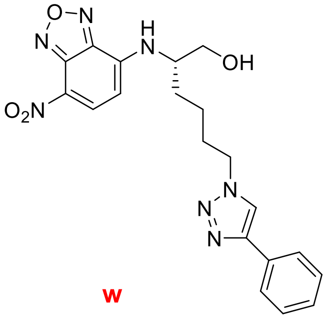

w

Chemical Formula: $C_{20}H_{21}N_7O_4$
Exact Mass: 423.17

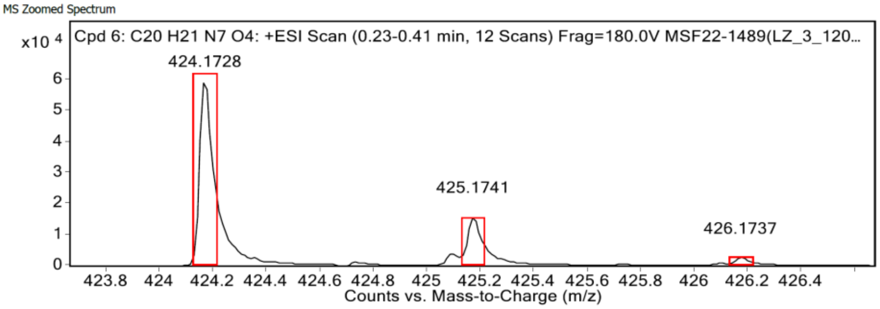

MS Spectrum Peak List

Obs. m/z	Calc. m/z	Charge	Abundance	Formula	Ion Species	Tgt Mass Error (ppm)
296.2077			2624516			
424.1728	424.1728	1	60499	C20H21N7O4	(M+H)+	-0.02
425.1741	425.1755	1	15791	C20H21N7O4	(M+H)+	3.29
426.1737	426.1780	1	2589	C20H21N7O4	(M+H)+	9.88
427.1766	427.1803	1	427	C20H21N7O4	(M+H)+	8.66

--- End Of Report ---

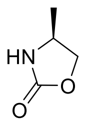

Chemical Formula: $C_4H_7NO_2$
Exact Mass: 101.05

#

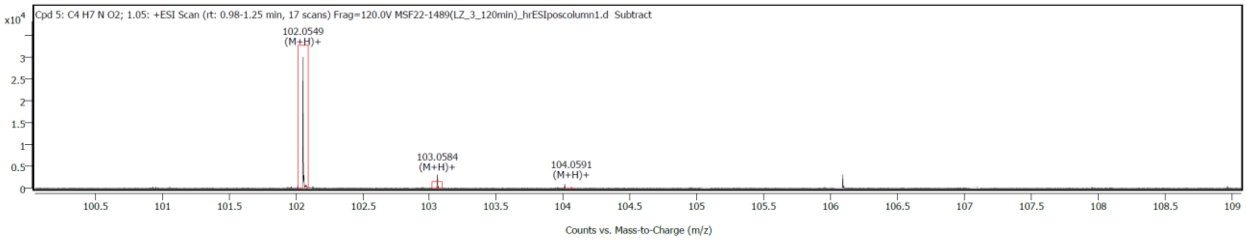

Spectrum Peaks

Obs. m/z	Calc. m/z	Charge	Abund	Formula	Ion Species	Tgt Mass Error (PPM)
102.0549	102.0550	1	30805	C4H7NO2	(M+H)+	-0.41
103.0584	103.0579	1	3115	C4H7NO2	(M+H)+	5.21
104.0591	104.0595	1	236	C4H7NO2	(M+H)+	-3.23

MassHunter Qual 10.0
(End of Report)

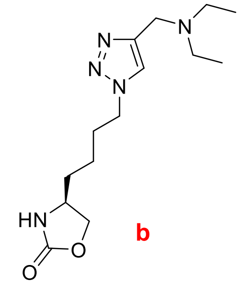

b

Chemical Formula: $C_{14}H_{25}N_5O_2$
Exact Mass: 295.20

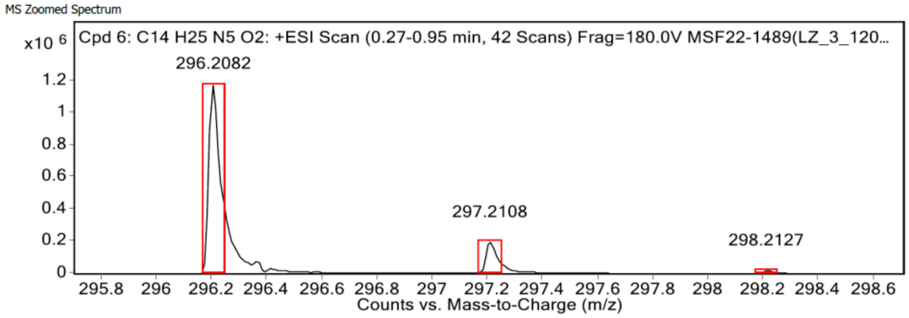

MS Spectrum Peak List

Obs. m/z	Calc. m/z	Charge	Abundance	Formula	Ion Species	Tgt Mass Error (ppm)
296.2082	296.2081	1	1178381	C14H25N5O2	(M+H)+	-0.29
297.2108	297.2108	1	197531	C14H25N5O2	(M+H)+	0.1
298.2127	298.2133	1	20996	C14H25N5O2	(M+H)+	1.84

--- End Of Report ---

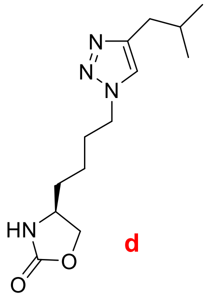

d

Chemical Formula: $C_{13}H_{22}N_4O_2$
Exact Mass: 266.17

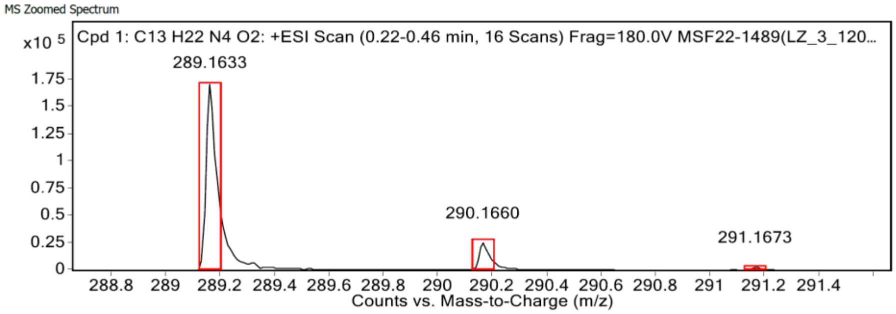

MS Spectrum Peak List

Obs. m/z	Calc. m/z	Charge	Abundance	Formula	Ion Species	Tgt Mass Error (ppm)
289.1633	289.1635	1	171372	C13H22N4O2	(M+Na)+	0.83
290.1660	290.1663	1	25126	C13H22N4O2	(M+Na)+	1
291.1673	291.1687	1	2814	C13H22N4O2	(M+Na)+	4.88
292.1706	292.1711	1	307	C13H22N4O2	(M+Na)+	1.98
296.2081			1067585			

--- End Of Report ---

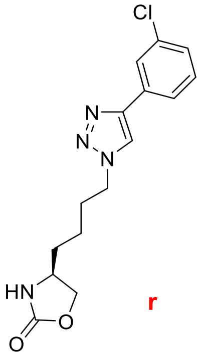

r

Chemical Formula: $C_{15}H_{17}ClN_4O_2$
Exact Mass: 320.10

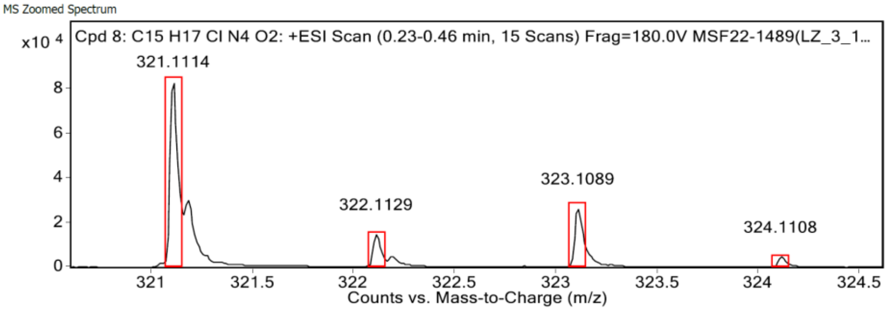

MS Spectrum Peak List

Obs. m/z	Calc. m/z	Charge	Abundance	Formula	Ion Species	Tgt Mass Error (ppm)
296.2081			1101688			
321.1114	321.1113	1	83930	C15H17ClN4O2	(M+H)+	-0.3
322.1129	322.1142	1	14904	C15H17ClN4O2	(M+H)+	3.77
323.1089	323.1088	1	26472	C15H17ClN4O2	(M+H)+	-0.44
324.1108	324.1114	1	4739	C15H17ClN4O2	(M+H)+	1.92
325.1073	325.1139	1	3819	C15H17ClN4O2	(M+H)+	20.22
326.1111	326.1163	1	815	C15H17ClN4O2	(M+H)+	15.7

--- End Of Report ---

Oligomer 4: #7fd2

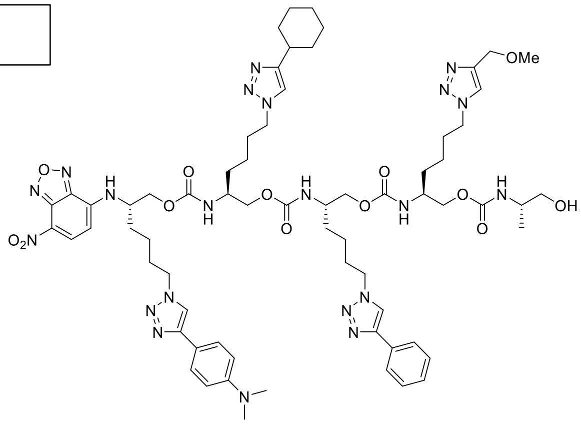

Exact Mass: 1399.73

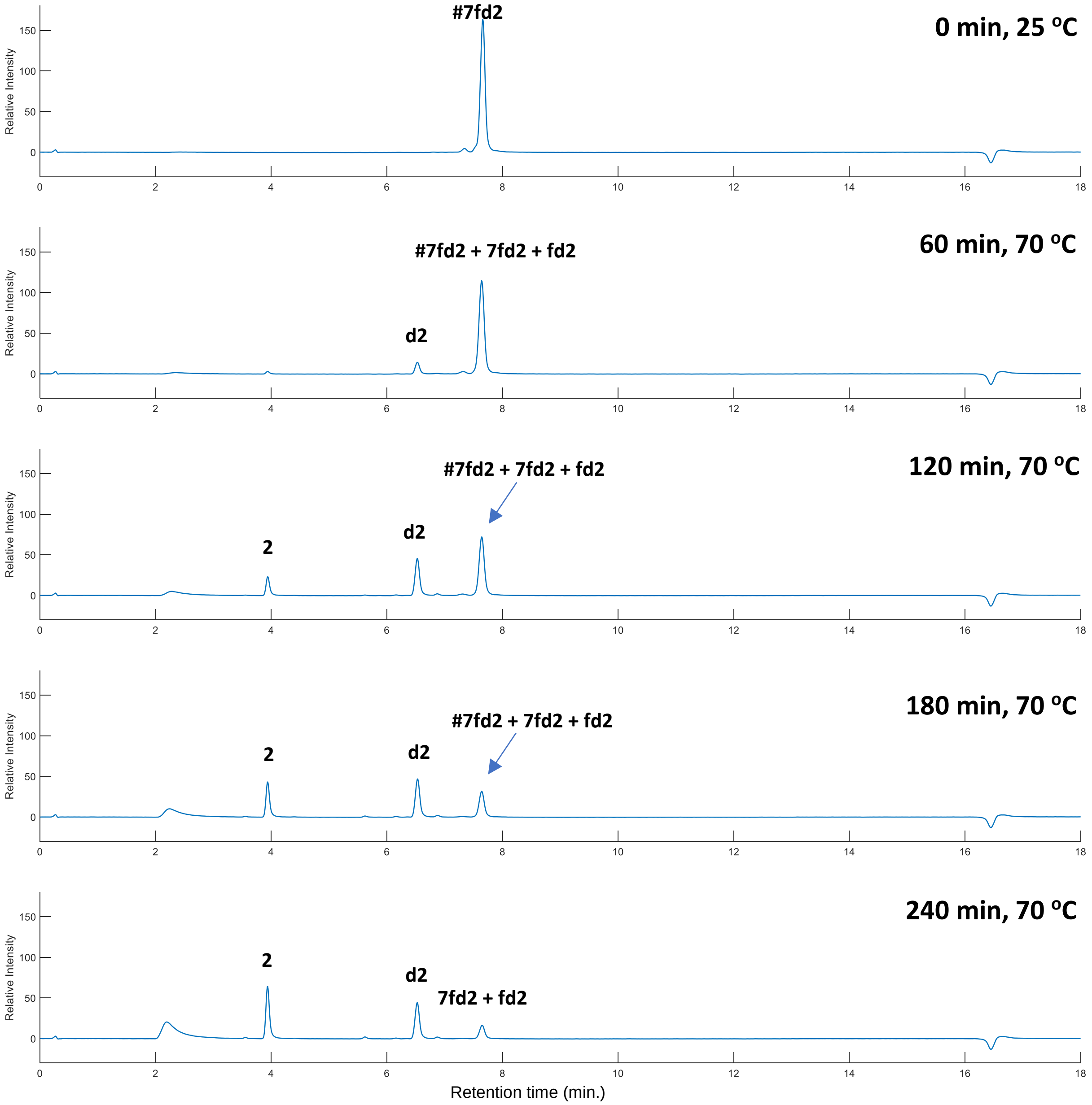

After 120 minutes of sequencing, all the following molecules are observed in the solution and analyzed via High-Res MS.

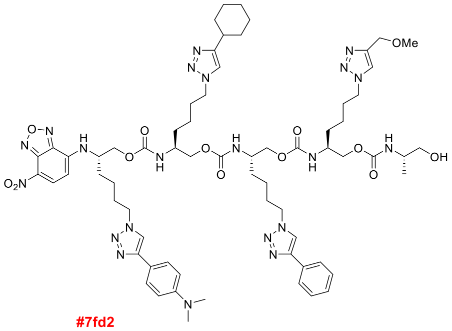

#7fd2

Chemical Formula: $C_{67}H_{93}N_{21}O_{13}$
Exact Mass: 1399.73

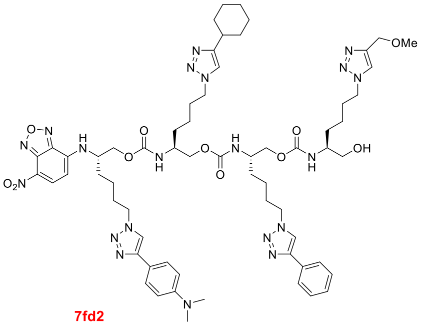

7fd2

Chemical Formula: $C_{63}H_{86}N_{20}O_{11}$
Exact Mass: 1298.68

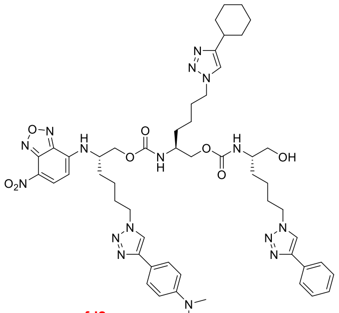

fd2

Chemical Formula: $C_{52}H_{68}N_{16}O_{8}$
Exact Mass: 1044.54

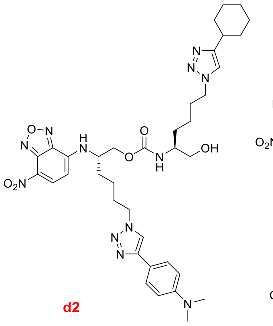

d2

Chemical Formula: $C_{37}H_{50}N_{12}O_{6}$
Exact Mass: 758.40

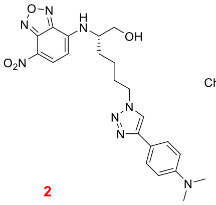

2

Chemical Formula: $C_{22}H_{26}N_{8}O_{4}$
Exact Mass: 466.21

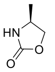

Chemical Formula: $C_{4}H_{7}NO_{2}$
Exact Mass: 101.05

#

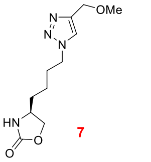

7

Chemical Formula: $C_{11}H_{18}N_{4}O_{3}$
Exact Mass: 254.14

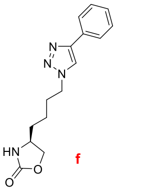

f

Chemical Formula: $C_{15}H_{18}N_{4}O_{2}$
Exact Mass: 286.14

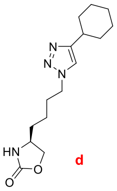

d

Chemical Formula: $C_{15}H_{24}N_{4}O_{2}$
Exact Mass: 292.19

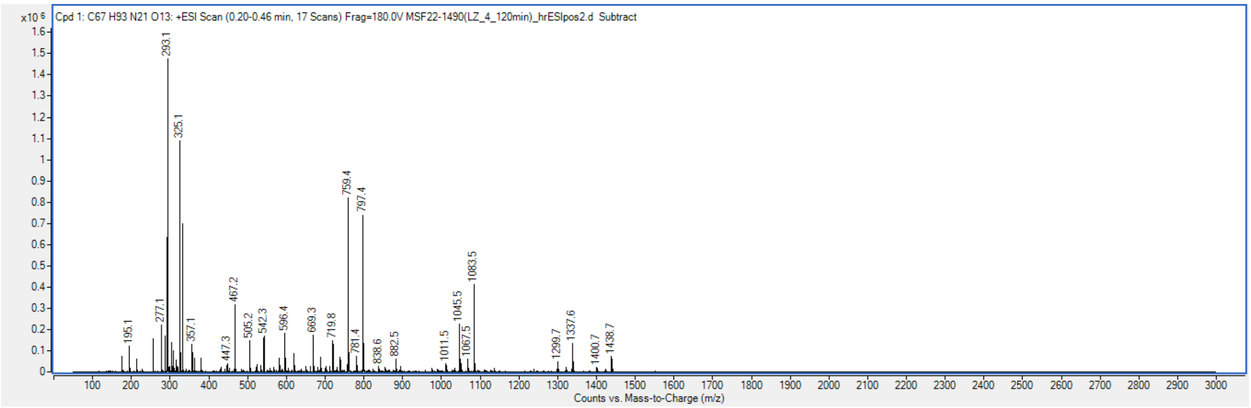

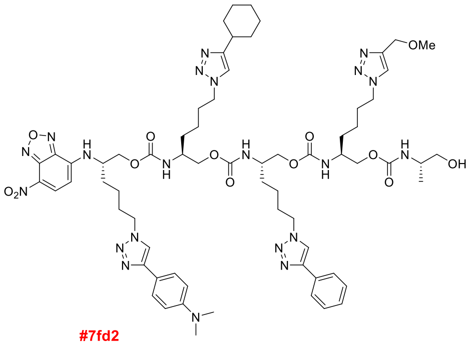

#7fd2

Chemical Formula: $C_{67}H_{93}N_{21}O_{13}$
Exact Mass: 1399.73

MS Zoomed Spectrum

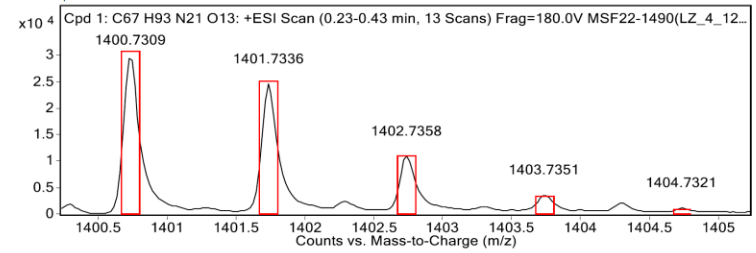

MS Spectrum Peak List

Obs. m/z	Calc. m/z	Charge	Abundance	Formula	Ion Species	Tgt Mass Error (ppm)
293.1010			1779023			
1400.7309	1400.7334	1	30107	C67H93N21O13	(M+H)+	1.8
1401.7336	1401.7363	1	24759	C67H93N21O13	(M+H)+	1.87
1402.7358	1402.7389	1	11096	C67H93N21O13	(M+H)+	2.24
1403.7351	1403.7416	1	3793	C67H93N21O13	(M+H)+	4.61
1404.7321	1404.7441	1	1357	C67H93N21O13	(M+H)+	8.58

--- End Of Report ---

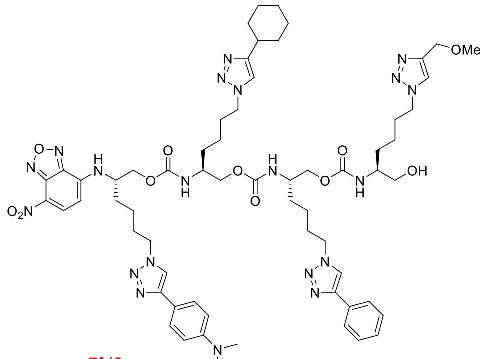

7fd2

Chemical Formula: $C_{63}H_{86}N_{20}O_{11}$
Exact Mass: 1298.68

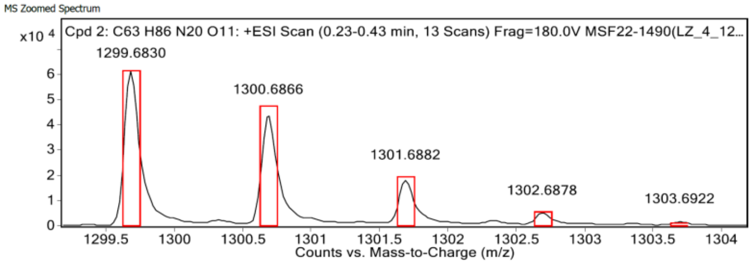

MS Spectrum Peak List

Obs. m/z	Calc. m/z	Charge	Abundance	Formula	Ion Species	Tgt Mass Error (ppm)
293.1010			1779991			
1299.6830	1299.6858	1	61539	C63H86N20O11	(M+H)+	2.1
1300.6866	1300.6886	1	44497	C63H86N20O11	(M+H)+	1.53
1301.6882	1301.6913	1	18568	C63H86N20O11	(M+H)+	2.34
1302.6878	1302.6939	1	4546	C63H86N20O11	(M+H)+	4.67
1303.6922	1303.6964	1	1229	C63H86N20O11	(M+H)+	3.26
1304.6768	1304.6990	1	248	C63H86N20O11	(M+H)+	17

--- End Of Report ---

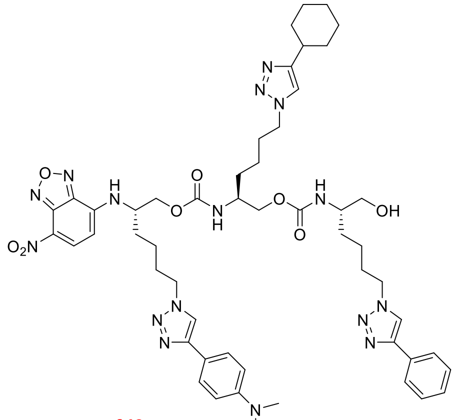

fd2

Chemical Formula: $C_{52}H_{68}N_{16}O_8$
Exact Mass: 1044.54

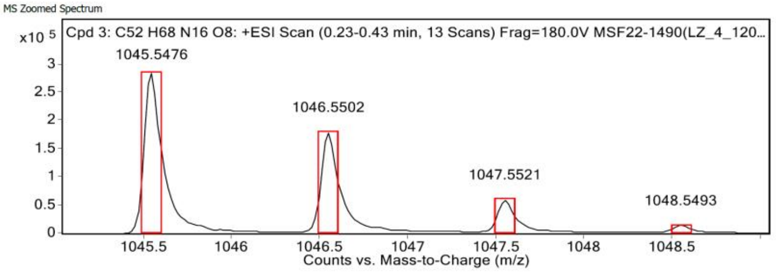

MS Spectrum Peak List

Obs. m/z	Calc. m/z	Charge	Abundance	Formula	Ion Species	Tgt Mass Error (ppm)
293.1010			1777930			
1045.5476	1045.5479	1	285185	C52H68N16O8	(M+H)+	0.22
1046.5502	1046.5507	1	178740	C52H68N16O8	(M+H)+	0.52
1047.5521	1047.5534	1	58956	C52H68N16O8	(M+H)+	1.27
1048.5493	1048.5560	1	13187	C52H68N16O8	(M+H)+	6.43

--- End Of Report ---

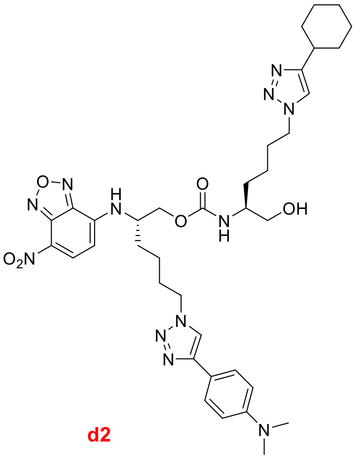

d2

Chemical Formula: $C_{37}H_{50}N_{12}O_6$
Exact Mass: 758.40

MS Zoomed Spectrum

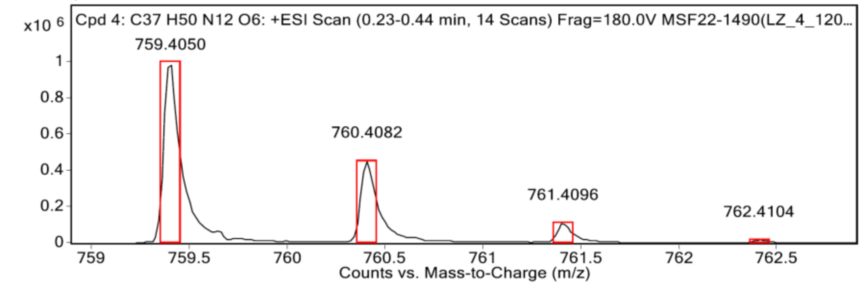

MS Spectrum Peak List

Obs. m/z	Calc. m/z	Charge	Abundance	Formula	Ion Species	Tgt Mass Error (ppm)
293.1010			1773373			
759.4050	759.4049	1	1002280	C37H50N12O6	(M+H)+	-0.13
760.4082	760.4077	1	455872	C37H50N12O6	(M+H)+	-0.73
761.4096	761.4103	1	111402	C37H50N12O6	(M+H)+	0.92
762.4104	762.4129	1	17271	C37H50N12O6	(M+H)+	3.16
763.4086	763.4153	1	2466	C37H50N12O6	(M+H)+	8.86

--- End Of Report ---

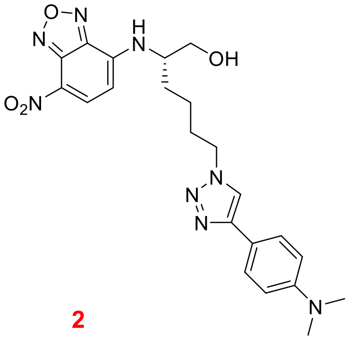

2

Chemical Formula: $C_{22}H_{26}N_8O_4$
Exact Mass: 466.21

MS Zoomed Spectrum

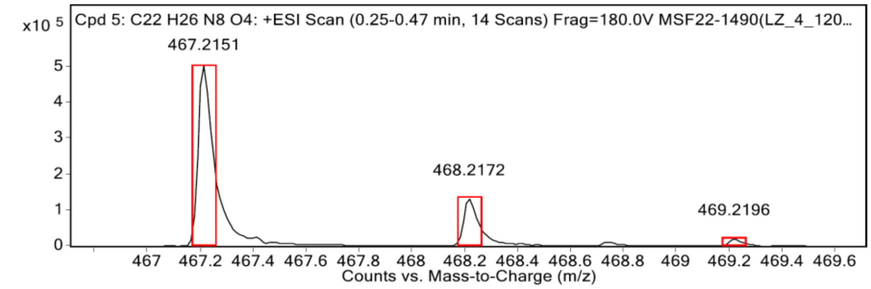

MS Spectrum Peak List

Obs. m/z	Calc. m/z	Charge	Abundance	Formula	Ion Species	Tgt Mass Error (ppm)
467.2151	467.2150	1	503378	C22H26N8O4	(M+H)+	-0.35
468.2172	468.2177	1	131744	C22H26N8O4	(M+H)+	1.04
469.2196	469.2202	1	20692	C22H26N8O4	(M+H)+	1.17
470.2523	470.2226	1	2501	C22H26N8O4	(M+H)+	-63.31
471.2134	471.2249	1	569	C22H26N8O4	(M+H)+	24.42
759.4044			1429557			

--- End Of Report ---

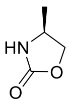

Chemical Formula: $C_4H_7NO_2$
Exact Mass: 101.05

#

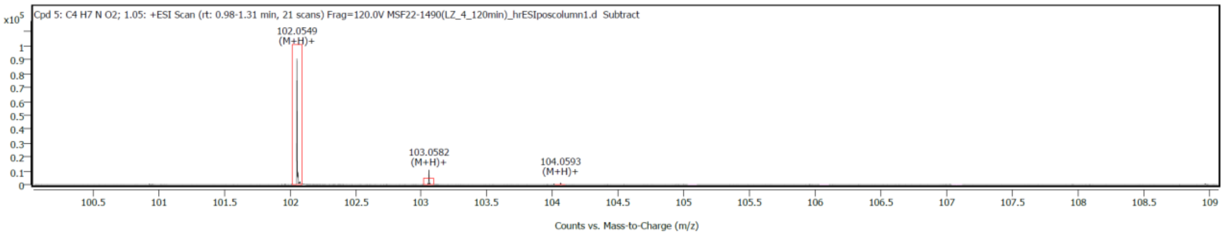

Spectrum Peaks

Obs. m/z	Calc. m/z	Charge	Abund	Formula	Ion Species	Tgt Mass Error (PPM)
102.0549	102.0550	1	94020	C4H7NO2	(M+H)+	-0.58
103.0582	103.0579	1	10733	C4H7NO2	(M+H)+	2.96
104.0593	104.0595	1	1258	C4H7NO2	(M+H)+	-1.74

MassHunter Qual 10.0
(End of Report)

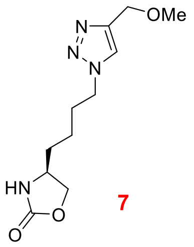

7

Chemical Formula: $C_{11}H_{18}N_4O_3$
Exact Mass: 254.14

MS Zoomed Spectrum

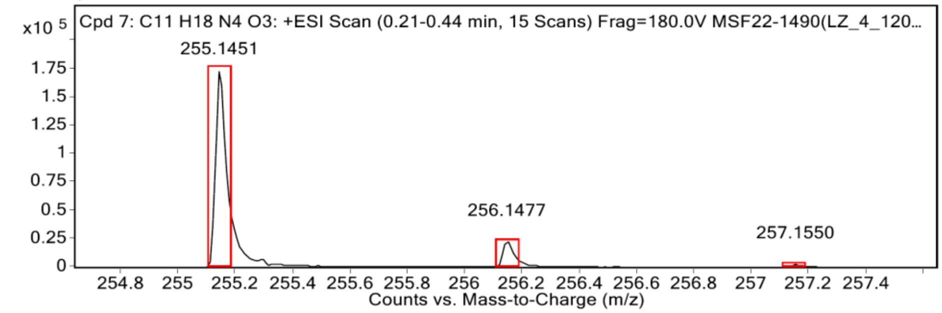

MS Spectrum Peak List

Obs. m/z	Calc. m/z	Charge	Abundance	Formula	Ion Species	Tgt Mass Error (ppm)
255.1451	255.1452	1	176362	C11H18N4O3	(M+H)+	0.35
256.1477	256.1479	1	22645	C11H18N4O3	(M+H)+	0.96
257.1550	257.1501	1	2804	C11H18N4O3	(M+H)+	-19.13
258.1715	258.1524	1	421	C11H18N4O3	(M+H)+	-73.93
293.1010			1625527			

--- End Of Report ---

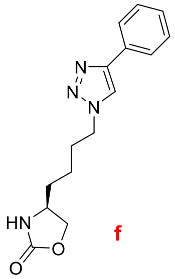

f

Chemical Formula: $C_{15}H_{18}N_4O_2$
Exact Mass: 286.14

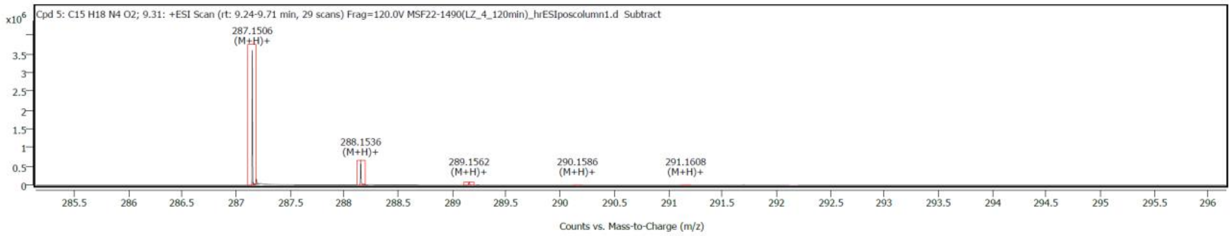

Spectrum Peaks

Obs. m/z	Calc. m/z	Charge	Abund	Formula	Ion Species	Tgt Mass Error (PPM)
287.1506	287.1503	1	3714401	C15H18N4O2	(M+H)+	1.17
288.1536	288.1531	1	685425	C15H18N4O2	(M+H)+	1.75
289.1562	289.1557	1	75108	C15H18N4O2	(M+H)+	1.72
290.1586	290.1581	1	5989	C15H18N4O2	(M+H)+	1.58
291.1608	291.1606	1	445	C15H18N4O2	(M+H)+	0.62

MassHunter Qual 10.0
(End of Report)

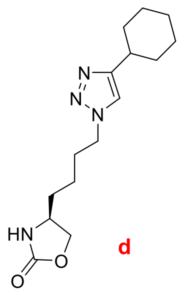

d

Chemical Formula: $C_{15}H_{24}N_4O_2$

Exact Mass: 292.19

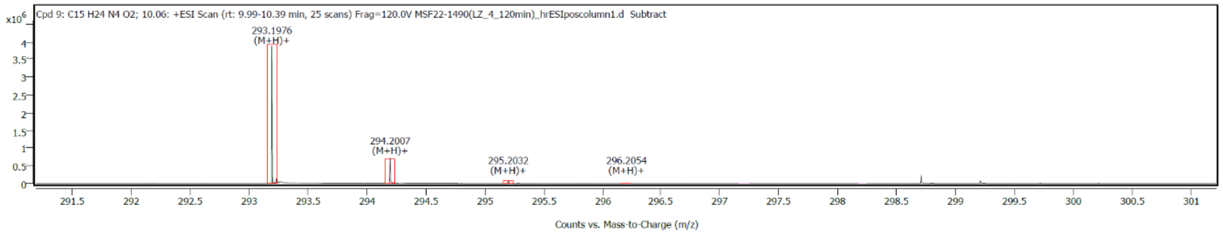

Spectrum Peaks

Obs. m/z	Calc. m/z	Charge	Abund	Formula	Ion Species	Tgt Mass Error (PPM)
293.1976	293.1972	1	3885574	C15H24N4O2	(M+H)+	1.37
294.2007	294.2001	1	729886	C15H24N4O2	(M+H)+	1.97
295.2032	295.2026	1	80608	C15H24N4O2	(M+H)+	1.93
296.2054	296.2051	1	6756	C15H24N4O2	(M+H)+	1.04

MassHunter Qual 10.0
(End of Report)

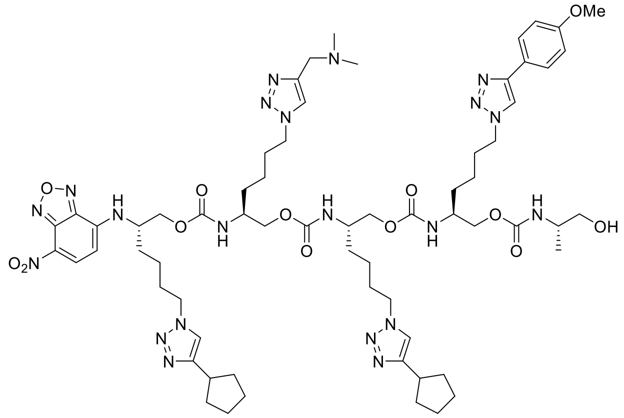

Exact Mass: 1377.74

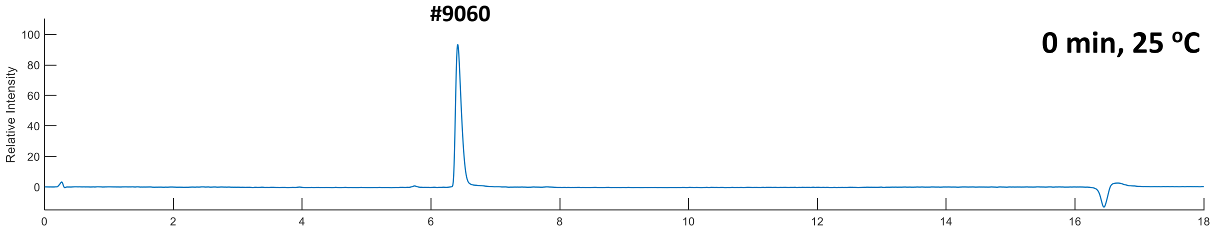

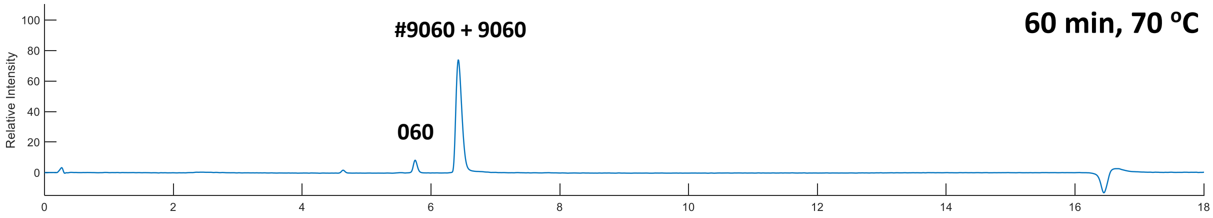

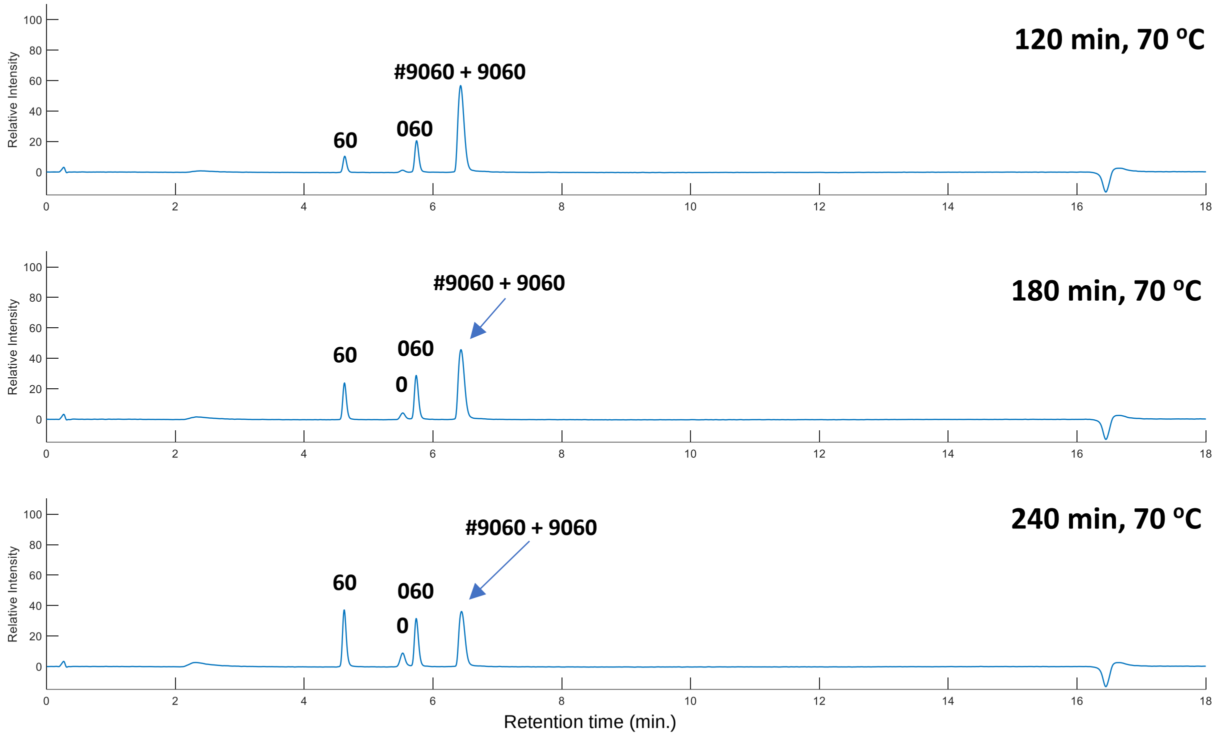

After 120 minutes of sequencing, all the following molecules are observed in the solution and analyzed via High-Res MS.

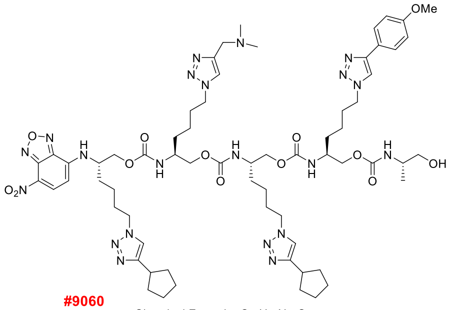

#9060

Chemical Formula: $C_{65}H_{95}N_{21}O_{13}$
Exact Mass: 1377.74

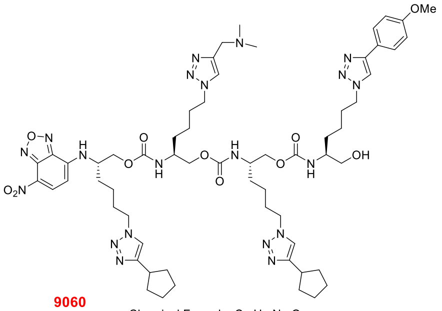

9060

Chemical Formula: $C_{61}H_{88}N_{20}O_{11}$
Exact Mass: 1276.69

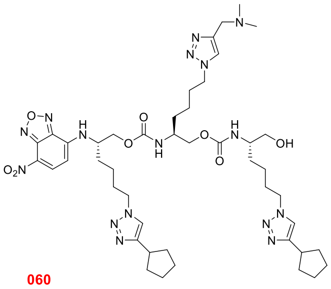

060

Chemical Formula: $C_{45}H_{68}N_{16}O_8$
Exact Mass: 960.54

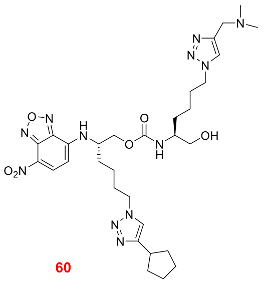

60

Chemical Formula: $C_{31}H_{46}N_{12}O_6$
Exact Mass: 682.37

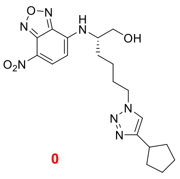

0

Chemical Formula: $C_{19}H_{25}N_7O_4$
Exact Mass: 415.20

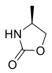

Chemical Formula: $C_4H_7NO_2$
Exact Mass: 101.05

#

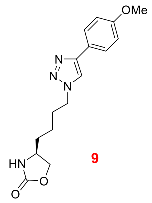

9

Chemical Formula: $C_{16}H_{20}N_4O_3$
Exact Mass: 316.15

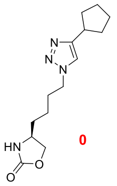

0

Chemical Formula: $C_{14}H_{22}N_4O_2$
Exact Mass: 278.17

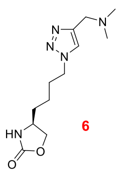

6

Chemical Formula: $C_{12}H_{21}N_5O_2$
Exact Mass: 267.17

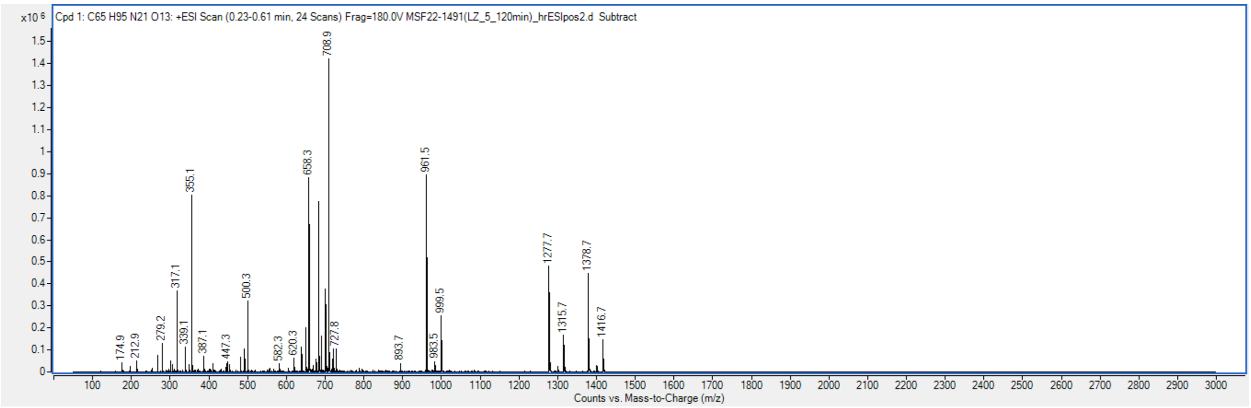

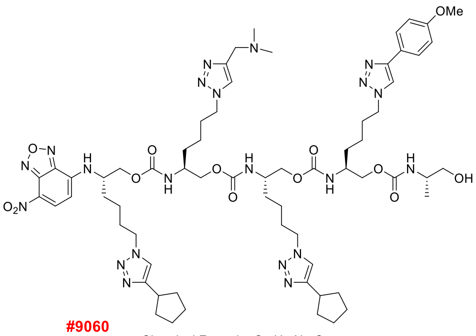

#9060

Chemical Formula: $C_{65}H_{95}N_{21}O_{13}$
Exact Mass: 1377.74

MS Zoomed Spectrum

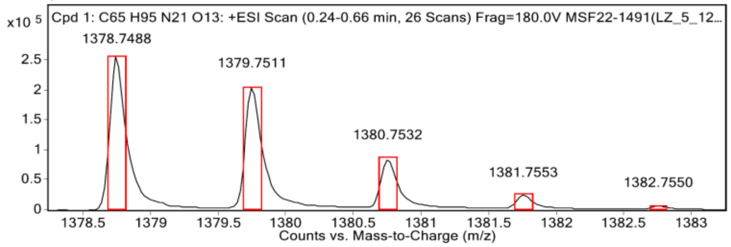

MS Spectrum Peak List

Obs. m/z	Calc. m/z	Charge	Abundance	Formula	Ion Species	Tgt Mass Error (ppm)
708.8576			786927			
1378.7488	1378.7491	1	257452	C65H95N21O13	(M+H)+	0.24
1379.7511	1379.7519	1	205424	C65H95N21O13	(M+H)+	0.56
1380.7532	1380.7546	1	85241	C65H95N21O13	(M+H)+	0.99
1381.7553	1381.7572	1	22253	C65H95N21O13	(M+H)+	1.38
1382.7550	1382.7597	1	5388	C65H95N21O13	(M+H)+	3.43
1383.7472	1383.7622	1	1195	C65H95N21O13	(M+H)+	10.84
1384.7199	1384.7647	1	391	C65H95N21O13	(M+H)+	32.4

--- End Of Report ---

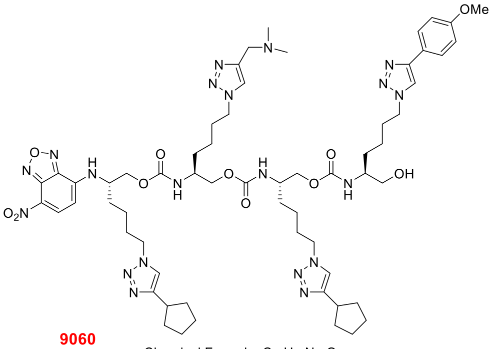

9060

Chemical Formula: $C_{61}H_{88}N_{20}O_{11}$
Exact Mass: 1276.69

MS Zoomed Spectrum

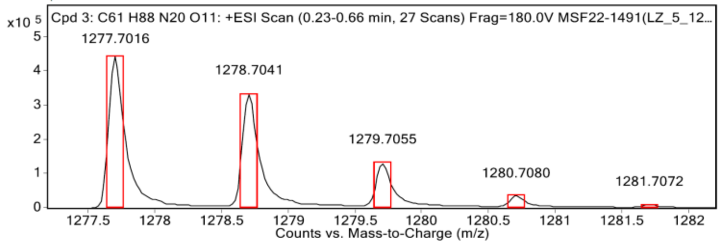

MS Spectrum Peak List

Obs. m/z	Calc. m/z	Charge	Abundance	Formula	Ion Species	Tgt Mass Error (ppm)
708.8569			1373255			
1277.7016	1277.7014	1	444067	C61H88N20O11	(M+H)+	-0.1
1278.7041	1278.7042	1	332958	C61H88N20O11	(M+H)+	0.11
1279.7055	1279.7069	1	130646	C61H88N20O11	(M+H)+	1.04
1280.7080	1280.7095	1	31737	C61H88N20O11	(M+H)+	1.17
1281.7072	1281.7120	1	7262	C61H88N20O11	(M+H)+	3.76
1282.7086	1282.7145	1	1469	C61H88N20O11	(M+H)+	4.67

--- End Of Report ---

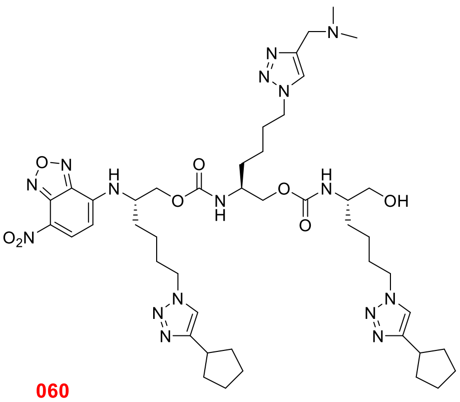

060

Chemical Formula: $C_{45}H_{68}N_{16}O_8$
Exact Mass: 960.54

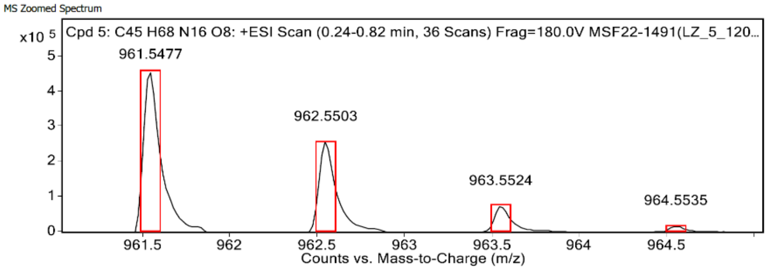

MS Spectrum Peak List

Obs. m/z	Calc. m/z	Charge	Abundance	Formula	Ion Species	Tgt Mass Error (ppm)
708.8576			721214			
961.5477	961.5479	1	455760	C45H68N16O8	(M+H)+	0.18
962.5503	962.5506	1	257267	C45H68N16O8	(M+H)+	0.29
963.5524	963.5532	1	74346	C45H68N16O8	(M+H)+	0.84
964.5535	964.5558	1	17695	C45H68N16O8	(M+H)+	2.33
965.5507	965.5582	1	3226	C45H68N16O8	(M+H)+	7.82

--- End Of Report ---

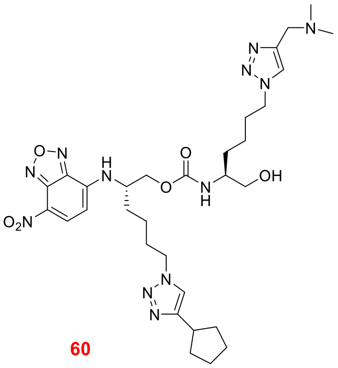

60

Chemical Formula: $C_{31}H_{46}N_{12}O_6$
Exact Mass: 682.37

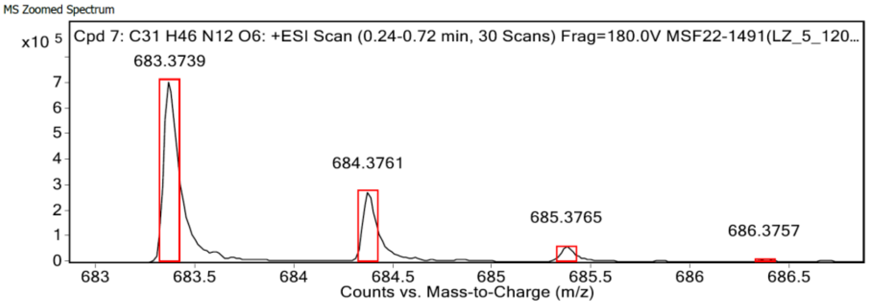

MS Spectrum Peak List

Obs. m/z	Calc. m/z	Charge	Abundance	Formula	Ion Species	Tgt Mass Error (ppm)
683.3739	683.3736	1	711672	C31H46N12O6	(M+H)+	-0.47
684.3761	684.3763	1	274587	C31H46N12O6	(M+H)+	0.3
685.3765	685.3788	1	62449	C31H46N12O6	(M+H)+	3.41
686.3757	686.3812	1	9978	C31H46N12O6	(M+H)+	8.04
687.3494	687.3836	1	2431	C31H46N12O6	(M+H)+	49.82
708.8570			1317642			

--- End Of Report ---

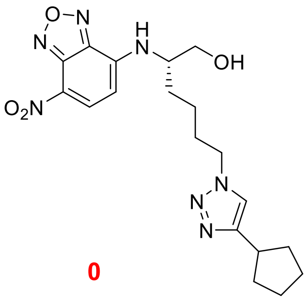

0

Chemical Formula: $C_{19}H_{25}N_7O_4$
Exact Mass: 415.20

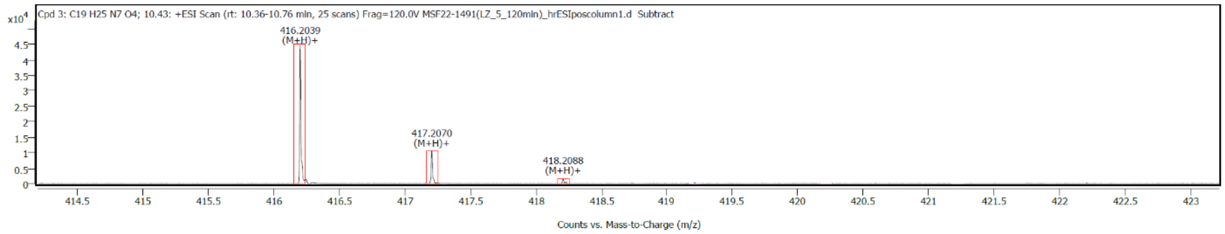

Spectrum Peaks

Obs. m/z	Calc. m/z	Charge	Abund	Formula	Ion Species	Tgt Mass Error (PPM)
416.2039	416.2041	1	44601	C19H25N7O4	(M+H)+	-0.54
417.2070	417.2068	1	10731	C19H25N7O4	(M+H)+	0.46
418.2088	418.2092	1	1547	C19H25N7O4	(M+H)+	-0.85
481.2778			61066			

MassHunter Qual 10.0
(End of Report)

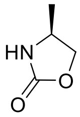

Chemical Formula: $C_4H_7NO_2$
Exact Mass: 101.05

#

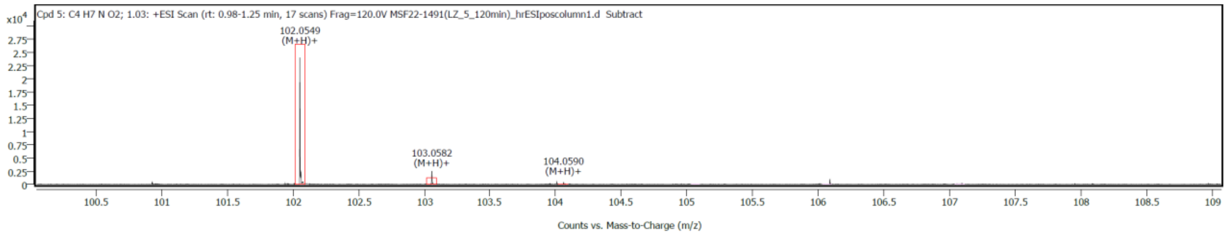

Spectrum Peaks

Obs. m/z	Calc. m/z	Charge	Abund	Formula	Ion Species	Tgt Mass Error (PPM)
102.0549	102.0550	1	24958	C4H7NO2	(M+H)+	-0.36
103.0582	103.0579	1	2557	C4H7NO2	(M+H)+	2.59
104.0590	104.0595	1	363	C4H7NO2	(M+H)+	-5.09
922.0036			37361			

MassHunter Qual 10.0
(End of Report)

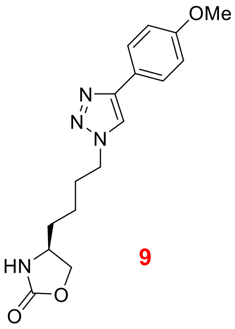

9

Chemical Formula: $C_{16}H_{20}N_4O_3$
Exact Mass: 316.15

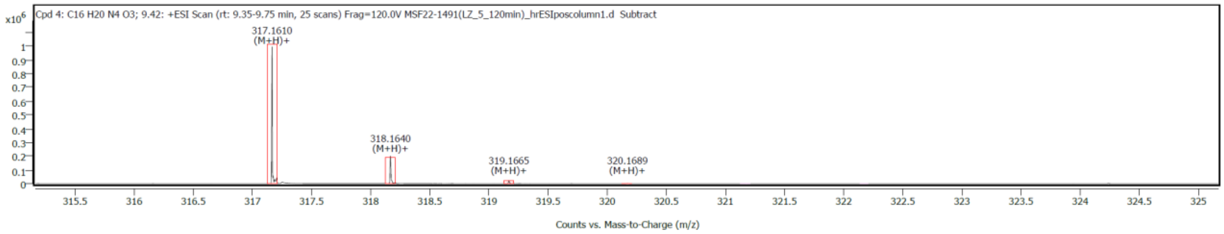

Spectrum Peaks

Obs. m/z	Calc. m/z	Charge	Abund	Formula	Ion Species	Tgt Mass Error (PPM)
317.1610	317.1608	1	1001021	C16H20N4O3	(M+H)+	0.63
318.1640	318.1637	1	202360	C16H20N4O3	(M+H)+	0.82
319.1665	319.1662	1	24087	C16H20N4O3	(M+H)+	0.79
320.1689	320.1687	1	2224	C16H20N4O3	(M+H)+	0.71

MassHunter Qual 10.0
(End of Report)

MS Zoomed Spectrum

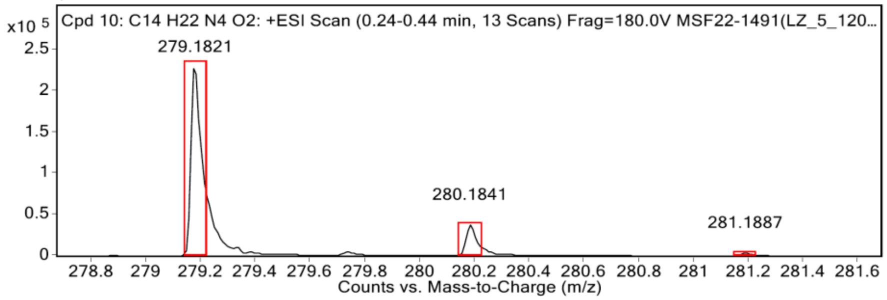

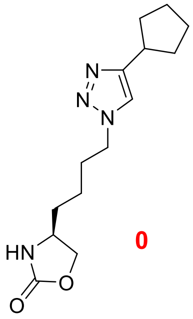

0

Chemical Formula: $C_{14}H_{22}N_4O_2$
Exact Mass: 278.17

MS Spectrum Peak List

Obs. m/z	Calc. m/z	Charge	Abundance	Formula	Ion Species	Tgt Mass Error (ppm)
279.1821	279.1816	1	234825	C14H22N4O2	(M+H)+	-2.05
280.1841	280.1844	1	38205	C14H22N4O2	(M+H)+	1.15
281.1887	281.1869	1	4162	C14H22N4O2	(M+H)+	-6.49
708.8570			1426713			

--- End Of Report ---

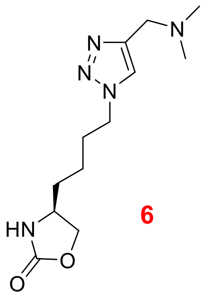

6

Chemical Formula: $C_{12}H_{21}N_5O_2$
Exact Mass: 267.17

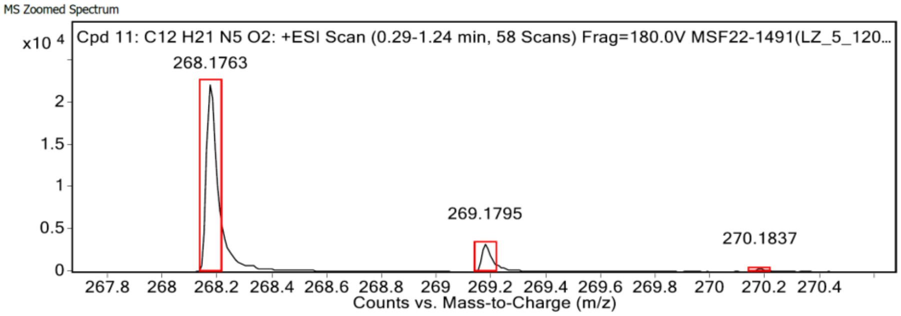

MS Spectrum Peak List

Obs. m/z	Calc. m/z	Charge	Abundance	Formula	Ion Species	Tgt Mass Error (ppm)
268.1763	268.1768	1	22578	C12H21N5O2	(M+H)+	1.89
269.1795	269.1794	1	3329	C12H21N5O2	(M+H)+	-0.05
270.1837	270.1818	1	398	C12H21N5O2	(M+H)+	-7.03
708.8576			525030			

--- End Of Report ---

Oligomer 6_1: *76f8

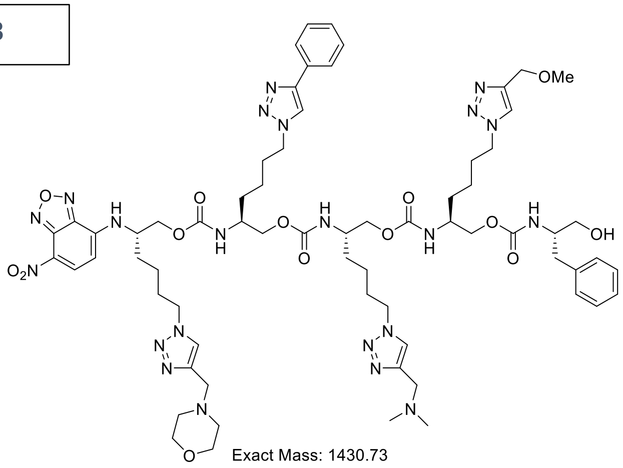

Exact Mass: 1430.73

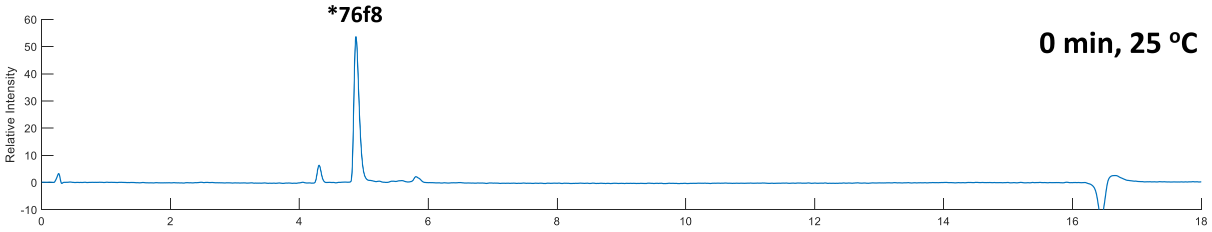

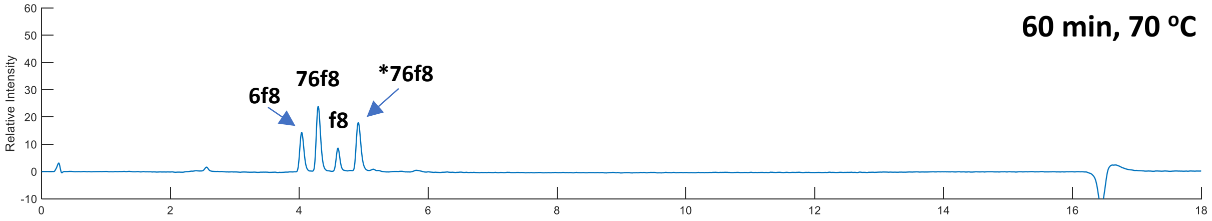

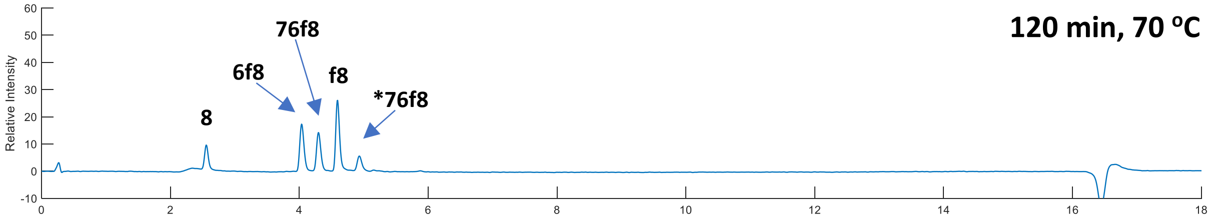

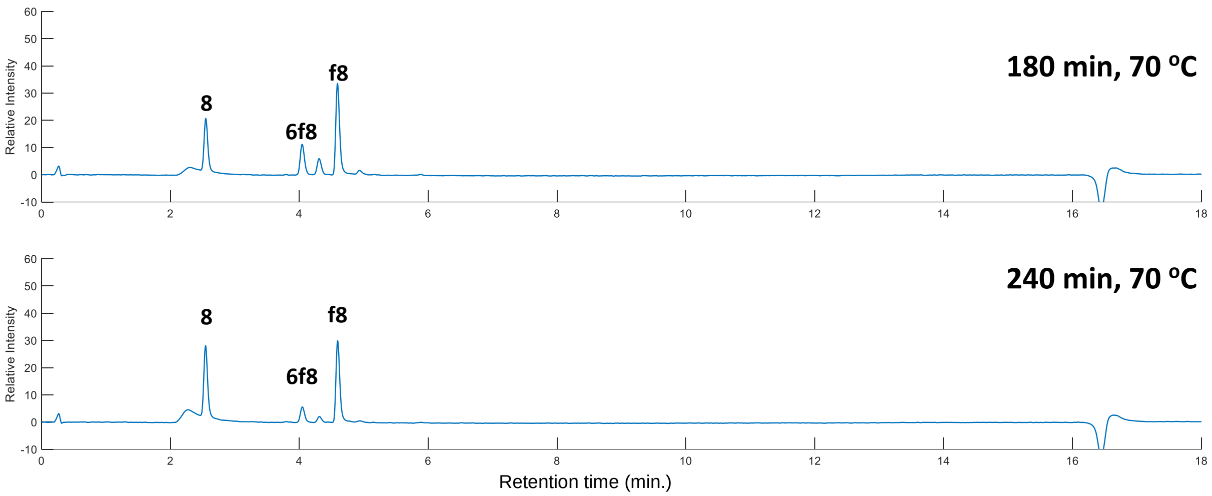

After 120 minutes of sequencing, all the following molecules are observed in the solution and analyzed via High-Res MS.

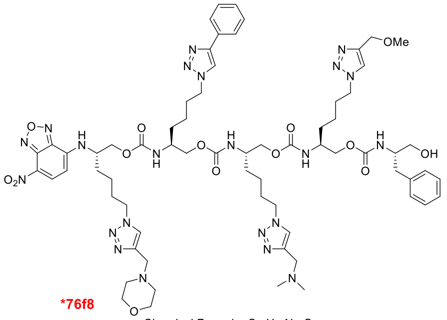

*76f8

Chemical Formula: $C_{67}H_{94}N_{22}O_{14}$
Exact Mass: 1430.73

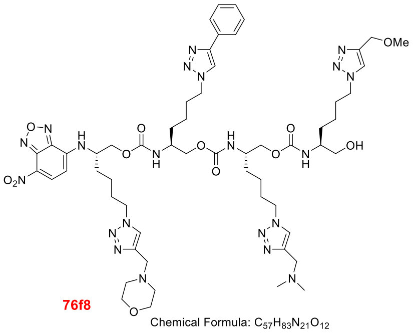

76f8

Chemical Formula: $C_{57}H_{83}N_{21}O_{12}$
Exact Mass: 1253.65

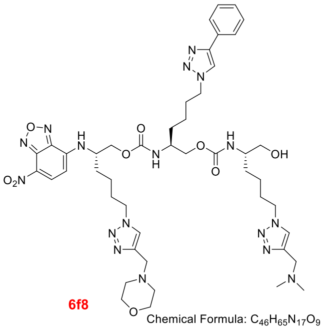

6f8

Chemical Formula: $C_{46}H_{65}N_{17}O_{9}$
Exact Mass: 999.52

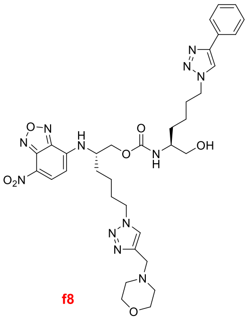

f8

Chemical Formula: $C_{34}H_{44}N_{12}O_{7}$
Exact Mass: 732.35

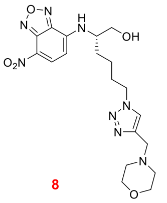

8

Chemical Formula: $C_{19}H_{26}N_{8}O_{5}$
Exact Mass: 446.20

*

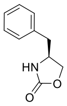

Chemical Formula: $C_{10}H_{11}NO_{2}$
Exact Mass: 177.08

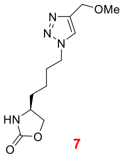

7

Chemical Formula: $C_{11}H_{18}N_{4}O_{3}$
Exact Mass: 254.14

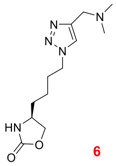

6

Chemical Formula: $C_{12}H_{21}N_{5}O_{2}$
Exact Mass: 267.17

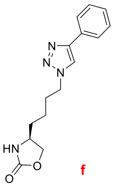

f

Chemical Formula: $C_{15}H_{18}N_{4}O_{2}$
Exact Mass: 286.14

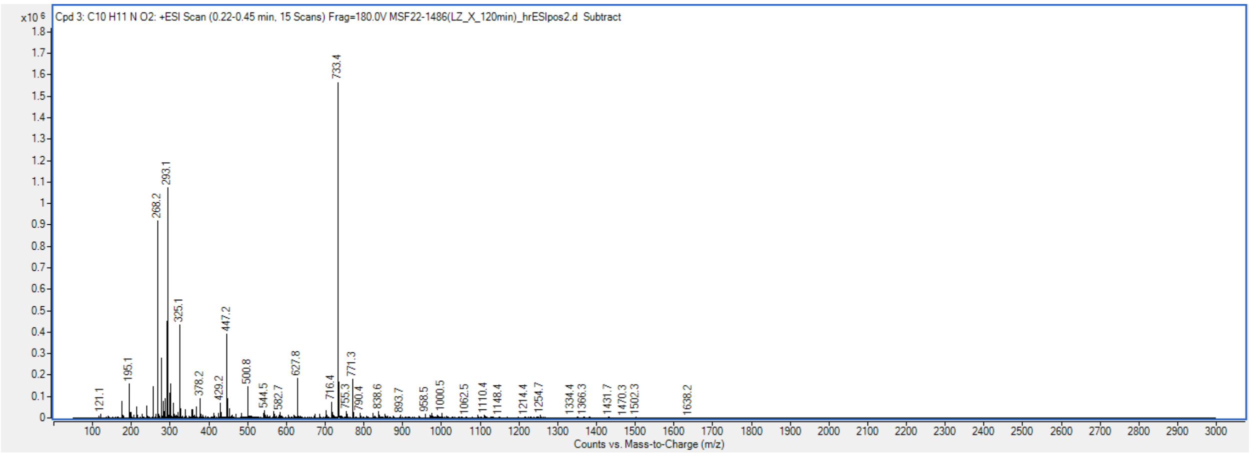

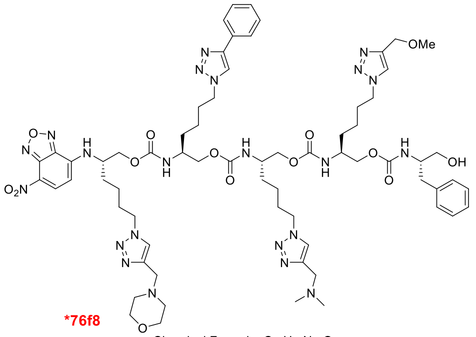

***76f8**

Chemical Formula: $C_{67}H_{94}N_{22}O_{14}$
Exact Mass: 1430.73

MS Zoomed Spectrum

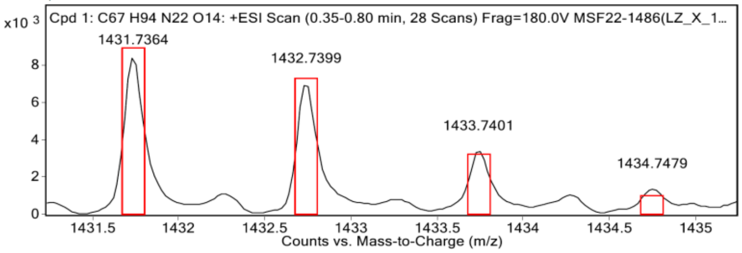

MS Spectrum Peak List

Obs. m/z	Calc. m/z	Charge	Abundance	Formula	Ion Species	Tgt Mass Error (ppm)
268.1768			877491			
1431.7364	1431.7393	1	8465	C67H94N22O14	(M+H)+	2.03
1432.7399	1432.7420	1	7071	C67H94N22O14	(M+H)+	1.5
1433.7401	1433.7447	1	3455	C67H94N22O14	(M+H)+	3.19
1434.7479	1434.7473	1	1425	C67H94N22O14	(M+H)+	-0.4

--- End Of Report ---

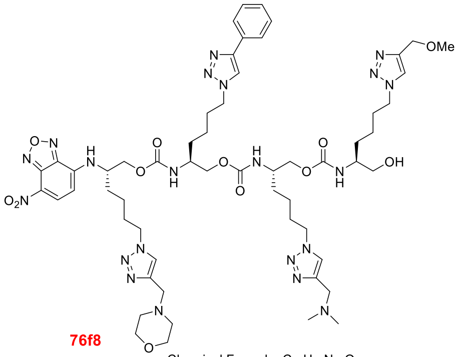

76f8

Chemical Formula: $C_{57}H_{83}N_{21}O_{12}$
Exact Mass: 1253.65

MS Zoomed Spectrum

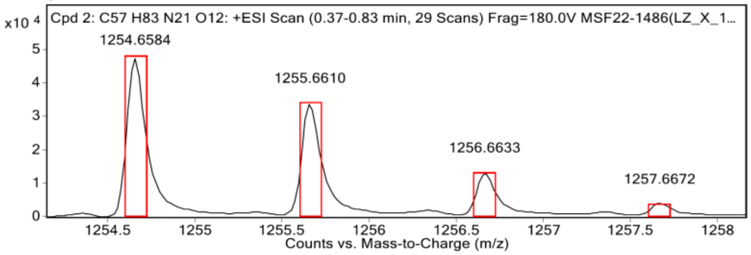

MS Spectrum Peak List

Obs. m/z	Calc. m/z	Charge	Abundance	Formula	Ion Species	Tgt Mass Error (ppm)
268.1768			810816			
1254.6584	1254.6603	1	47537	C57H83N21O12	(M+H)+	1.51
1255.6610	1255.6630	1	34020	C57H83N21O12	(M+H)+	1.59
1256.6633	1256.6656	1	13186	C57H83N21O12	(M+H)+	1.8
1257.6672	1257.6681	1	3804	C57H83N21O12	(M+H)+	0.74
1258.6924	1258.6706	1	931	C57H83N21O12	(M+H)+	-17.35

--- End Of Report ---

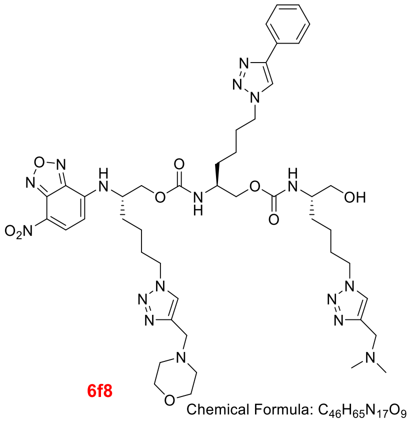

6f8

Chemical Formula: $C_{46}H_{65}N_{17}O_9$
Exact Mass: 999.52

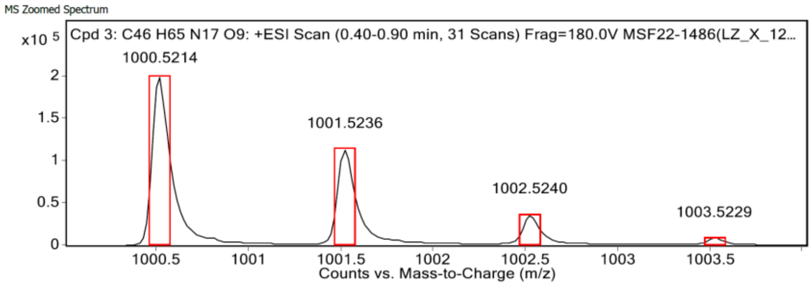

MS Spectrum Peak List

Obs. m/z	Calc. m/z	Charge	Abundance	Formula	Ion Species	Tgt Mass Error (ppm)
268.1768			671398			
1000.5214	1000.5224	1	200279	C46H65N17O9	(M+H)+	0.95
1001.5236	1001.5251	1	113484	C46H65N17O9	(M+H)+	1.47
1002.5240	1002.5277	1	35910	C46H65N17O9	(M+H)+	3.71
1003.5229	1003.5302	1	7840	C46H65N17O9	(M+H)+	7.26
1004.4888	1004.5326	1	2248	C46H65N17O9	(M+H)+	43.6

--- End Of Report ---

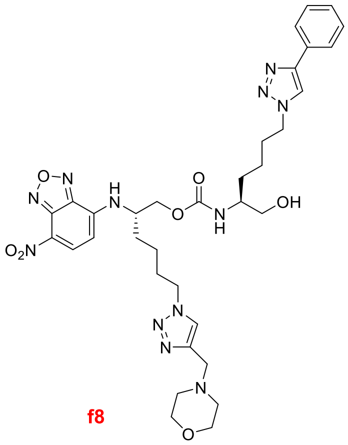

f8

Chemical Formula: $C_{34}H_{44}N_{12}O_7$
Exact Mass: 732.35

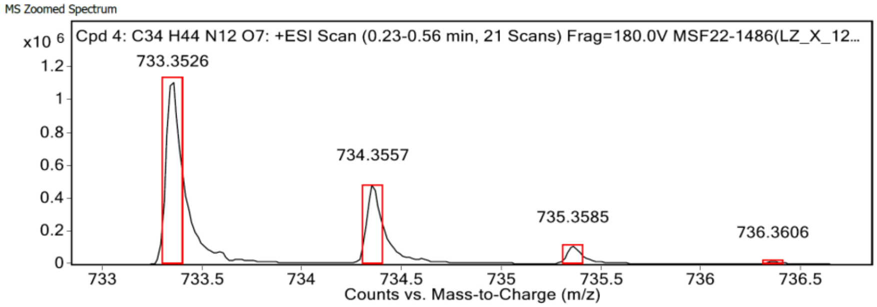

MS Spectrum Peak List

Obs. m/z	Calc. m/z	Charge	Abundance	Formula	Ion Species	Tgt Mass Error (ppm)
733.3526	733.3529	1	1126879	C34H44N12O7	(M+H)+	0.43
734.3557	734.3556	1	489317	C34H44N12O7	(M+H)+	-0.1
735.3585	735.3581	1	110820	C34H44N12O7	(M+H)+	-0.46
736.3606	736.3606	1	17291	C34H44N12O7	(M+H)+	0.08
737.3570	737.3630	1	2994	C34H44N12O7	(M+H)+	8.2
738.3585	738.3654	1	858	C34H44N12O7	(M+H)+	9.38

--- End Of Report ---

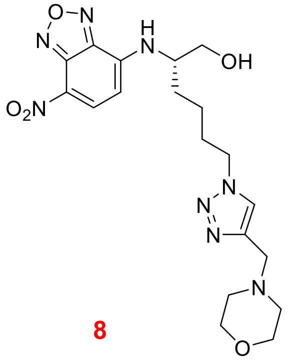

8

Chemical Formula: $C_{19}H_{26}N_8O_5$
Exact Mass: 446.20

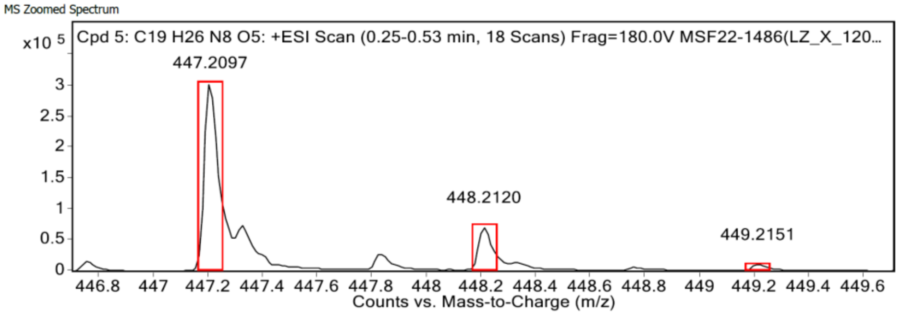

MS Spectrum Peak List

Obs. m/z	Calc. m/z	Charge	Abundance	Formula	Ion Species	Tgt Mass Error (ppm)
447.2097	447.2099	1	305704	C19H26N8O5	(M+H)+	0.46
448.2120	448.2125	1	70604	C19H26N8O5	(M+H)+	1.21
449.2151	449.2148	1	10564	C19H26N8O5	(M+H)+	-0.6
450.2183	450.2172	1	1816	C19H26N8O5	(M+H)+	-2.56
733.3526			1270069			

--- End Of Report ---

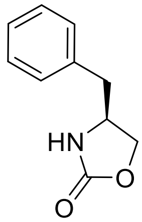

*

Chemical Formula: $C_{10}H_{11}NO_2$
Exact Mass: 177.08

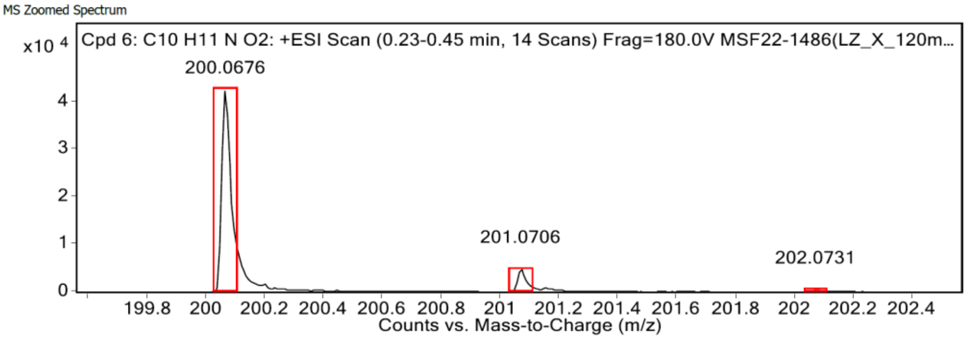

MS Spectrum Peak List

Obs. m/z	Calc. m/z	Charge	Abundance	Formula	Ion Species	Tgt Mass Error (ppm)
200.0676	200.0682	1	42671	C10H11NO2	(M+Na)+	3
201.0706	201.0714	1	4777	C10H11NO2	(M+Na)+	3.72
202.0731	202.0737	1	442	C10H11NO2	(M+Na)+	3.13
733.3523			1368605			

--- End Of Report ---

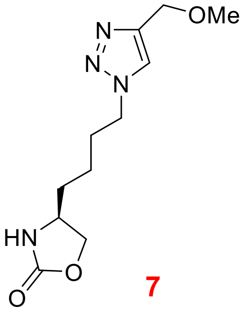

7

Chemical Formula: $C_{11}H_{18}N_4O_3$
Exact Mass: 254.14

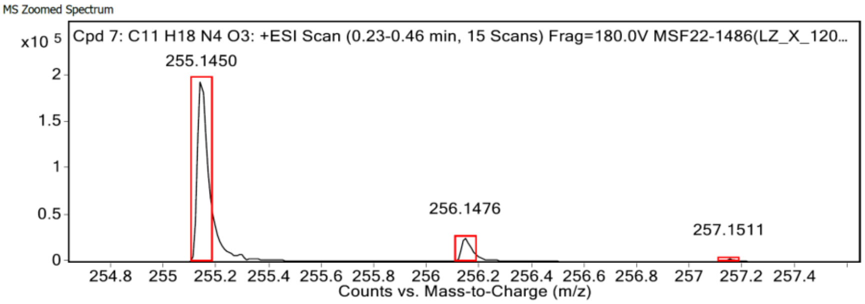

MS Spectrum Peak List

Obs. m/z	Calc. m/z	Charge	Abundance	Formula	Ion Species	Tgt Mass Error (ppm)
255.1450	255.1452	1	197367	C11H18N4O3	(M+H)+	0.72
256.1476	256.1479	1	25928	C11H18N4O3	(M+H)+	1.16
257.1511	257.1501	1	3100	C11H18N4O3	(M+H)+	-3.98
258.1586	258.1524	1	426	C11H18N4O3	(M+H)+	-23.88
733.3524			1352653			

--- End Of Report ---

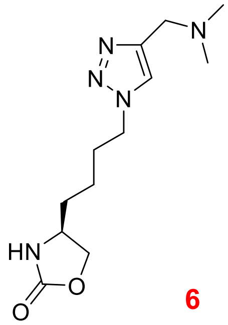

6

Chemical Formula: $C_{12}H_{21}N_5O_2$
Exact Mass: 267.17

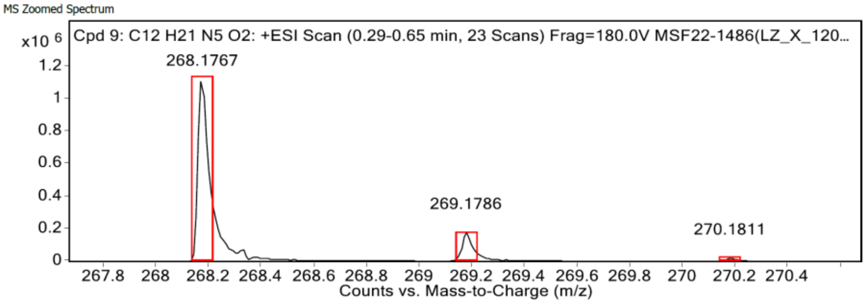

MS Spectrum Peak List

Obs. m/z	Calc. m/z	Charge	Abundance	Formula	Ion Species	Tgt Mass Error (ppm)
268.1767	268.1768	1	1121969	C12H21N5O2	(M+H)+	0.31
269.1786	269.1794	1	176131	C12H21N5O2	(M+H)+	3.1
270.1811	270.1818	1	18622	C12H21N5O2	(M+H)+	2.3
271.1675	271.1841	1	3471	C12H21N5O2	(M+H)+	61.2

--- End Of Report ---

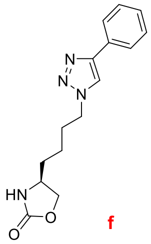

f

Chemical Formula: $C_{15}H_{18}N_4O_2$
Exact Mass: 286.14

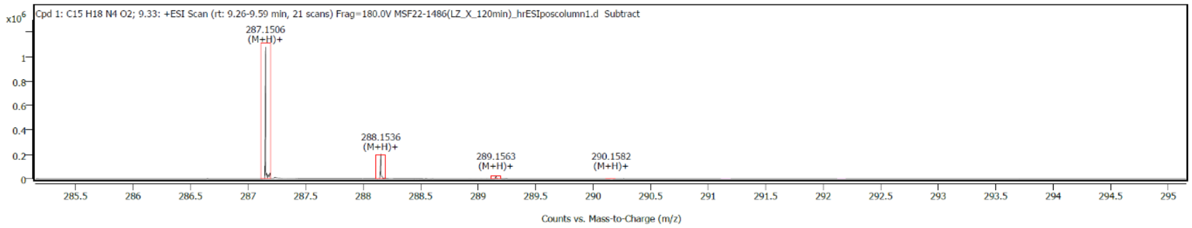

Spectrum Peaks

Obs. m/z	Calc. m/z	Charge	Abund	Formula	Ion Species	Tgt Mass Error (PPM)
287.1506	287.1503	1	1100940	C15H18N4O2	(M+H)+	1.14
288.1536	288.1531	1	205374	C15H18N4O2	(M+H)+	1.73
289.1563	289.1557	1	22564	C15H18N4O2	(M+H)+	2.31
290.1582	290.1581	1	1424	C15H18N4O2	(M+H)+	0.22
733.3531			1177512			

MassHunter Qual 10.0
(End of Report)

Oligomer 6_2: *76f8

***76f8**

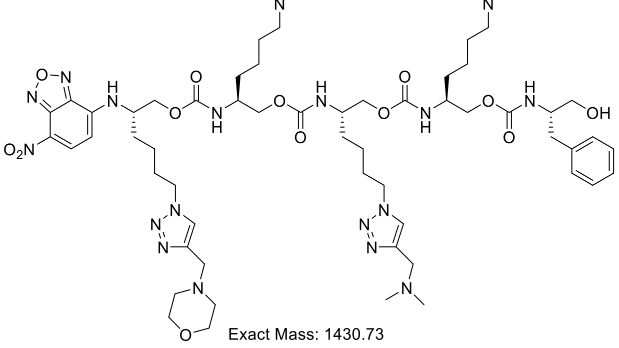

Exact Mass: 1430.73

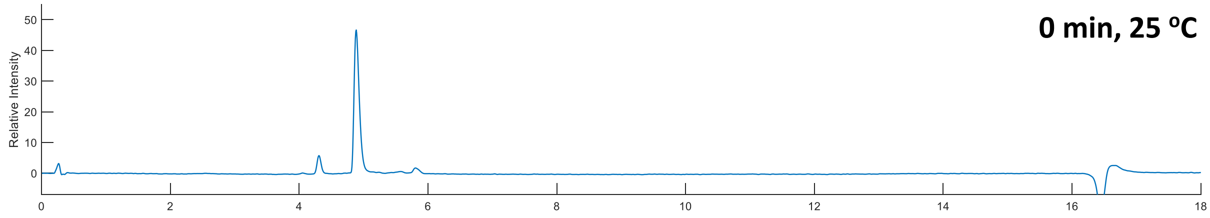

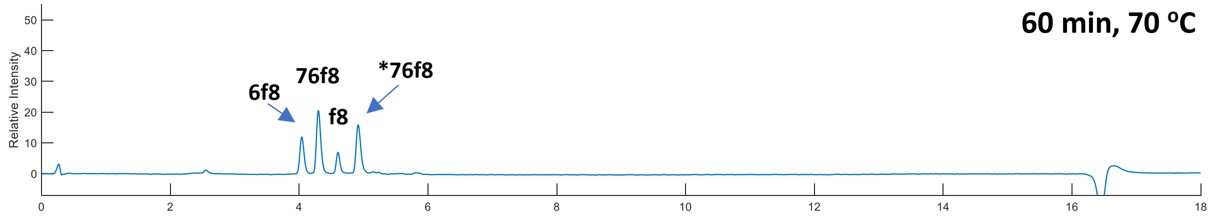

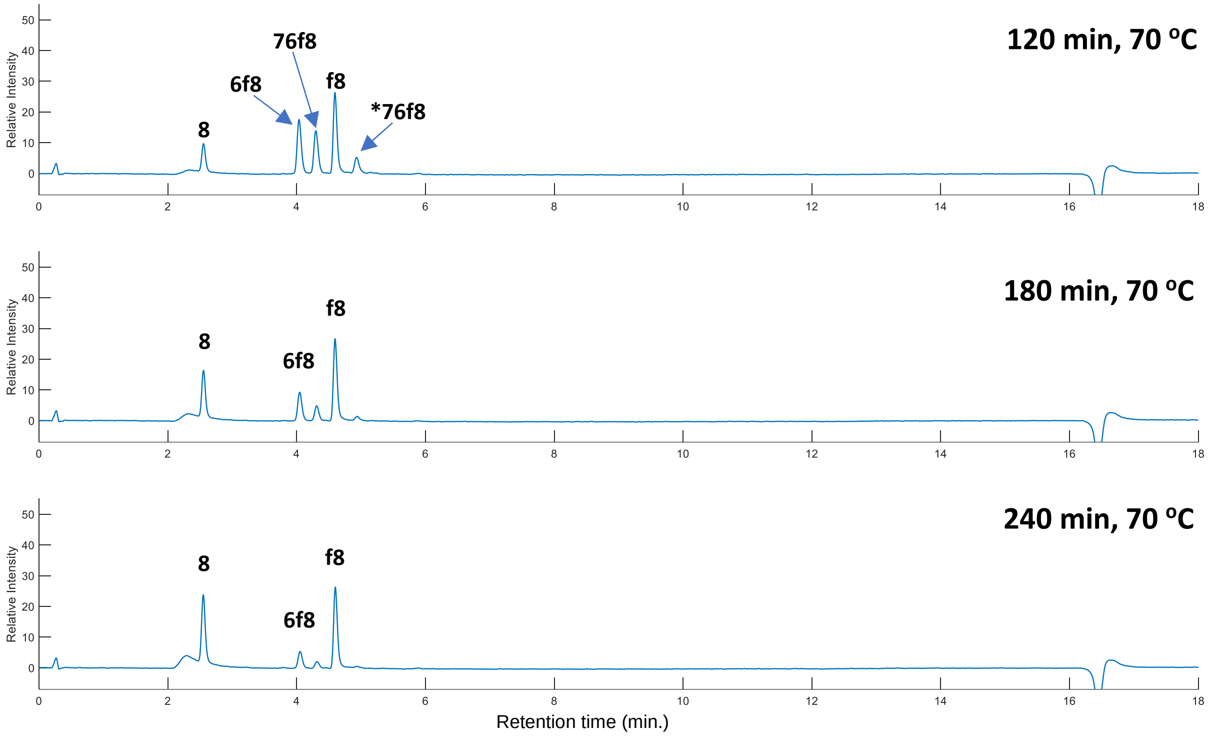

Oligomer Z1_1: *YI

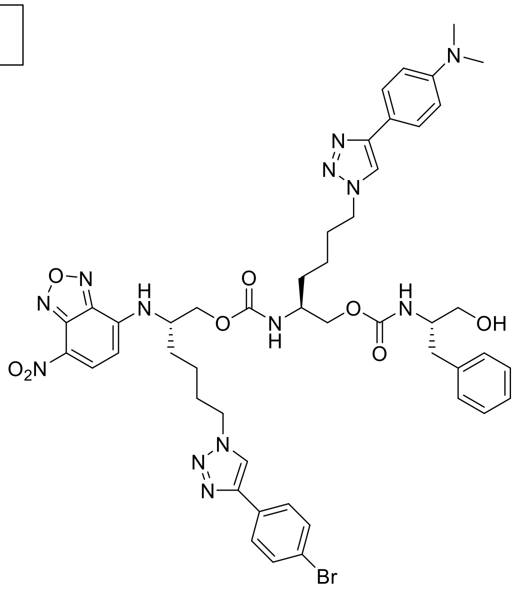

Exact Mass: 1007.34

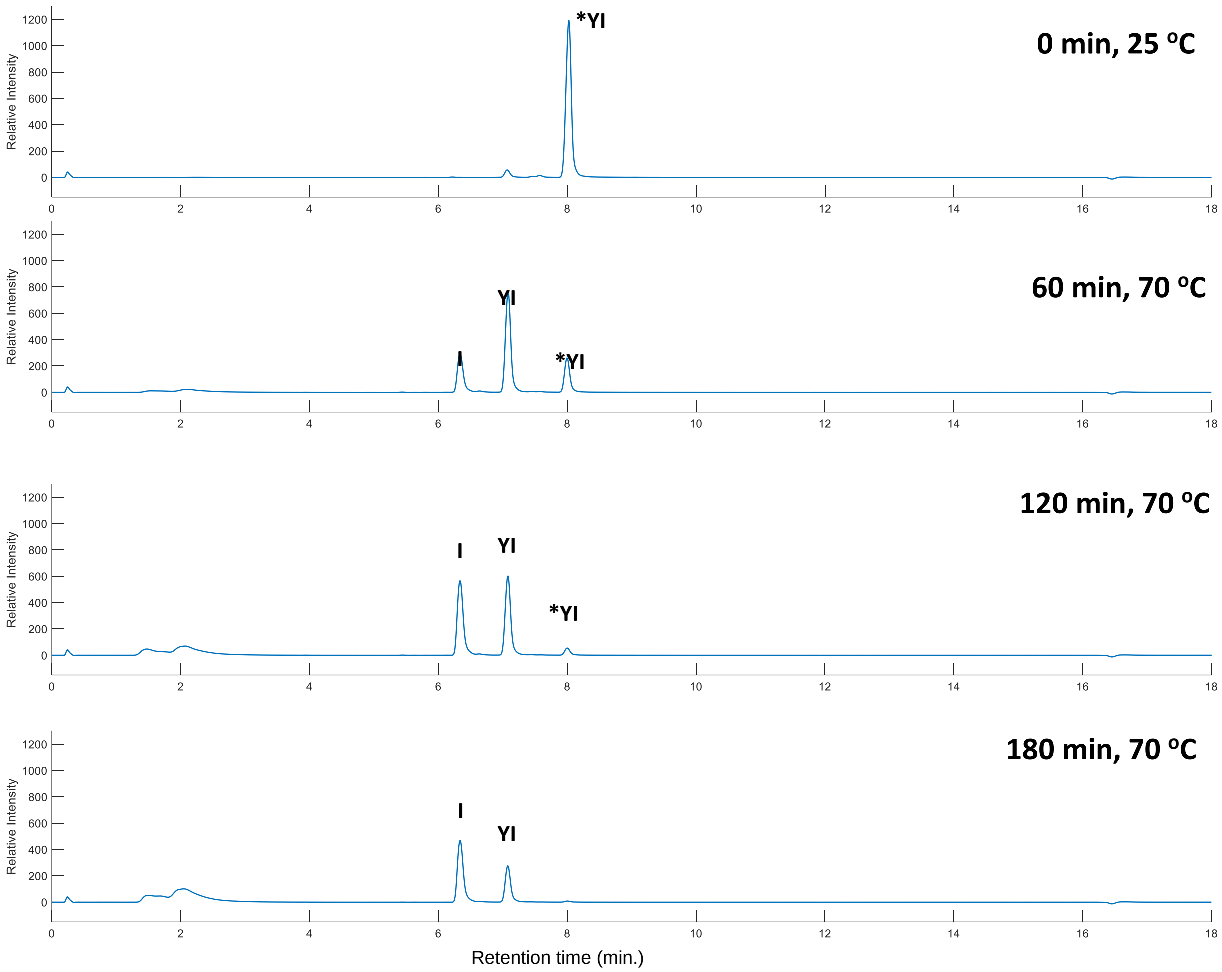

After 60 minutes of sequencing, all the following molecules are observed in the solution and analyzed via High-Res MS.

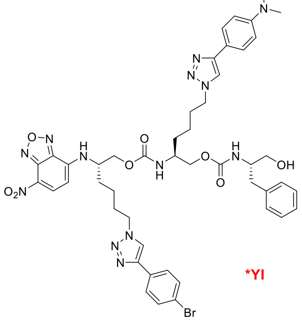
*YI
Chemical Formula: $C_{47}H_{54}BrN_{13}O_8$
Exact Mass: 1007.34

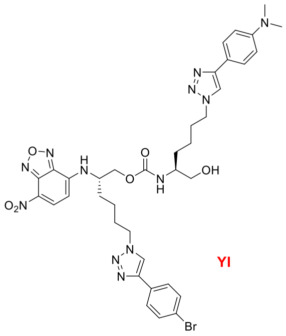
YI
Chemical Formula: $C_{37}H_{43}BrN_{12}O_6$
Exact Mass: 830.26

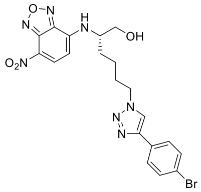
I
Chemical Formula: $C_{20}H_{20}BrN_7O_4$
Exact Mass: 501.08

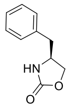
Chemical Formula: $C_{10}H_{11}NO_2$
Exact Mass: 177.08
*

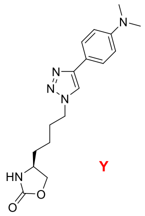
Y
Chemical Formula: $C_{17}H_{23}N_5O_2$
Exact Mass: 329.19

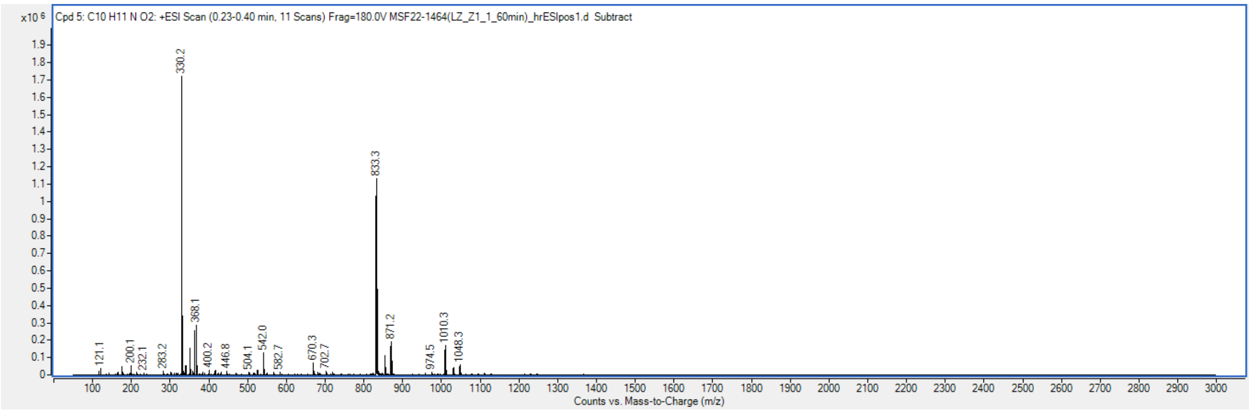

MS Zoomed Spectrum

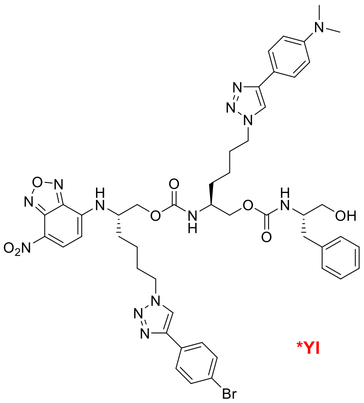

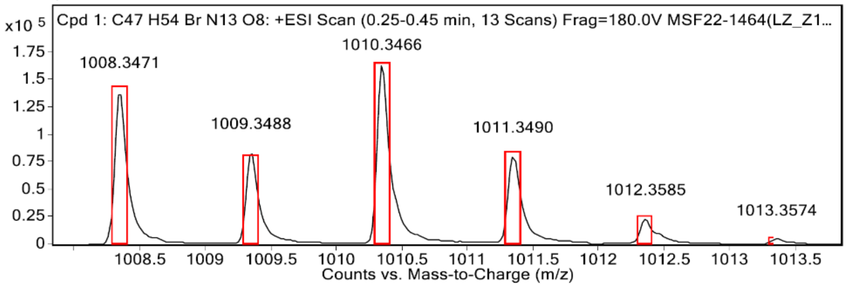

*YI

Chemical Formula: $C_{47}H_{54}BrN_{13}O_8$
Exact Mass: 1007.34

MS Spectrum Peak List

Obs. m/z	Calc. m/z	Charge	Abundance	Formula	Ion Species	Tgt Mass Error (ppm)
330.1927			1631671			
1008.3471	1008.3474	1	139751	C47H54BrN13O8	(M+H)+	0.32
1009.3488	1009.3503	1	84139	C47H54BrN13O8	(M+H)+	1.51
1010.3466	1010.3466	1	164479	C47H54BrN13O8	(M+H)+	-0.02
1011.3490	1011.3487	1	80795	C47H54BrN13O8	(M+H)+	-0.25
1012.3585	1012.3512	1	23911	C47H54BrN13O8	(M+H)+	-7.14
1013.3574	1013.3538	1	5160	C47H54BrN13O8	(M+H)+	-3.59

--- End Of Report ---

MS Zoomed Spectrum

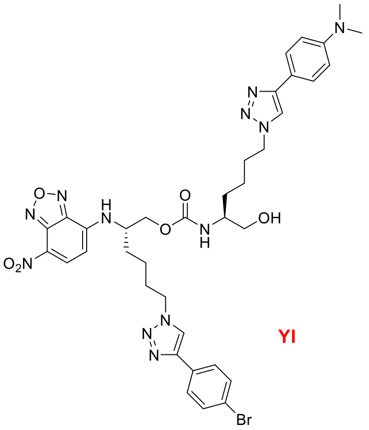

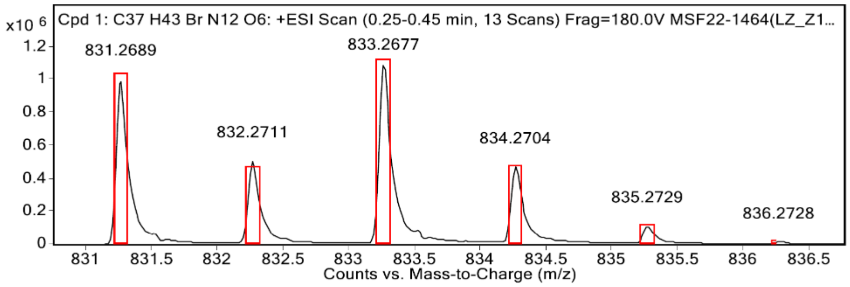

YI

Chemical Formula: $C_{37}H_{43}BrN_{12}O_6$
Exact Mass: 830.26

MS Spectrum Peak List

Obs. m/z	Calc. m/z	Charge	Abundance	Formula	Ion Species	Tgt Mass Error (ppm)
330.1927			1631767			
831.2689	831.2685	1	1006838	C37H43BrN12O6	(M+H)+	-0.48
832.2711	832.2712	1	509775	C37H43BrN12O6	(M+H)+	0.18
833.2677	833.2672	1	1108627	C37H43BrN12O6	(M+H)+	-0.59
834.2704	834.2695	1	473370	C37H43BrN12O6	(M+H)+	-1.07
835.2729	835.2720	1	114724	C37H43BrN12O6	(M+H)+	-1.11
836.2728	836.2745	1	16771	C37H43BrN12O6	(M+H)+	1.97
837.2740	837.2769	1	2459	C37H43BrN12O6	(M+H)+	3.53

--- End Of Report ---

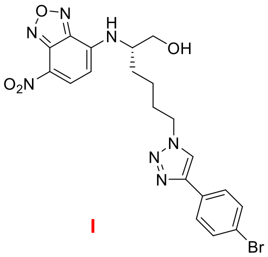

I

Chemical Formula: $C_{20}H_{20}BrN_7O_4$
Exact Mass: 501.08

MS Zoomed Spectrum

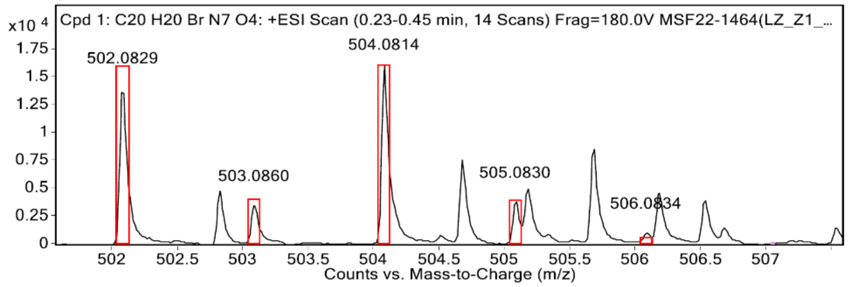

MS Spectrum Peak List

Obs. m/z	Calc. m/z	Charge	Abundance	Formula	Ion Species	Tgt Mass Error (ppm)
330.1927			1516400			
502.0829	502.0833	1	14174	C20H20BrN7O4	(M+H)+	0.78
503.0860	503.0860	1	3568	C20H20BrN7O4	(M+H)+	-0.03
504.0814	504.0815	1	16105	C20H20BrN7O4	(M+H)+	0.14
505.0830	505.0841	1	3865	C20H20BrN7O4	(M+H)+	2.07
506.0834	506.0865	1	1086	C20H20BrN7O4	(M+H)+	6.12

--- End Of Report ---

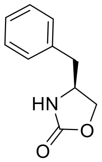

Chemical Formula: $C_{10}H_{11}NO_2$
Exact Mass: 177.08
*

MS Zoomed Spectrum

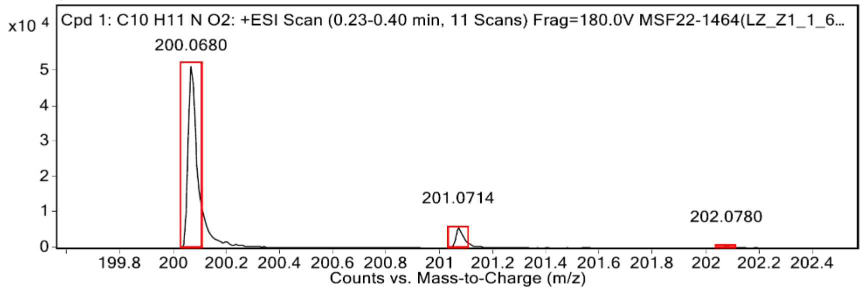

MS Spectrum Peak List

Obs. m/z	Calc. m/z	Charge	Abundance	Formula	Ion Species	Tgt Mass Error (ppm)
200.0680	200.0682	1	52132	C10H11NO2	(M+Na)+	0.88
201.0714	201.0714	1	5911	C10H11NO2	(M+Na)+	0.04
202.0780	202.0737	1	628	C10H11NO2	(M+Na)+	-21.08
330.1926			1735902			

--- End Of Report ---

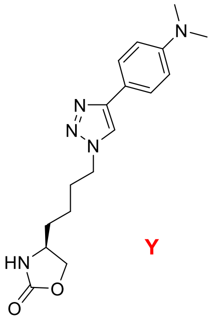

Y

Chemical Formula: $C_{17}H_{23}N_5O_2$
Exact Mass: 329.19

MS Zoomed Spectrum

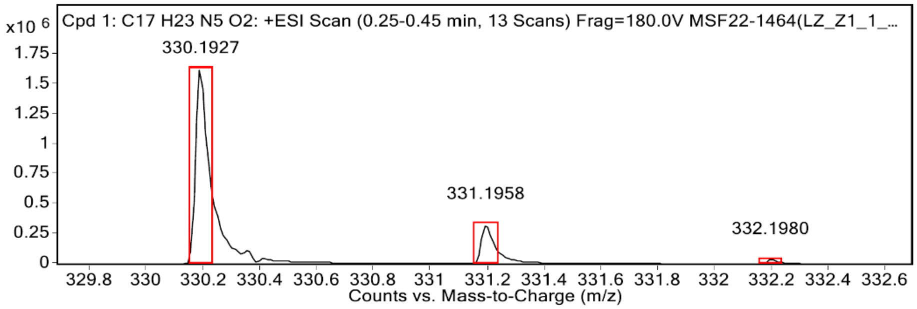

MS Spectrum Peak List

Obs. m/z	Calc. m/z	Charge	Abundance	Formula	Ion Species	Tgt Mass Error (ppm)
330.1927	330.1925	1	1631610	C17H23N5O2	(M+H)+	-0.83
331.1958	331.1953	1	329494	C17H23N5O2	(M+H)+	-1.67
332.1980	332.1979	1	36961	C17H23N5O2	(M+H)+	-0.44
333.2098	333.2003	1	5255	C17H23N5O2	(M+H)+	-28.5
334.2105	334.2028	1	1033	C17H23N5O2	(M+H)+	-23.2

--- End Of Report ---

Oligomer Z1_2: *YI

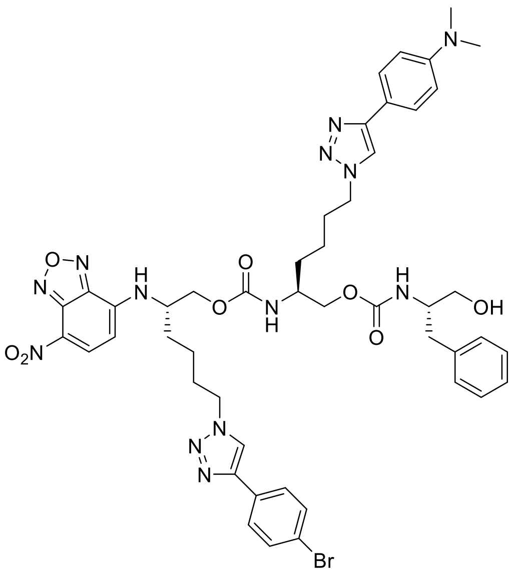

Exact Mass: 1007.34

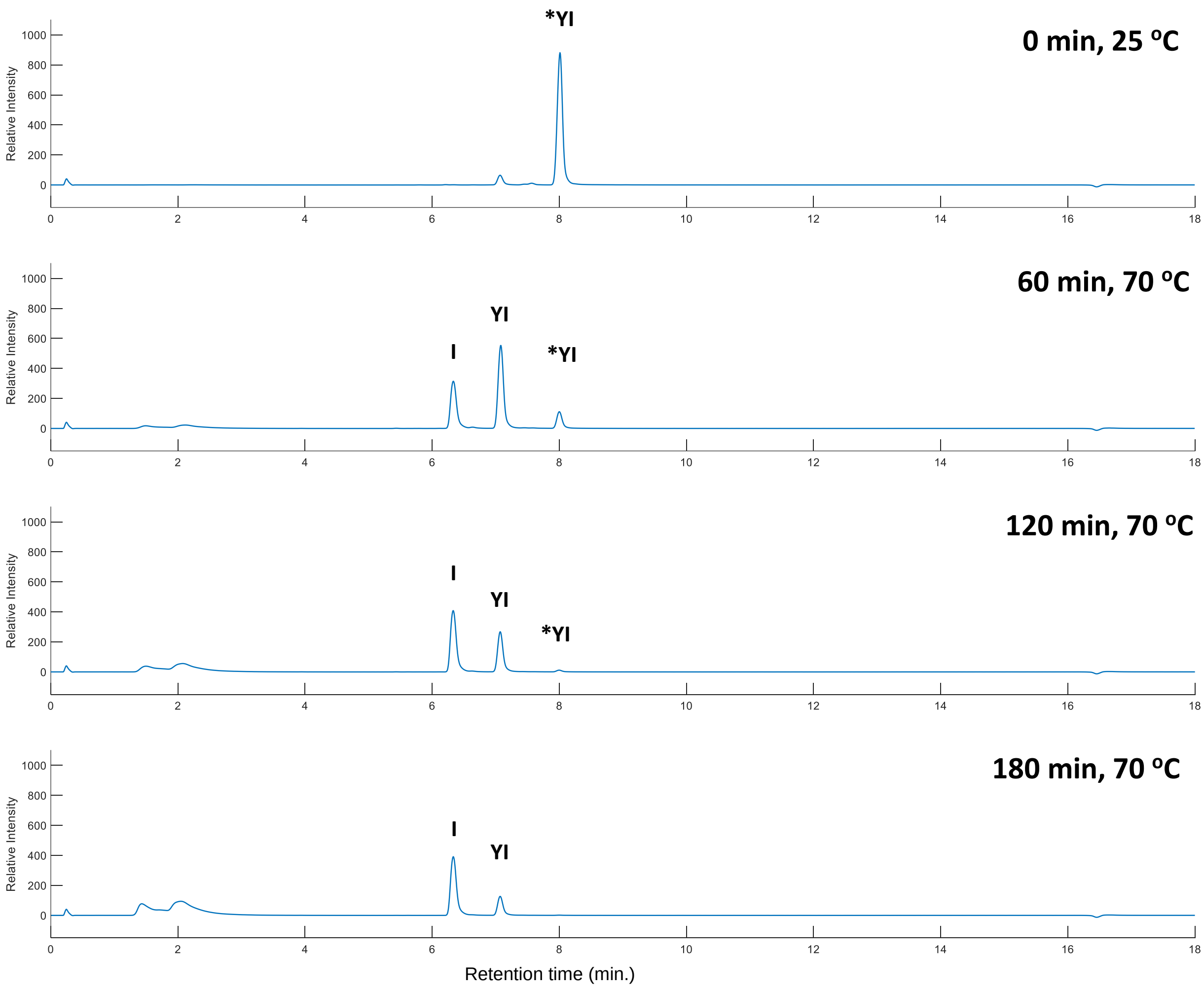

Oligomer Z2: *YT

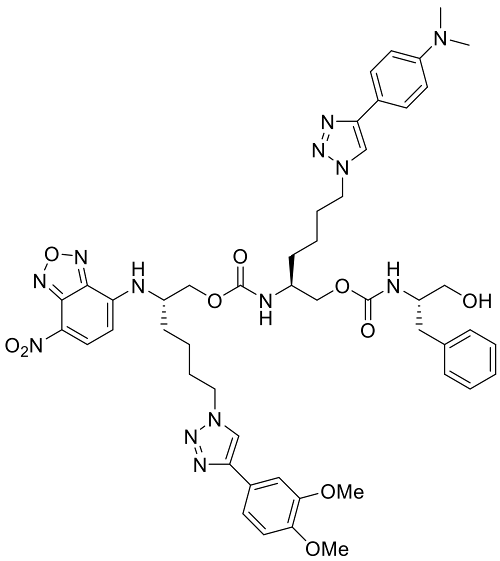

Exact Mass: 989.45

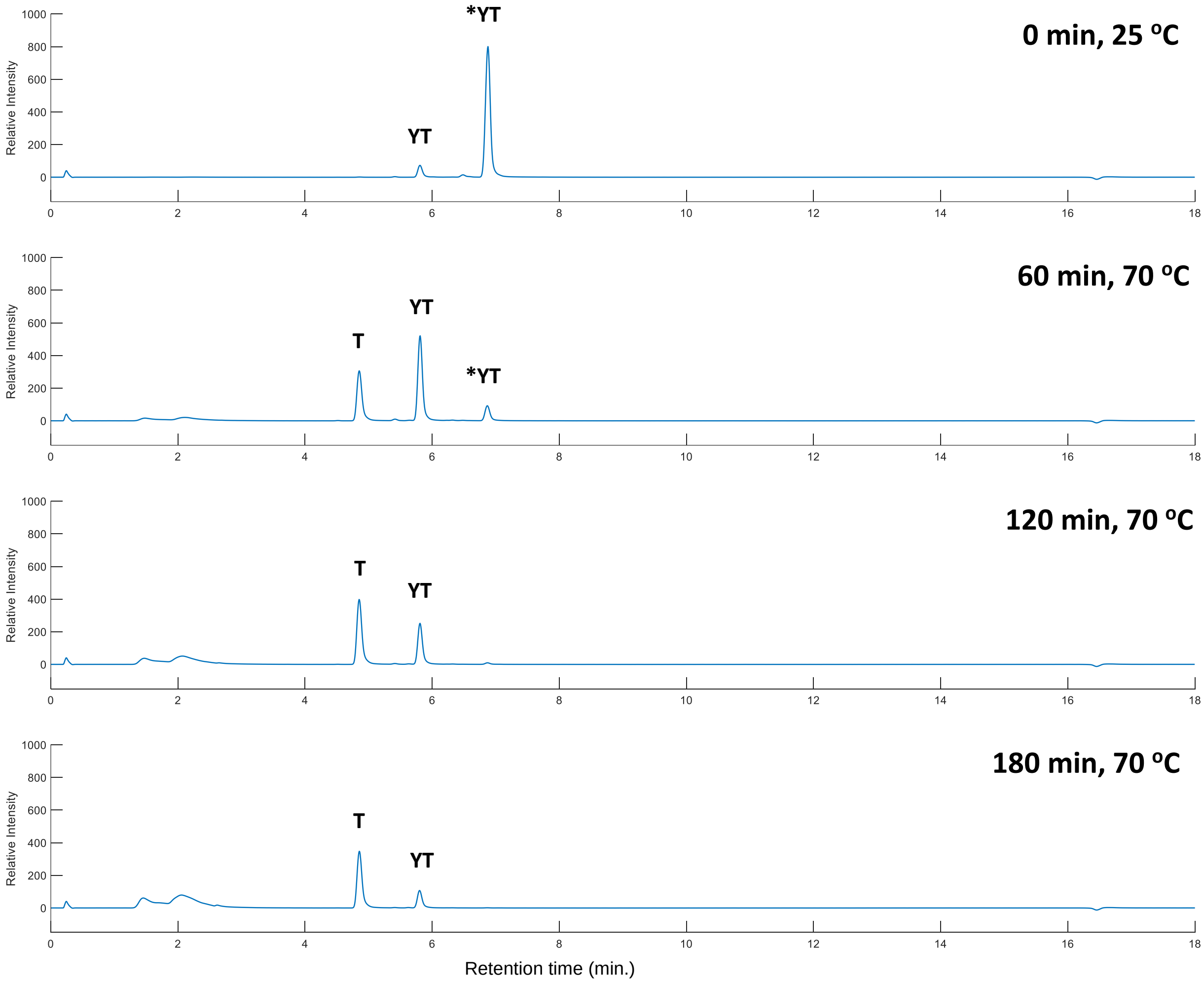

After 60 minutes of sequencing, all the following molecules are observed in the solution and analyzed via High-Res MS.

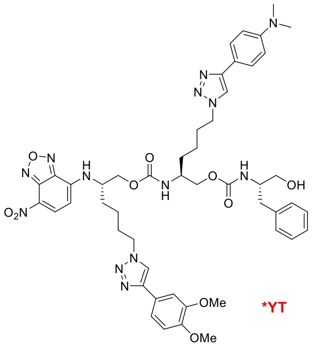

*YT

Chemical Formula: $C_{49}H_{59}N_{13}O_{10}$
Exact Mass: 989.45

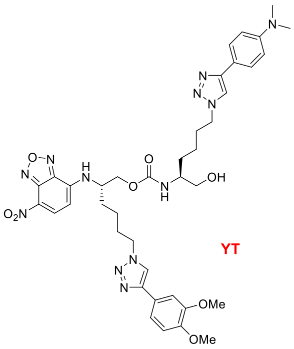

YT

Chemical Formula: $C_{39}H_{48}N_{12}O_8$
Exact Mass: 812.37

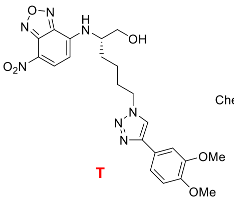

T

Chemical Formula: $C_{22}H_{25}N_7O_6$
Exact Mass: 483.19

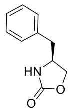

Chemical Formula: $C_{10}H_{11}NO_2$
Exact Mass: 177.08

*

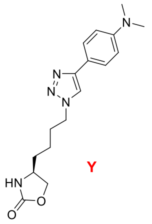

Y

Chemical Formula: $C_{17}H_{23}N_5O_2$
Exact Mass: 329.19

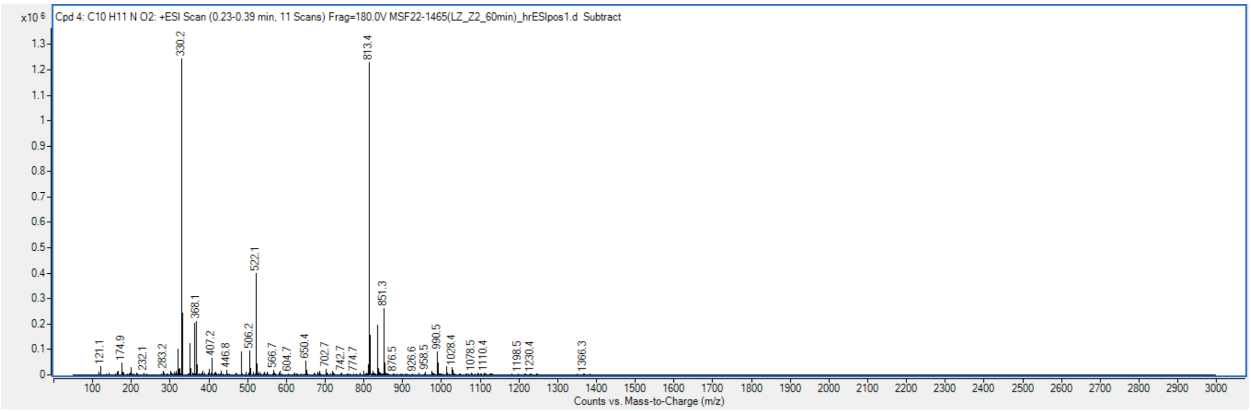

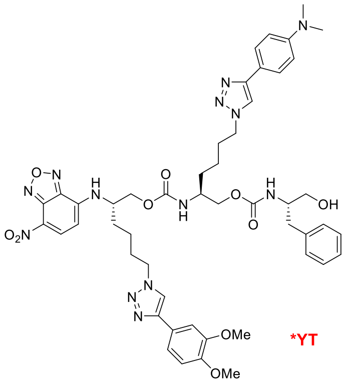

*YT

Chemical Formula: $C_{49}H_{59}N_{13}O_{10}$
Exact Mass: 989.45

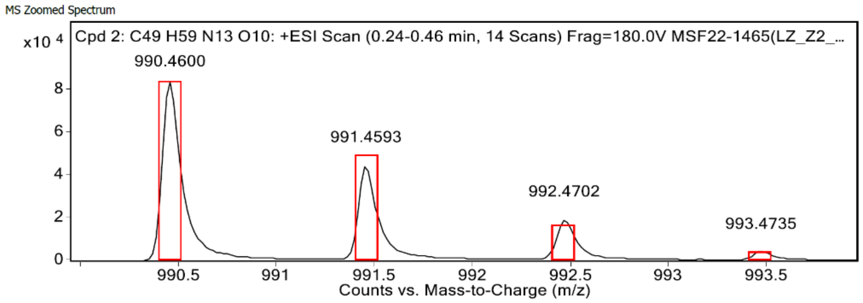

MS Spectrum Peak List

Obs. m/z	Calc. m/z	Charge	Abundance	Formula	Ion Species	Tgt Mass Error (ppm)
813.3784			1139089			
990.4600	990.4581	1	83810	C49H59N13O10	(M+H)+	-1.93
991.4593	991.4609	1	44582	C49H59N13O10	(M+H)+	1.68
992.4702	992.4637	1	19087	C49H59N13O10	(M+H)+	-6.6
993.4735	993.4663	1	3922	C49H59N13O10	(M+H)+	-7.26

--- End Of Report ---

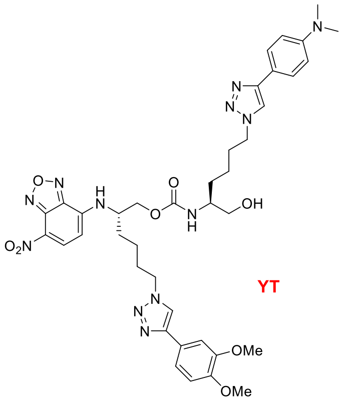

YT

Chemical Formula: $C_{39}H_{48}N_{12}O_8$
Exact Mass: 812.37

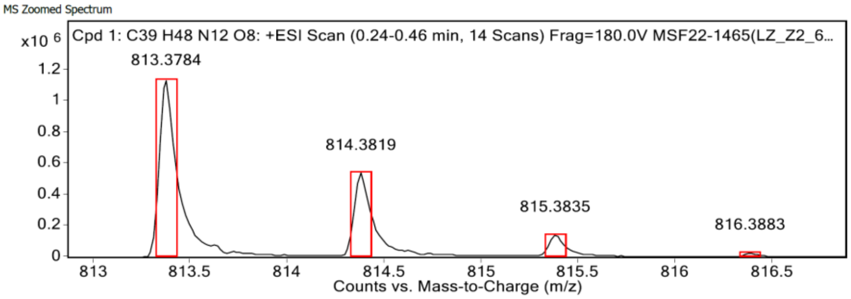

MS Spectrum Peak List

Obs. m/z	Calc. m/z	Charge	Abundance	Formula	Ion Species	Tgt Mass Error (ppm)
813.3784	813.3791	1	1139112	C39H48N12O8	(M+H)+	0.8
814.3819	814.3819	1	538590	C39H48N12O8	(M+H)+	0.02
815.3835	815.3845	1	142767	C39H48N12O8	(M+H)+	1.23
816.3883	816.3870	1	28719	C39H48N12O8	(M+H)+	-1.52
817.3810	817.3895	1	4115	C39H48N12O8	(M+H)+	10.47
818.3698	818.3920	1	824	C39H48N12O8	(M+H)+	27.06

--- End Of Report ---

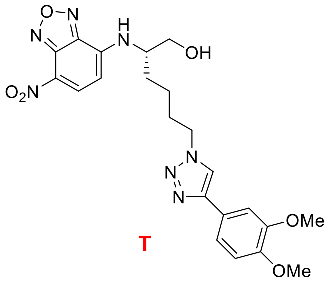

T

Chemical Formula: $C_{22}H_{25}N_7O_6$
Exact Mass: 483.19

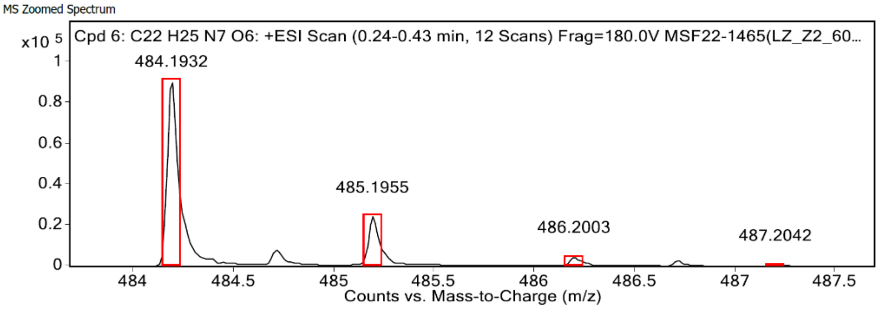

MS Spectrum Peak List

Obs. m/z	Calc. m/z	Charge	Abundance	Formula	Ion Species	Tgt Mass Error (ppm)
484.1932	484.1939	1	91353	C22H25N7O6	(M+H)+	1.52
485.1955	485.1967	1	24078	C22H25N7O6	(M+H)+	2.39
486.2003	486.1991	1	4524	C22H25N7O6	(M+H)+	-2.47
487.2042	487.2015	1	632	C22H25N7O6	(M+H)+	-5.38
813.3784			1274478			

--- End Of Report ---

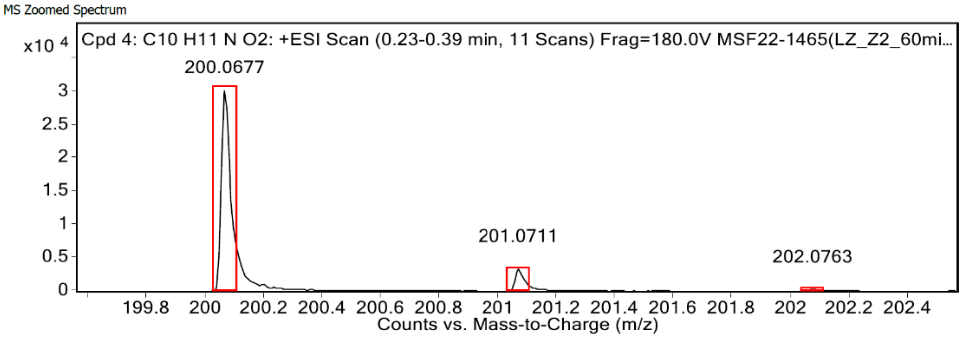

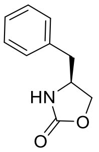

Chemical Formula: $C_{10}H_{11}NO_2$
Exact Mass: 177.08
*

MS Spectrum Peak List

Obs. m/z	Calc. m/z	Charge	Abundance	Formula	Ion Species	Tgt Mass Error (ppm)
200.0677	200.0682	1	30751	C10H11NO2	(M+Na)+	2.41
201.0711	201.0714	1	3465	C10H11NO2	(M+Na)+	1.55
202.0763	202.0737	1	382	C10H11NO2	(M+Na)+	-12.94
330.1922			1253860			

--- End Of Report ---

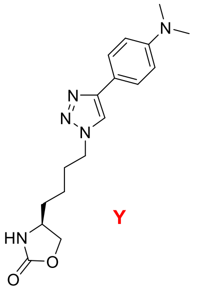

Y

Chemical Formula: $C_{17}H_{23}N_5O_2$
Exact Mass: 329.19

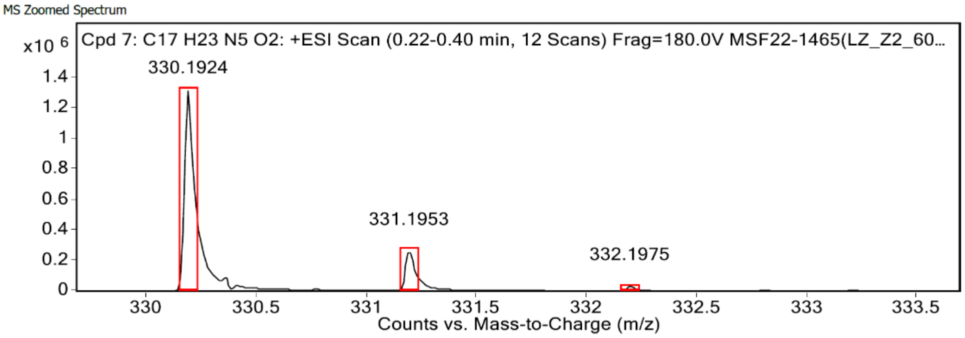

MS Spectrum Peak List

Obs. m/z	Calc. m/z	Charge	Abundance	Formula	Ion Species	Tgt Mass Error (ppm)
330.1924	330.1925	1	1327937	C17H23N5O2	(M+H)+	0.13
331.1953	331.1953	1	263087	C17H23N5O2	(M+H)+	-0.15
332.1975	332.1979	1	29321	C17H23N5O2	(M+H)+	0.98
813.3784			1360110			

--- End Of Report ---

Oligomer Z3: *umc

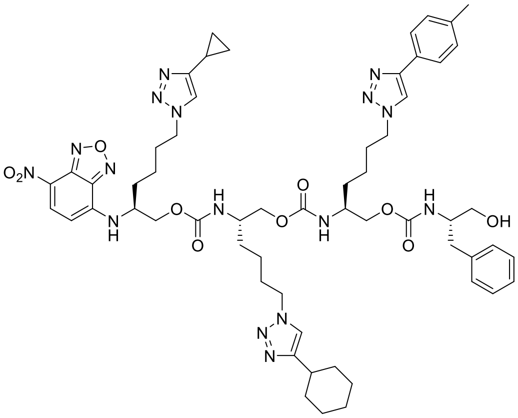

Exact Mass: 1156.59

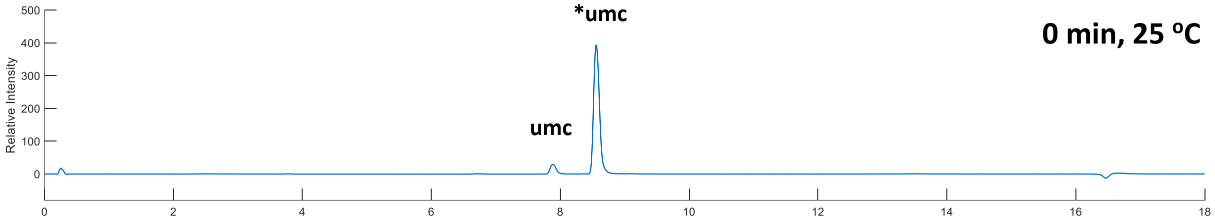

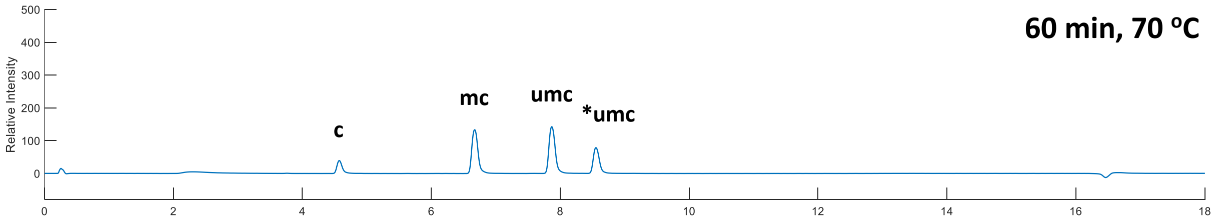

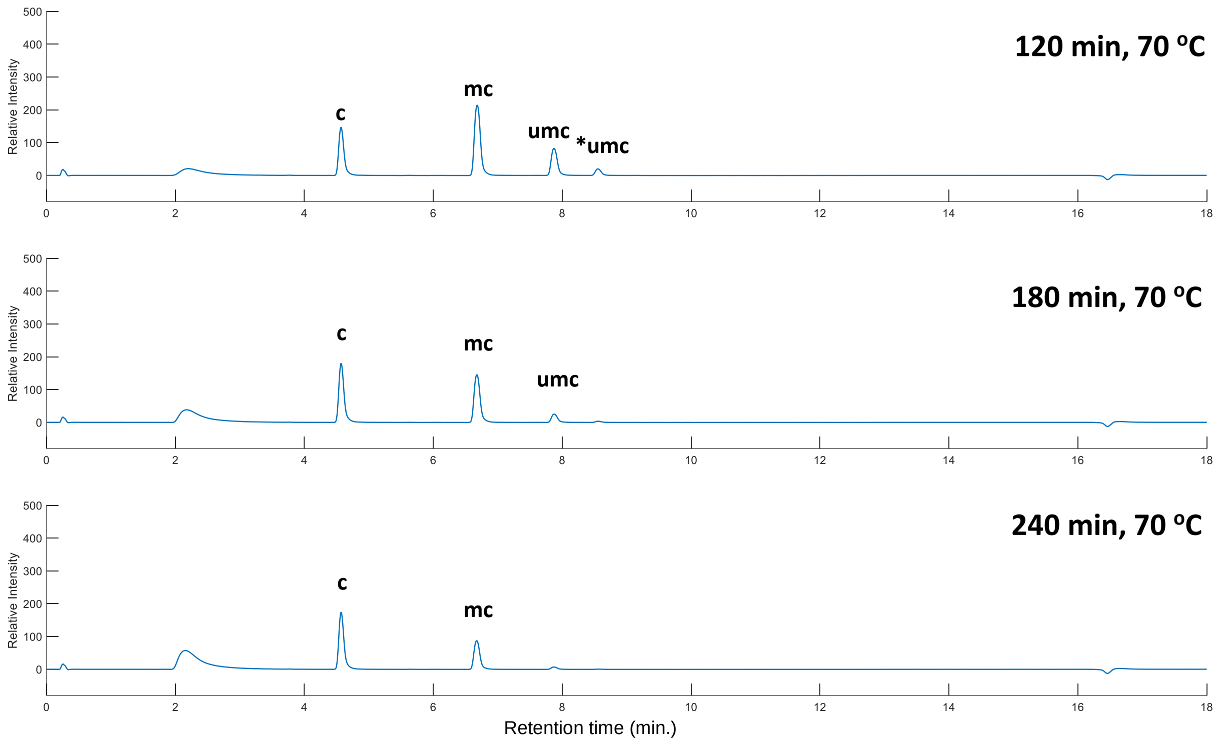

After 60 minutes of sequencing, all the following molecules are observed in the solution and analyzed via High-Res MS.

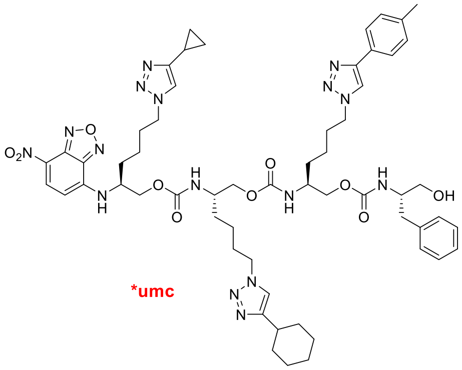

Chemical Formula: $C_{58}H_{76}N_{16}O_{10}$
Exact Mass: 1156.59

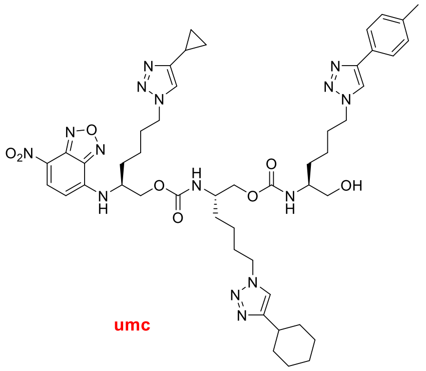

Chemical Formula: $C_{48}H_{65}N_{15}O_8$
Exact Mass: 979.51

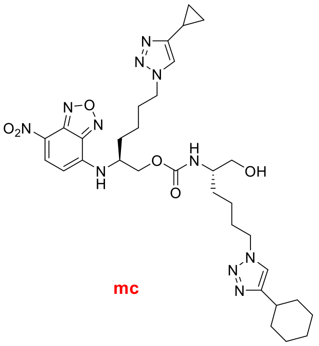

Chemical Formula: $C_{32}H_{45}N_{11}O_6$
Exact Mass: 679.36

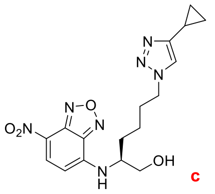

Chemical Formula: $C_{17}H_{21}N_7O_4$
Exact Mass: 387.17

Chemical Formula: $C_{10}H_{11}NO_2$
Exact Mass: 177.08

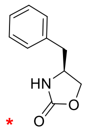

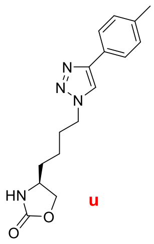

Chemical Formula: $C_{16}H_{20}N_4O_2$
Exact Mass: 300.16

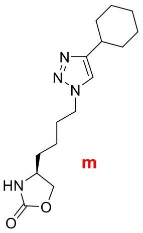

Chemical Formula: $C_{15}H_{24}N_4O_2$
Exact Mass: 292.19

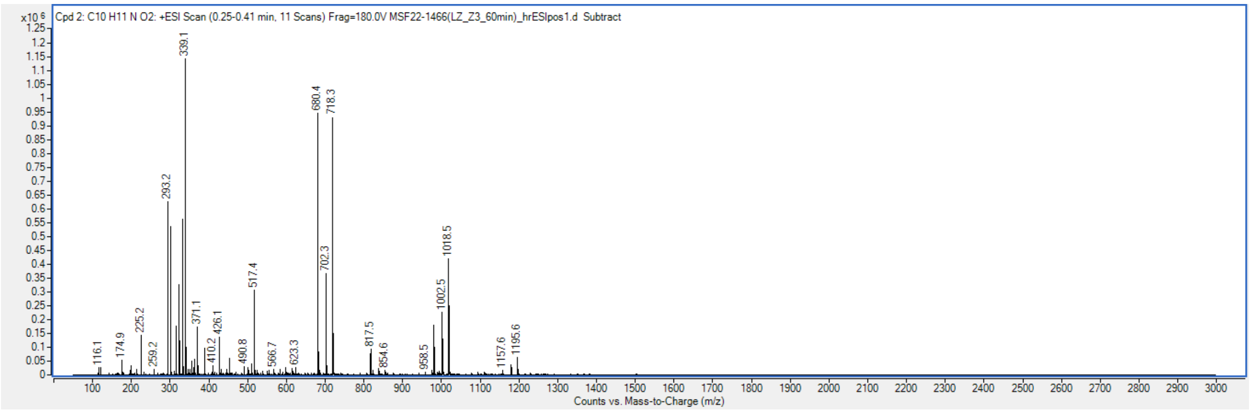

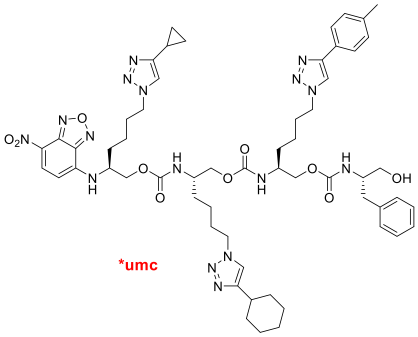

*umc

Chemical Formula: $C_{58}H_{76}N_{16}O_{10}$
Exact Mass: 1156.59

MS Zoomed Spectrum

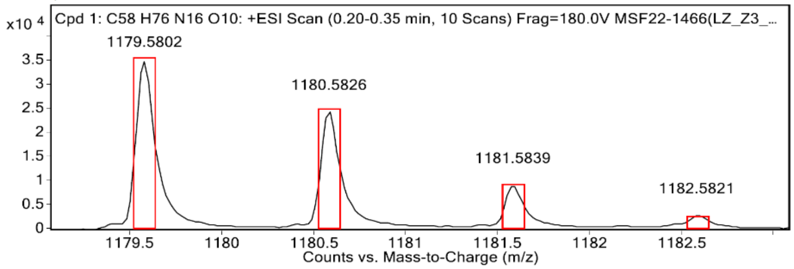

MS Spectrum Peak List

Obs. m/z	Calc. m/z	Charge	Abundance	Formula	Ion Species	Tgt Mass Error (ppm)
339.1217			1248861			
1179.5802	1179.5823	1	34878	C58H76N16O10	(M+Na)+	1.74
1180.5826	1180.5851	1	24409	C58H76N16O10	(M+Na)+	2.14
1181.5839	1181.5879	1	9213	C58H76N16O10	(M+Na)+	3.39
1182.5821	1182.5905	1	2914	C58H76N16O10	(M+Na)+	7.1
1183.5960	1183.5931	1	772	C58H76N16O10	(M+Na)+	-2.43

--- End Of Report ---

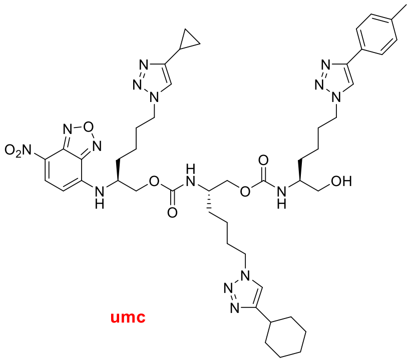

umc

Chemical Formula: $C_{48}H_{65}N_{15}O_8$
Exact Mass: 979.51

MS Zoomed Spectrum

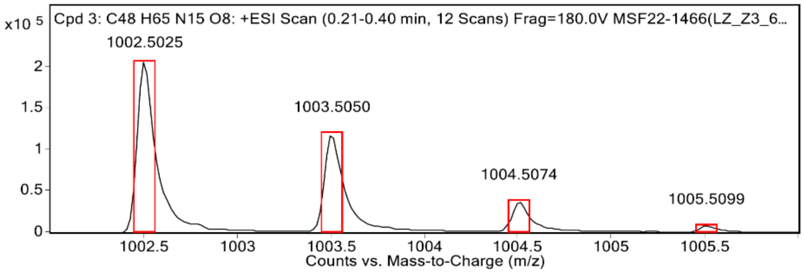

MS Spectrum Peak List

Obs. m/z	Calc. m/z	Charge	Abundance	Formula	Ion Species	Tgt Mass Error (ppm)
339.1217			955617			
1002.5025	1002.5033	1	207380	C48H65N15O8	(M+Na)+	0.75
1003.5050	1003.5061	1	119381	C48H65N15O8	(M+Na)+	1.11
1004.5074	1004.5088	1	37382	C48H65N15O8	(M+Na)+	1.37
1005.5099	1005.5113	1	7696	C48H65N15O8	(M+Na)+	1.47
1006.5170	1006.5139	1	1636	C48H65N15O8	(M+Na)+	-3.09

--- End Of Report ---

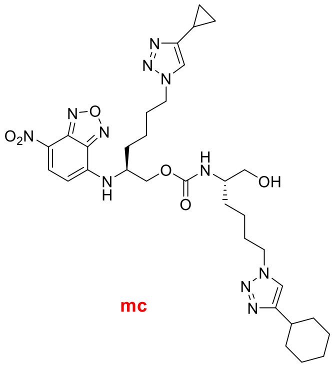

mc

Chemical Formula: $C_{32}H_{45}N_{11}O_6$
Exact Mass: 679.36

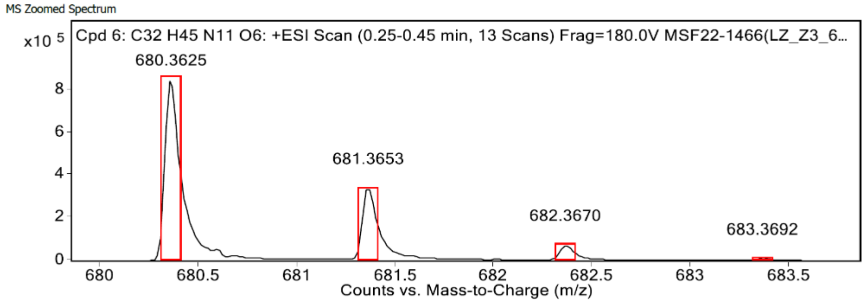

MS Spectrum Peak List

Obs. m/z	Calc. m/z	Charge	Abundance	Formula	Ion Species	Tgt Mass Error (ppm)
339.1218			1143050			
680.3625	680.3627	1	858821	C32H45N11O6	(M+H)+	0.34
681.3653	681.3655	1	341434	C32H45N11O6	(M+H)+	0.23
682.3670	682.3680	1	74733	C32H45N11O6	(M+H)+	1.5
683.3692	683.3705	1	11149	C32H45N11O6	(M+H)+	1.88
684.3711	684.3729	1	1753	C32H45N11O6	(M+H)+	2.72

--- End Of Report ---

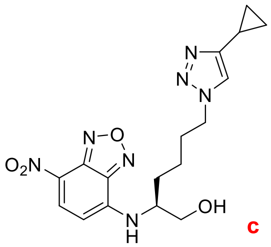

c

Chemical Formula: $C_{17}H_{21}N_7O_4$
Exact Mass: 387.17

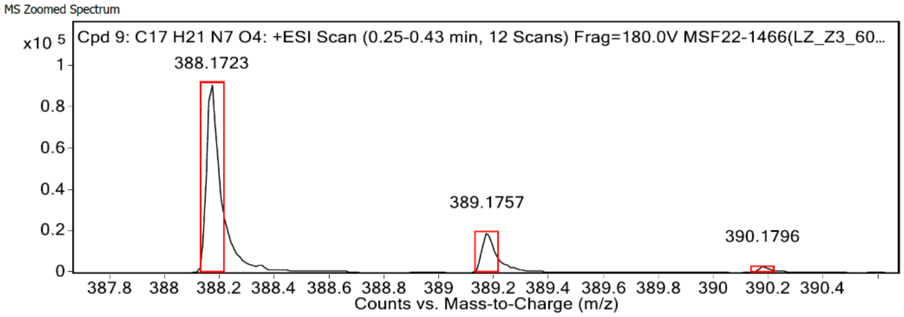

MS Spectrum Peak List

Obs. m/z	Calc. m/z	Charge	Abundance	Formula	Ion Species	Tgt Mass Error (ppm)
339.1218			1172686			
388.1723	388.1728	1	91609	C17H21N7O4	(M+H)+	1.27
389.1757	389.1754	1	19532	C17H21N7O4	(M+H)+	-0.81
390.1796	390.1777	1	3009	C17H21N7O4	(M+H)+	-4.64

--- End Of Report ---

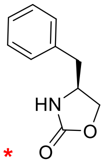

*

Chemical Formula: $C_{10}H_{11}NO_2$
Exact Mass: 177.08

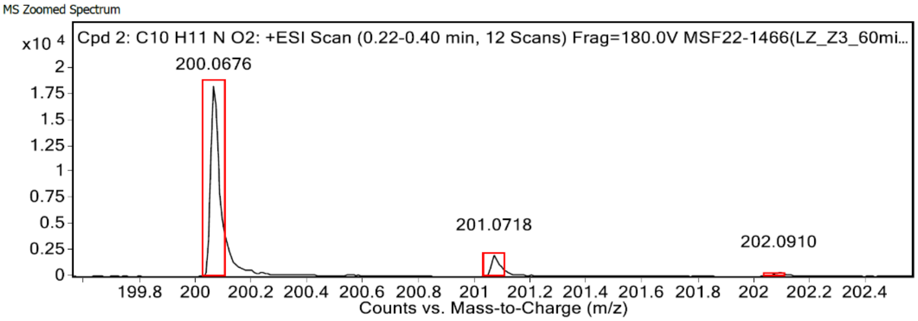

MS Spectrum Peak List

Obs. m/z	Calc. m/z	Charge	Abundance	Formula	Ion Species	Tgt Mass Error (ppm)
200.0676	200.0682	1	18592	C10H11NO2	(M+Na)+	2.95
201.0718	201.0714	1	2041	C10H11NO2	(M+Na)+	-1.88
202.0910	202.0737	1	414	C10H11NO2	(M+Na)+	-85.41
339.1217			1652829			

--- End Of Report ---

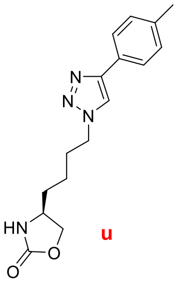

u

Chemical Formula: $C_{16}H_{20}N_4O_2$
Exact Mass: 300.16

MS Zoomed Spectrum

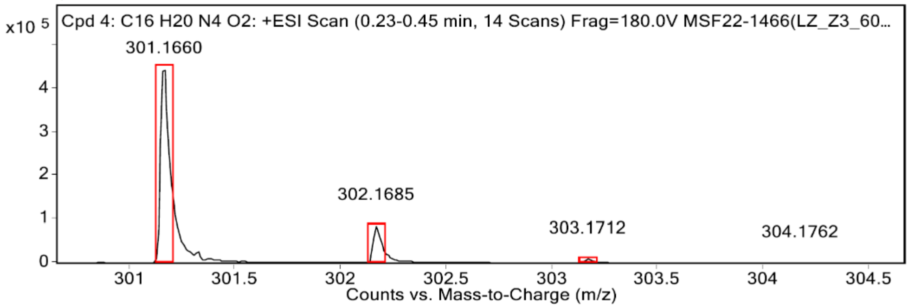

MS Spectrum Peak List

Obs. m/z	Calc. m/z	Charge	Abundance	Formula	Ion Species	Tgt Mass Error (ppm)
301.1660	301.1659	1	452094	C16H20N4O2	(M+H)+	-0.47
302.1685	302.1688	1	85743	C16H20N4O2	(M+H)+	0.99
303.1712	303.1714	1	9452	C16H20N4O2	(M+H)+	0.69
304.1762	304.1739	1	828	C16H20N4O2	(M+H)+	-7.61
339.1218			1080400			

--- End Of Report ---

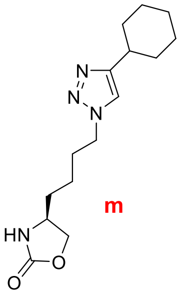

m

Chemical Formula: $C_{15}H_{24}N_4O_2$
Exact Mass: 292.19

MS Zoomed Spectrum

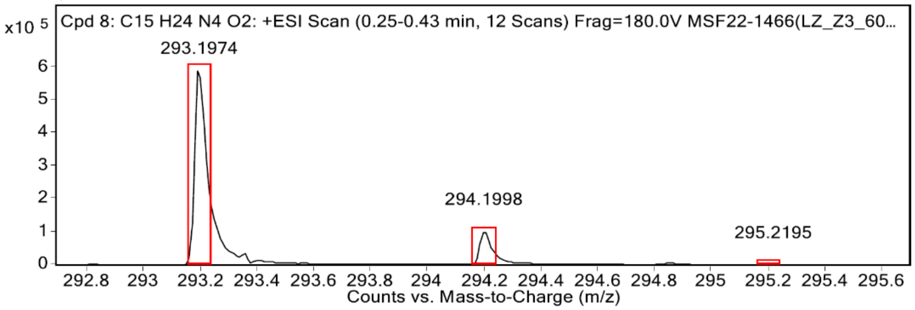

MS Spectrum Peak List

Obs. m/z	Calc. m/z	Charge	Abundance	Formula	Ion Species	Tgt Mass Error (ppm)
293.1974	293.1972	1	607055	C15H24N4O2	(M+H)+	-0.74
294.1998	294.2001	1	104470	C15H24N4O2	(M+H)+	1.04
295.2195	295.2026	1	3072	C15H24N4O2	(M+H)+	-56.96
339.1218			1178658			

--- End Of Report ---

Oligomer Z4: *pdw

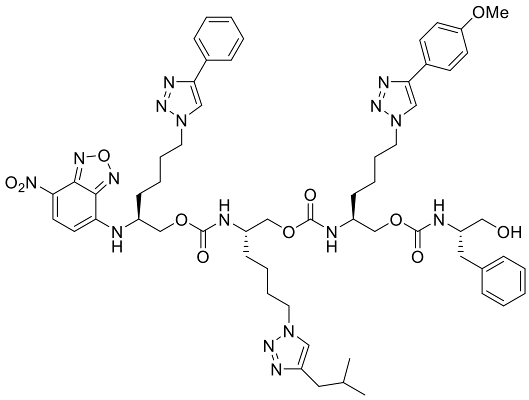

Exact Mass: 1182.57

***pdw**

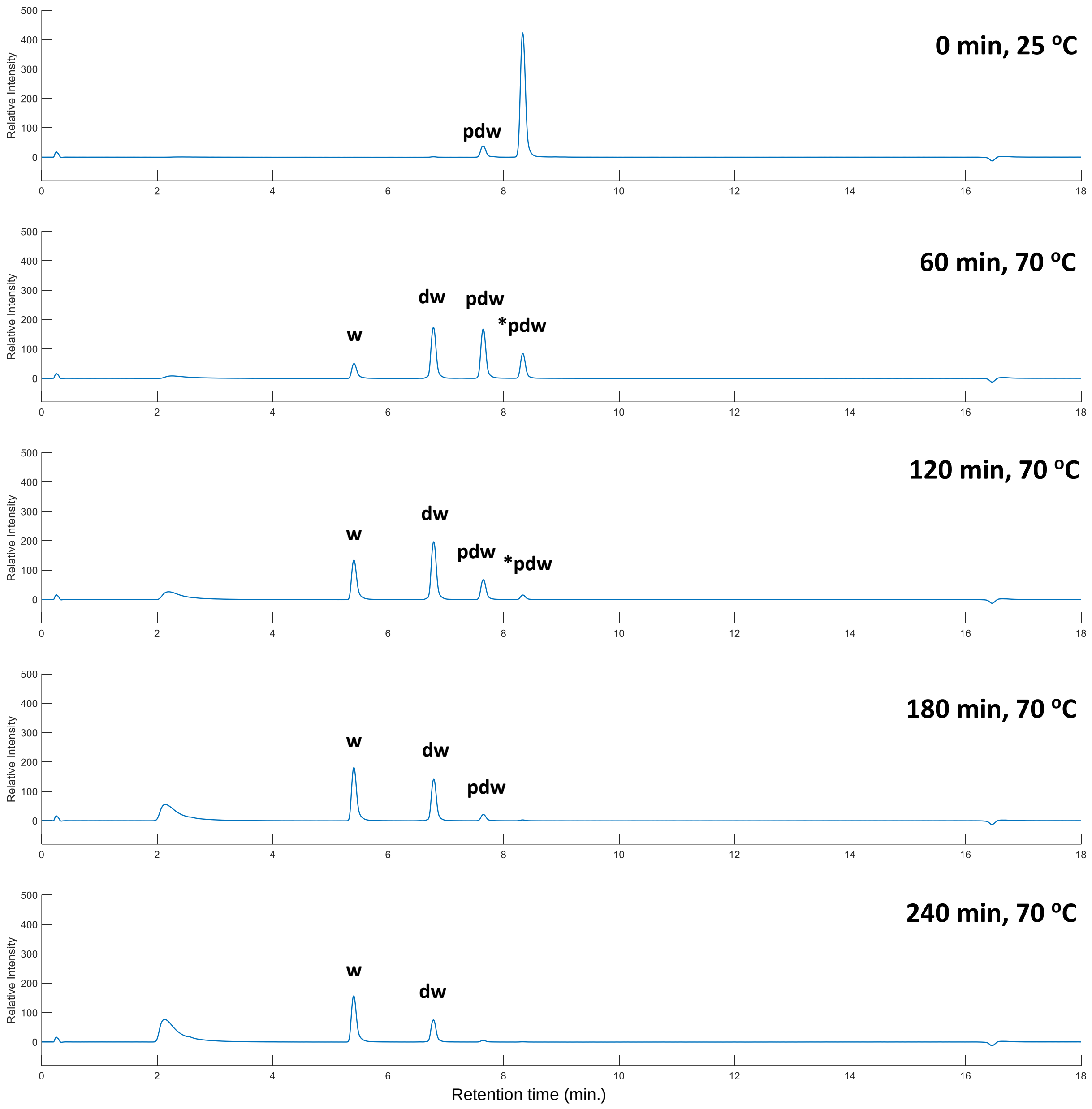

After 60 minutes of sequencing, all the following molecules are observed in the solution and analyzed via High-Res MS.

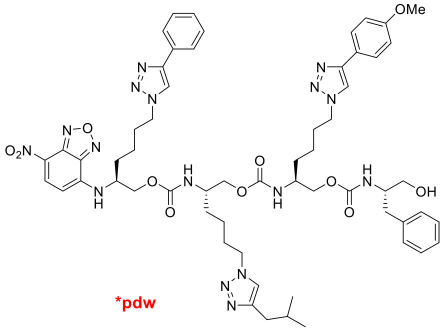

*pdw

Chemical Formula: $C_{59}H_{74}N_{16}O_{11}$
Exact Mass: 1182.57

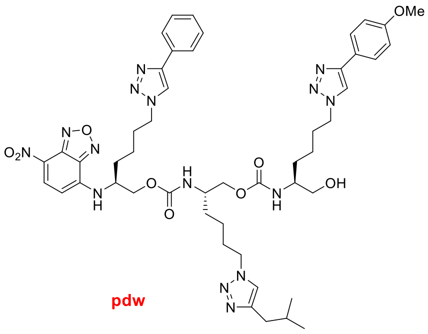

pdw

Chemical Formula: $C_{49}H_{63}N_{15}O_{9}$
Exact Mass: 1005.49

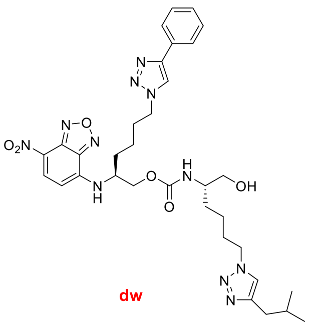

dw

Chemical Formula: $C_{33}H_{43}N_{11}O_{6}$
Exact Mass: 689.34

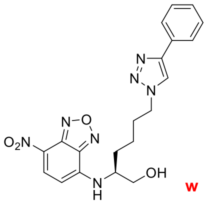

w

Chemical Formula: $C_{20}H_{21}N_{7}O_{4}$
Exact Mass: 423.17

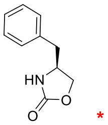

*

Chemical Formula: $C_{10}H_{11}NO_{2}$
Exact Mass: 177.08

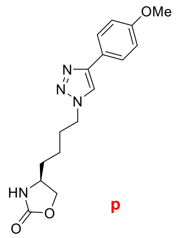

p

Chemical Formula: $C_{16}H_{20}N_{4}O_{3}$
Exact Mass: 316.15

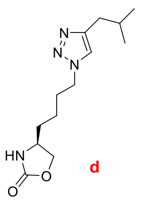

d

Chemical Formula: $C_{13}H_{22}N_{4}O_{2}$
Exact Mass: 266.17

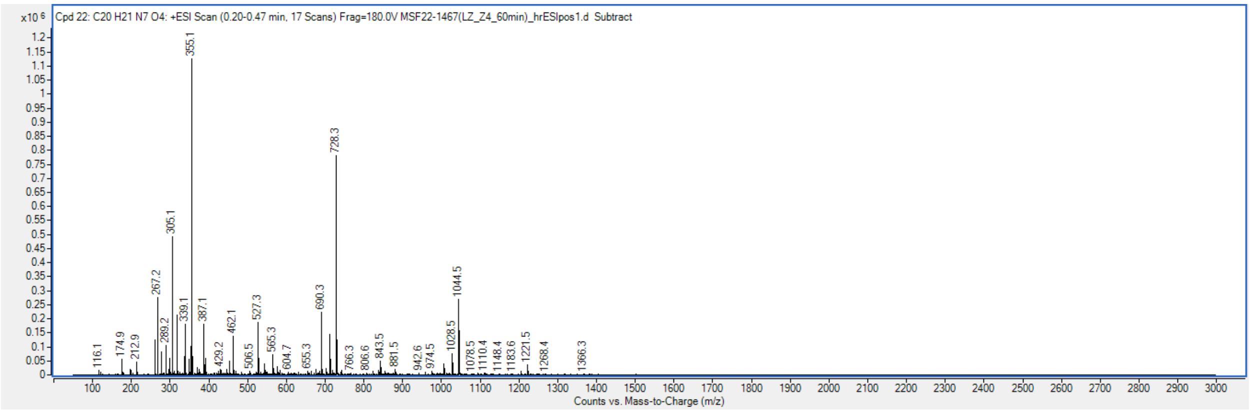

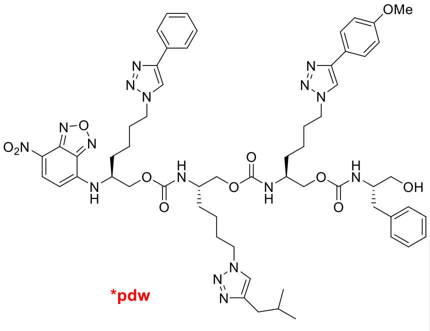

*pdw

Chemical Formula: $C_{59}H_{74}N_{16}O_{11}$
Exact Mass: 1182.57

MS Zoomed Spectrum

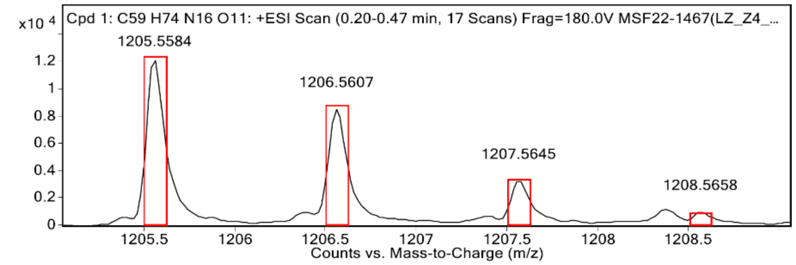

MS Spectrum Peak List

Obs. m/z	Calc. m/z	Charge	Abundance	Formula	Ion Species	Tgt Mass Error (ppm)
355.1165			1111499			
1205.5584	1205.5615	1	12185	C59H74N16O11	(M+Na)+	2.56
1206.5607	1206.5644	1	8579	C59H74N16O11	(M+Na)+	3.04
1207.5645	1207.5671	1	3323	C59H74N16O11	(M+Na)+	2.15
1208.5658	1208.5698	1	1046	C59H74N16O11	(M+Na)+	3.31
1209.6586	1209.5724	1	331	C59H74N16O11	(M+Na)+	-71.28

--- End Of Report ---

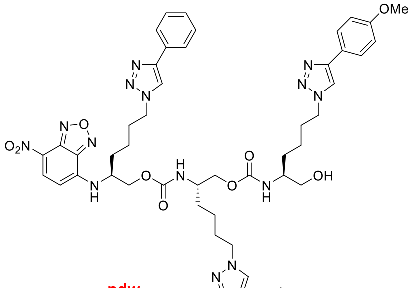

pdw

Chemical Formula: $C_{49}H_{63}N_{15}O_9$
Exact Mass: 1005.49

MS Zoomed Spectrum

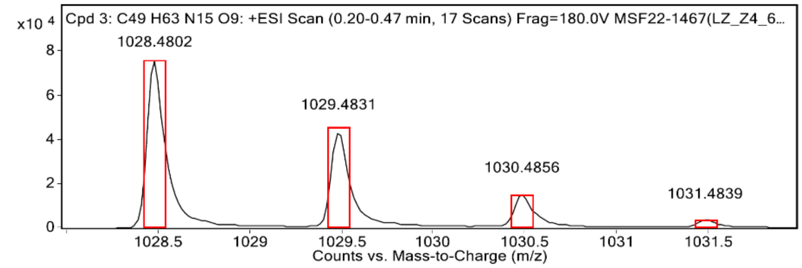

MS Spectrum Peak List

Obs. m/z	Calc. m/z	Charge	Abundance	Formula	Ion Species	Tgt Mass Error (ppm)
355.1165			1124680			
1028.4802	1028.4825	1	75801	C49H63N15O9	(M+Na)+	2.23
1029.4831	1029.4854	1	43996	C49H63N15O9	(M+Na)+	2.17
1030.4856	1030.4880	1	15425	C49H63N15O9	(M+Na)+	2.35
1031.4839	1031.4906	1	3558	C49H63N15O9	(M+Na)+	6.54

--- End Of Report ---

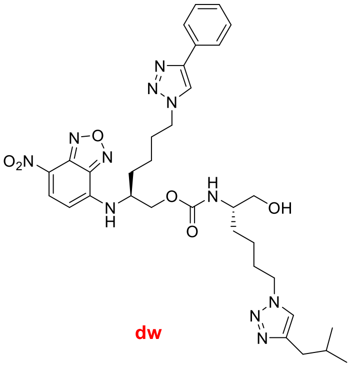

dw

Chemical Formula: $C_{33}H_{43}N_{11}O_6$
Exact Mass: 689.34

MS Zoomed Spectrum

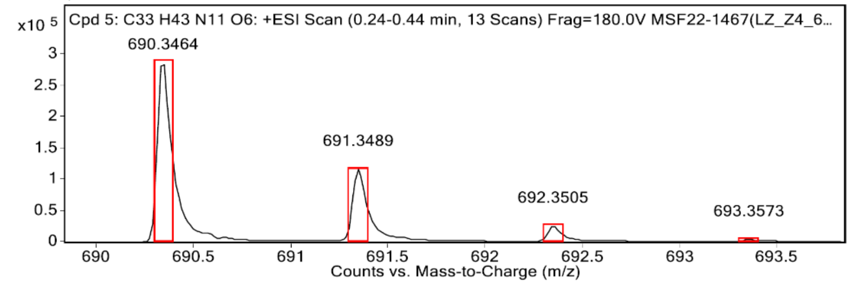

MS Spectrum Peak List

Obs. m/z	Calc. m/z	Charge	Abundance	Formula	Ion Species	Tgt Mass Error (ppm)
355.1164			1347538			
690.3464	690.3471	1	289473	C33H43N11O6	(M+H)+	0.92
691.3489	691.3498	1	116937	C33H43N11O6	(M+H)+	1.4
692.3505	692.3524	1	25994	C33H43N11O6	(M+H)+	2.7
693.3573	693.3549	1	4339	C33H43N11O6	(M+H)+	-3.5
694.4222	694.3574	1	1039	C33H43N11O6	(M+H)+	-93.42

--- End Of Report ---

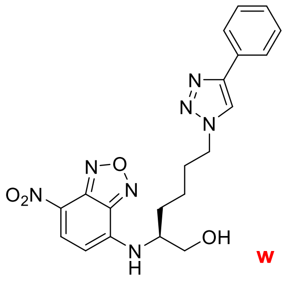

w

Chemical Formula: $C_{20}H_{21}N_7O_4$
Exact Mass: 423.17

MS Zoomed Spectrum

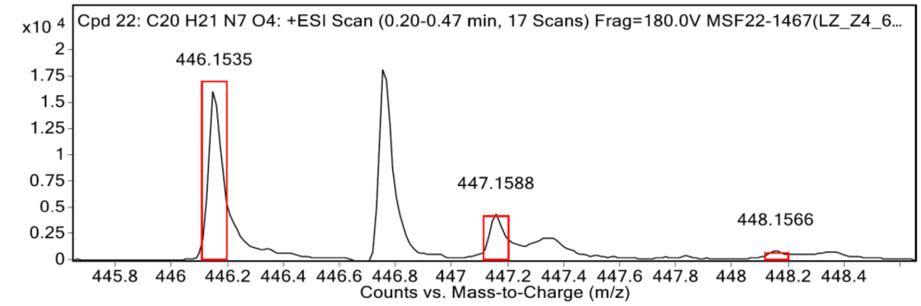

MS Spectrum Peak List

Obs. m/z	Calc. m/z	Charge	Abundance	Formula	Ion Species	Tgt Mass Error (ppm)
355.1165			1136338			
446.1535	446.1547	1	16223	C20H21N7O4	(M+Na)+	2.78
447.1588	447.1575	1	4480	C20H21N7O4	(M+Na)+	-2.93
448.1566	448.1599	1	978	C20H21N7O4	(M+Na)+	7.33

--- End Of Report ---

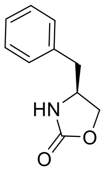

*

Chemical Formula: $C_{10}H_{11}NO_2$
Exact Mass: 177.08

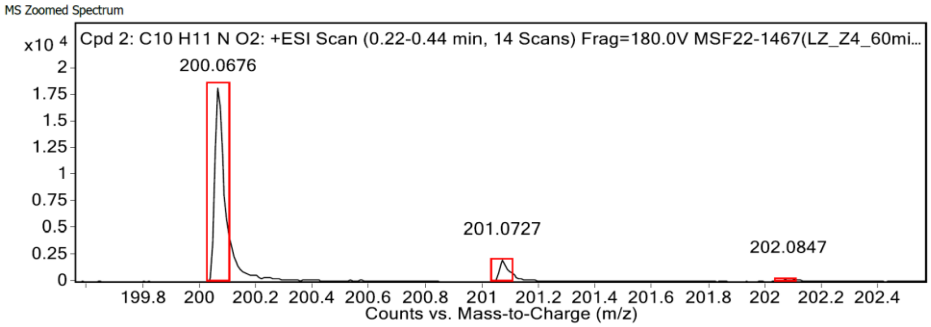

MS Spectrum Peak List

Obs. m/z	Calc. m/z	Charge	Abundance	Formula	Ion Species	Tgt Mass Error (ppm)
200.0676	200.0682	1	18515	C10H11NO2	(M+Na)+	2.84
201.0727	201.0714	1	2042	C10H11NO2	(M+Na)+	-6.72
202.0847	202.0737	1	328	C10H11NO2	(M+Na)+	-54.36
355.1164			1249394			

--- End Of Report ---

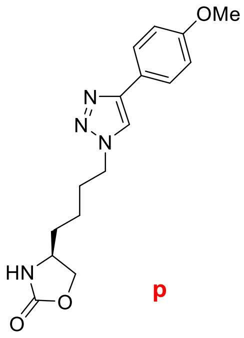

p

Chemical Formula: $C_{16}H_{20}N_4O_3$
Exact Mass: 316.15

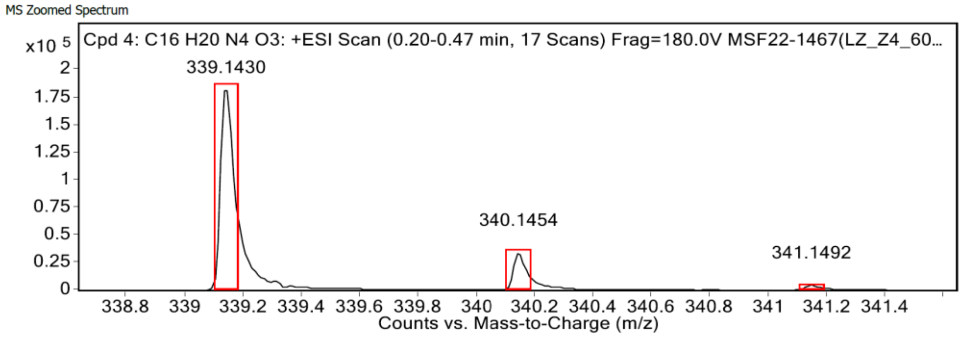

MS Spectrum Peak List

Obs. m/z	Calc. m/z	Charge	Abundance	Formula	Ion Species	Tgt Mass Error (ppm)
339.1430	339.1428	1	185836	C16H20N4O3	(M+Na)+	-0.63
340.1454	340.1457	1	34678	C16H20N4O3	(M+Na)+	0.74
341.1492	341.1481	1	5105	C16H20N4O3	(M+Na)+	-3.15
342.1561	342.1506	1	694	C16H20N4O3	(M+Na)+	-16.18
355.1165			1126090			

--- End Of Report ---

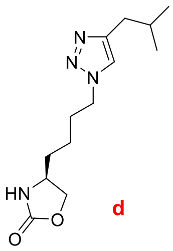

d

Chemical Formula: $C_{13}H_{22}N_4O_2$
Exact Mass: 266.17

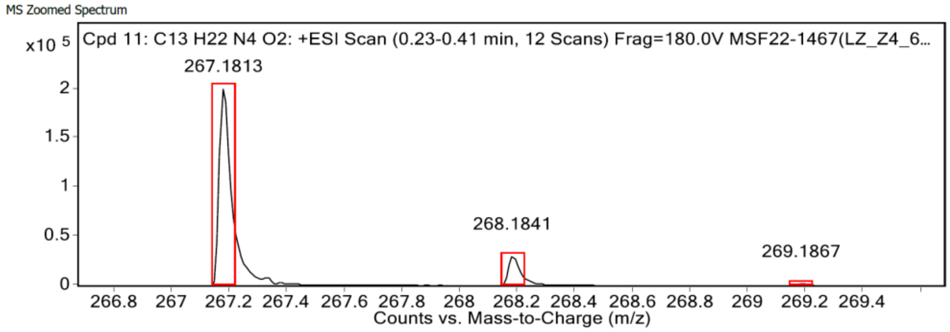

MS Spectrum Peak List

Obs. m/z	Calc. m/z	Charge	Abundance	Formula	Ion Species	Tgt Mass Error (ppm)
267.1813	267.1816	1	204025	C13H22N4O2	(M+H)+	0.81
268.1841	268.1844	1	30711	C13H22N4O2	(M+H)+	0.9
269.1867	269.1868	1	2803	C13H22N4O2	(M+H)+	0.55
270.1926	270.1892	1	295	C13H22N4O2	(M+H)+	-12.45
355.1164			2138946			

--- End Of Report ---

Oligomer Z5_1: *flvv

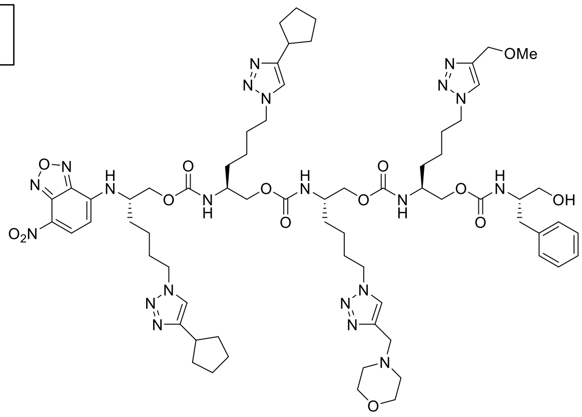

Exact Mass: 1433.77

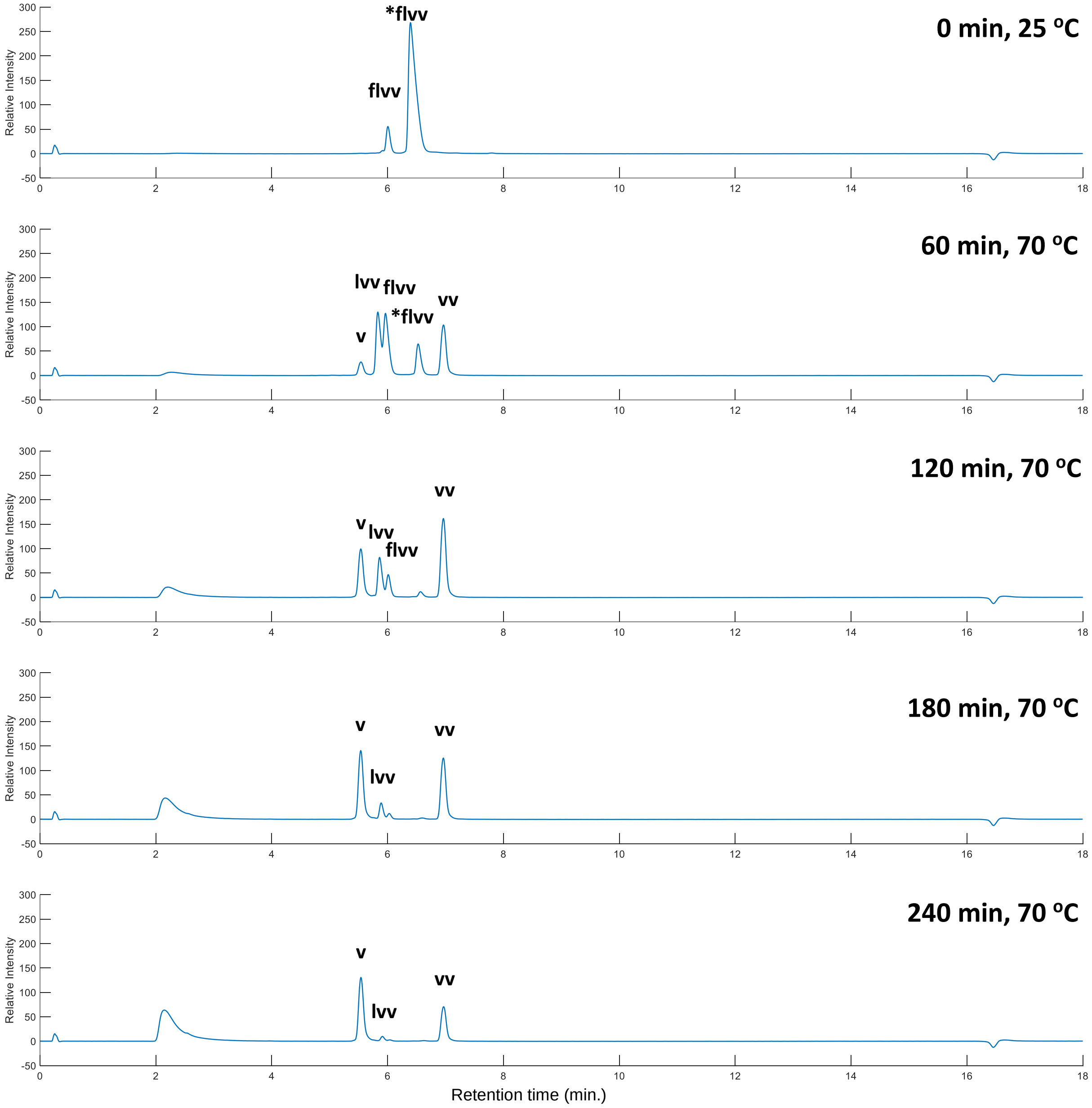

After 60 minutes of sequencing, all the following molecules are observed in the solution and analyzed via High-Res MS.

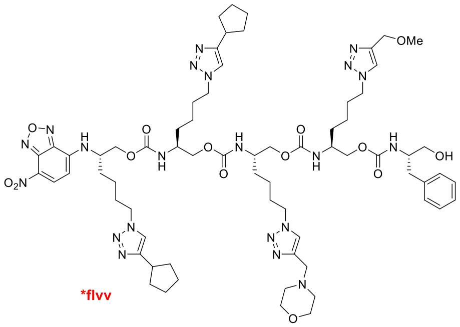

*flvv

Chemical Formula: $C_{68}H_{99}N_{21}O_{14}$
Exact Mass: 1433.77

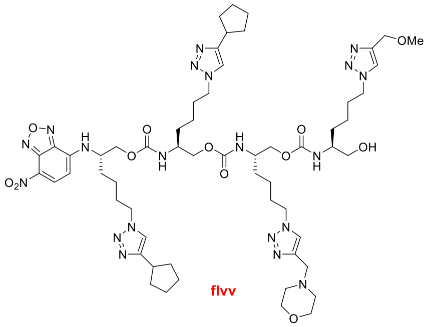

flvv

Chemical Formula: $C_{58}H_{88}N_{20}O_{12}$
Exact Mass: 1256.69

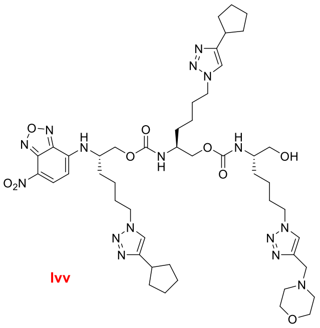

lvv

Chemical Formula: $C_{47}H_{70}N_{16}O_{9}$
Exact Mass: 1002.55

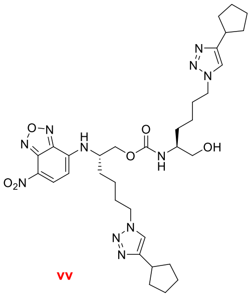

vv

Chemical Formula: $C_{33}H_{47}N_{11}O_{6}$
Exact Mass: 693.37

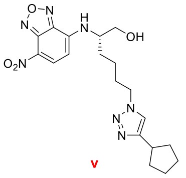

v

Chemical Formula: $C_{19}H_{25}N_{7}O_{4}$
Exact Mass: 415.20

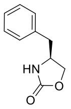

*

Chemical Formula: $C_{10}H_{11}NO_{2}$
Exact Mass: 177.08

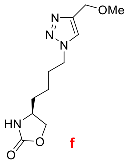

f

Chemical Formula: $C_{11}H_{18}N_{4}O_{3}$
Exact Mass: 254.14

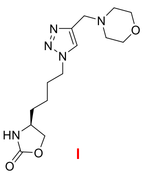

l

Chemical Formula: $C_{14}H_{23}N_{5}O_{3}$
Exact Mass: 309.18

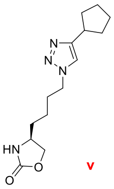

v

Chemical Formula: $C_{14}H_{22}N_{4}O_{2}$
Exact Mass: 278.17

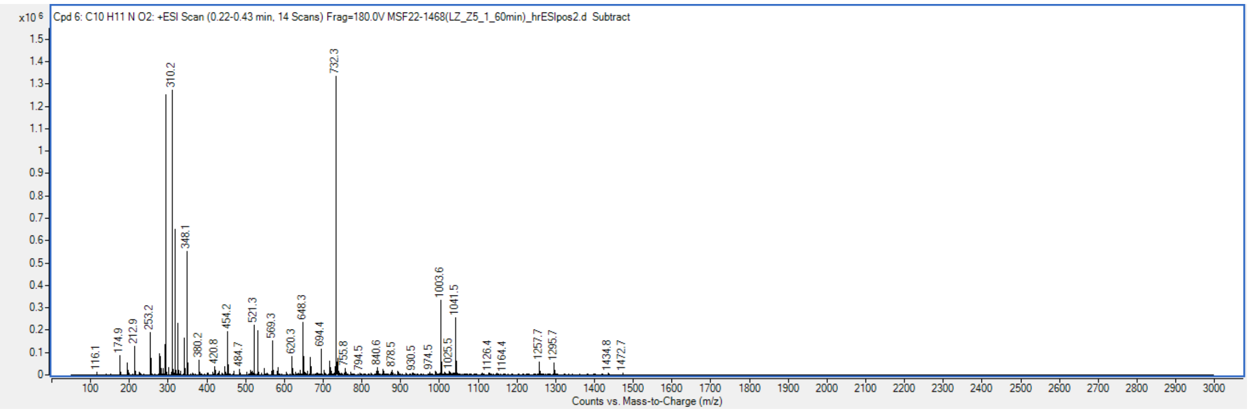

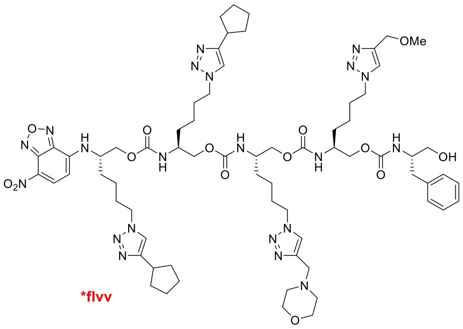

*flvv

Chemical Formula: $C_{68}H_{99}N_{21}O_{14}$
Exact Mass: 1433.77

MS Zoomed Spectrum

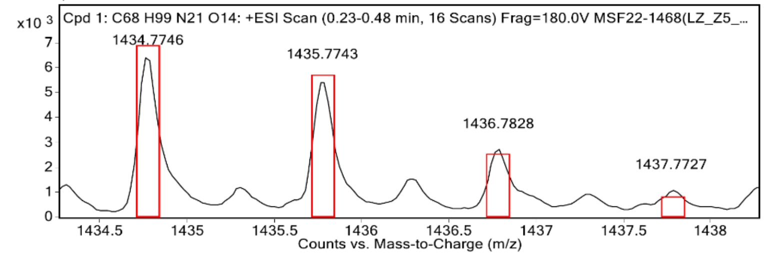

MS Spectrum Peak List

Obs. m/z	Calc. m/z	Charge	Abundance	Formula	Ion Species	Tgt Mass Error (ppm)
310.1871			1319422			
1434.7746	1434.7753	1	6507	C68H99N21O14	(M+H)+	0.52
1435.7743	1435.7781	1	5558	C68H99N21O14	(M+H)+	2.68
1436.7828	1436.7808	1	2759	C68H99N21O14	(M+H)+	-1.34
1437.7727	1437.7835	1	1119	C68H99N21O14	(M+H)+	7.51

--- End Of Report ---

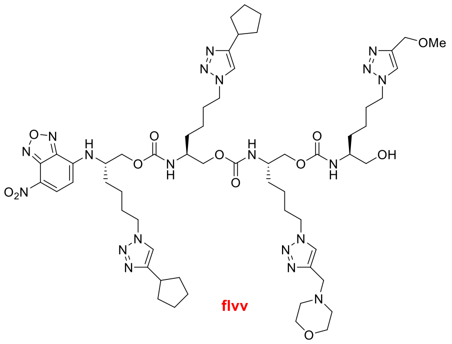

flvv

Chemical Formula: $C_{58}H_{88}N_{20}O_{12}$
Exact Mass: [illegible]

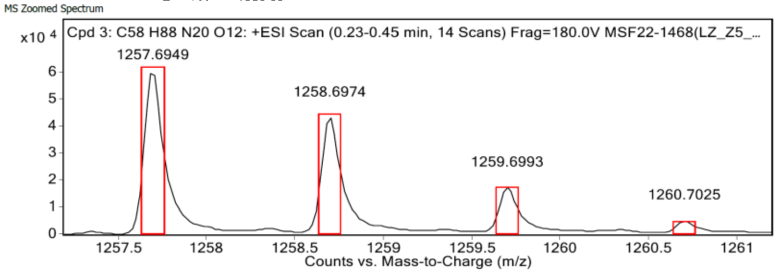

MS Spectrum Peak List

Obs. m/z	Calc. m/z	Charge	Abundance	Formula	Ion Species	Tgt Mass Error (ppm)
732.3339			1417106			
1257.6949	1257.6963	1	60978	C58H88N20O12	(M+H)+	1.15
1258.6974	1258.6991	1	43406	C58H88N20O12	(M+H)+	1.37
1259.6993	1259.7017	1	17361	C58H88N20O12	(M+H)+	1.89
1260.7025	1260.7043	1	5165	C58H88N20O12	(M+H)+	1.45
1261.6976	1261.7068	1	1822	C58H88N20O12	(M+H)+	7.32

--- End Of Report ---

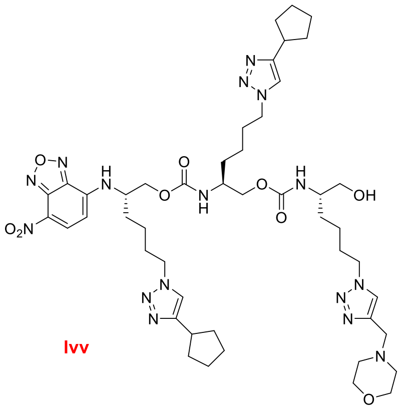

lvv

Chemical Formula: $C_{47}H_{70}N_{16}O_{9}$
Exact Mass: 1002.55

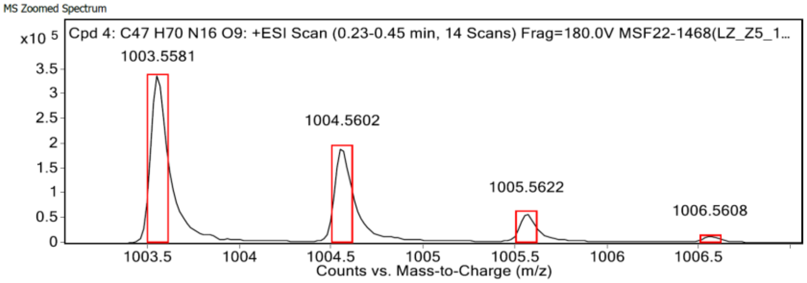

MS Spectrum Peak List

Obs. m/z	Calc. m/z	Charge	Abundance	Formula	Ion Species	Tgt Mass Error (ppm)
732.3339			1417495			
1003.5581	1003.5584	1	339733	C47H70N16O9	(M+H)+	0.34
1004.5602	1004.5612	1	193654	C47H70N16O9	(M+H)+	0.96
1005.5622	1005.5638	1	58838	C47H70N16O9	(M+H)+	1.61
1006.5608	1006.5664	1	11576	C47H70N16O9	(M+H)+	5.53
1007.5708	1007.5689	1	2325	C47H70N16O9	(M+H)+	-1.89
1008.6315	1008.5713	1	351	C47H70N16O9	(M+H)+	-59.63

--- End Of Report ---

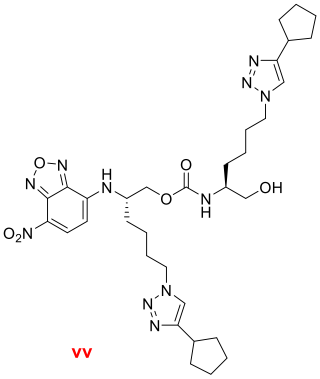

vv

Chemical Formula: $C_{33}H_{47}N_{11}O_6$
Exact Mass: 693.37

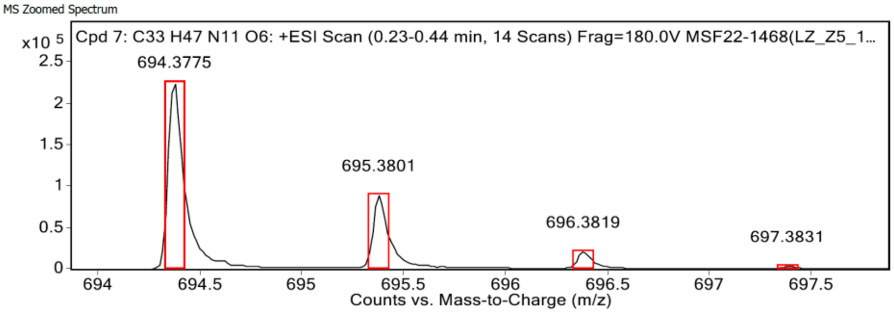

MS Spectrum Peak List

Obs. m/z	Calc. m/z	Charge	Abundance	Formula	Ion Species	Tgt Mass Error (ppm)
310.1870			1466951			
694.3775	694.3784	1	225503	C33H47N11O6	(M+H)+	1.23
695.3801	695.3811	1	89447	C33H47N11O6	(M+H)+	1.55
696.3819	696.3837	1	21247	C33H47N11O6	(M+H)+	2.63
697.3831	697.3862	1	3503	C33H47N11O6	(M+H)+	4.52
698.3601	698.3887	1	826	C33H47N11O6	(M+H)+	40.92

--- End Of Report ---

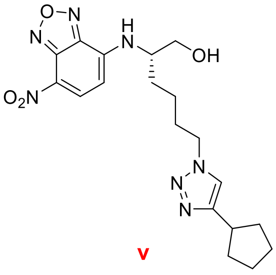

v

Chemical Formula: $C_{19}H_{25}N_7O_4$
Exact Mass: 415.20

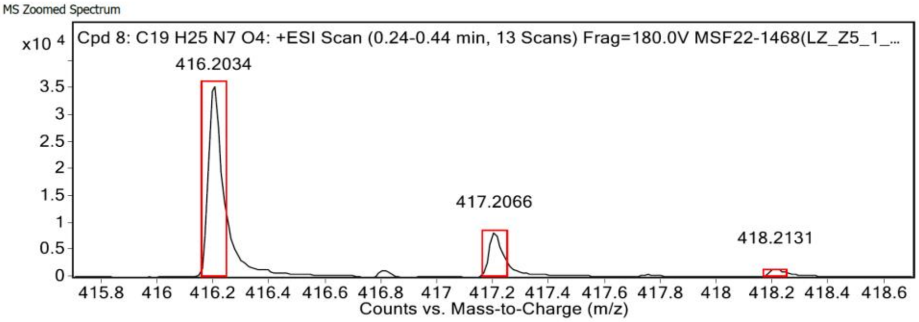

MS Spectrum Peak List

Obs. m/z	Calc. m/z	Charge	Abundance	Formula	Ion Species	Tgt Mass Error (ppm)
310.1870			1579303			
416.2034	416.2041	1	35990	C19H25N7O4	(M+H)+	1.74
417.2066	417.2068	1	8355	C19H25N7O4	(M+H)+	0.35
418.2131	418.2092	1	1581	C19H25N7O4	(M+H)+	-9.4

--- End Of Report ---

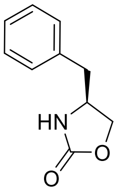

*

Chemical Formula:
$C_{10}H_{11}NO_2$
Exact Mass: 177.08

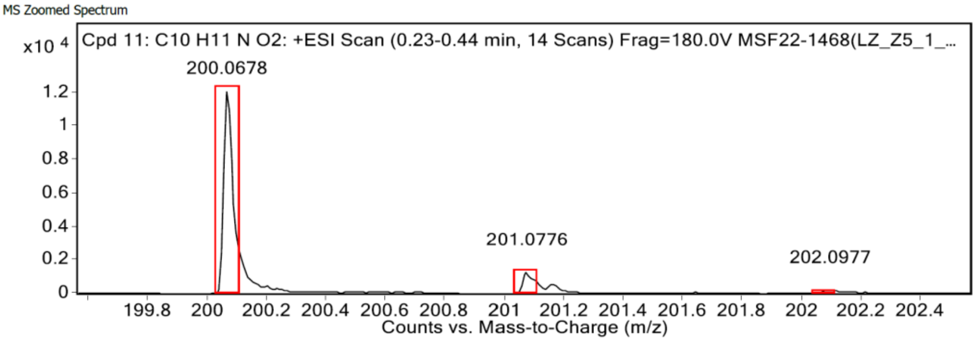

MS Spectrum Peak List

Obs. m/z	Calc. m/z	Charge	Abundance	Formula	Ion Species	Tgt Mass Error (ppm)
200.0678	200.0682	1	12276	C10H11NO2	(M+Na)+	1.99
201.0776	201.0714	1	1312	C10H11NO2	(M+Na)+	-30.94
202.0977	202.0737	1	245	C10H11NO2	(M+Na)+	-118.93
310.1870			1466891			

--- End Of Report ---

MS Zoomed Spectrum

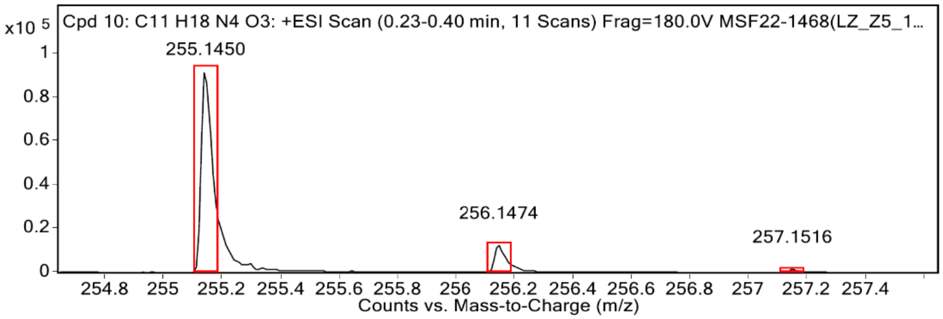

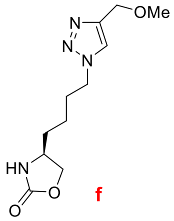

f

Chemical Formula: $C_{11}H_{18}N_4O_3$
Exact Mass: 254.14

MS Spectrum Peak List

Obs. m/z	Calc. m/z	Charge	Abundance	Formula	Ion Species	Tgt Mass Error (ppm)
255.1450	255.1452	1	93982	C11H18N4O3	(M+H)+	0.59
256.1474	256.1479	1	12553	C11H18N4O3	(M+H)+	1.83
257.1516	257.1501	1	1635	C11H18N4O3	(M+H)+	-5.79
258.1549	258.1524	1	296	C11H18N4O3	(M+H)+	-9.36
310.1871			1547083			

--- End Of Report ---

MS Zoomed Spectrum

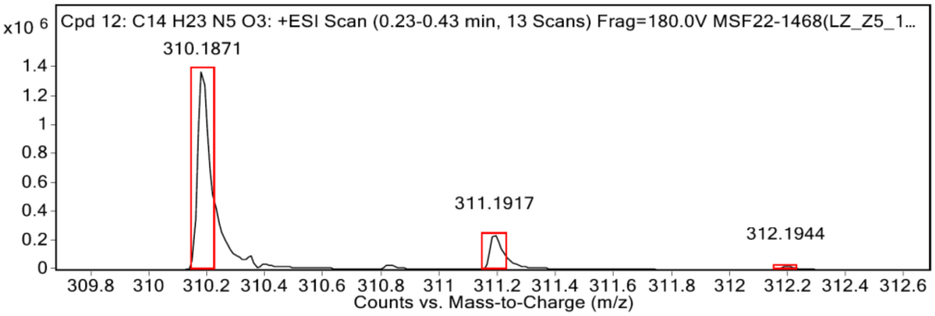

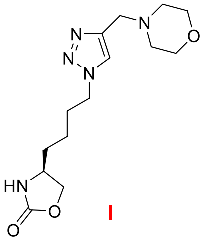

l

Chemical Formula: $C_{14}H_{23}N_5O_3$
Exact Mass: 309.18

MS Spectrum Peak List

Obs. m/z	Calc. m/z	Charge	Abundance	Formula	Ion Species	Tgt Mass Error (ppm)
310.1871	310.1874	1	1395079	C14H23N5O3	(M+H)+	0.79
311.1917	311.1901	1	242452	C14H23N5O3	(M+H)+	-5
312.1944	312.1924	1	28381	C14H23N5O3	(M+H)+	-6.15
313.2042	313.1948	1	2587	C14H23N5O3	(M+H)+	-29.88
732.3339			1437123			

--- End Of Report ---

MS Zoomed Spectrum

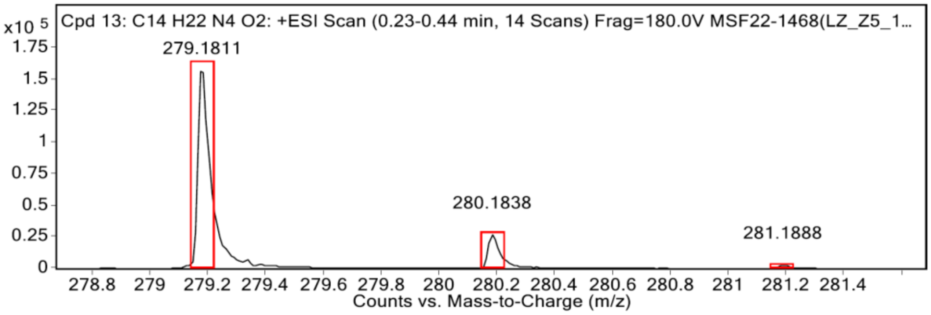

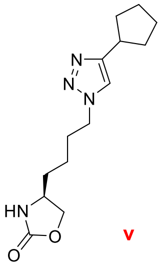

v

Chemical Formula: $C_{14}H_{22}N_4O_2$
Exact Mass: 278.17

MS Spectrum Peak List

Obs. m/z	Calc. m/z	Charge	Abundance	Formula	Ion Species	Tgt Mass Error (ppm)
279.1811	279.1816	1	163805	C14H22N4O2	(M+H)+	1.59
280.1838	280.1844	1	27120	C14H22N4O2	(M+H)+	2.32
281.1888	281.1869	1	3208	C14H22N4O2	(M+H)+	-6.59
310.1870			1466986			

--- End Of Report ---

Oligomer Z5_2: *flvv

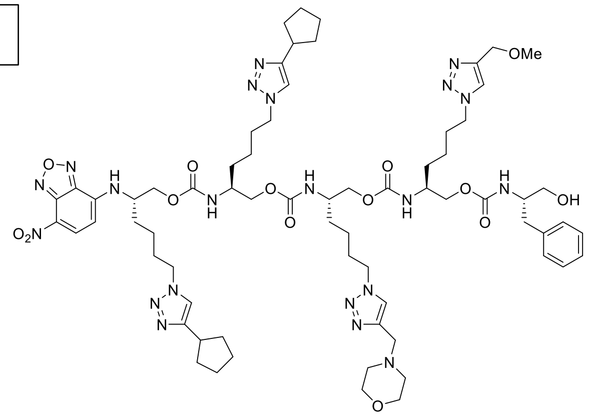

Exact Mass: 1433.77

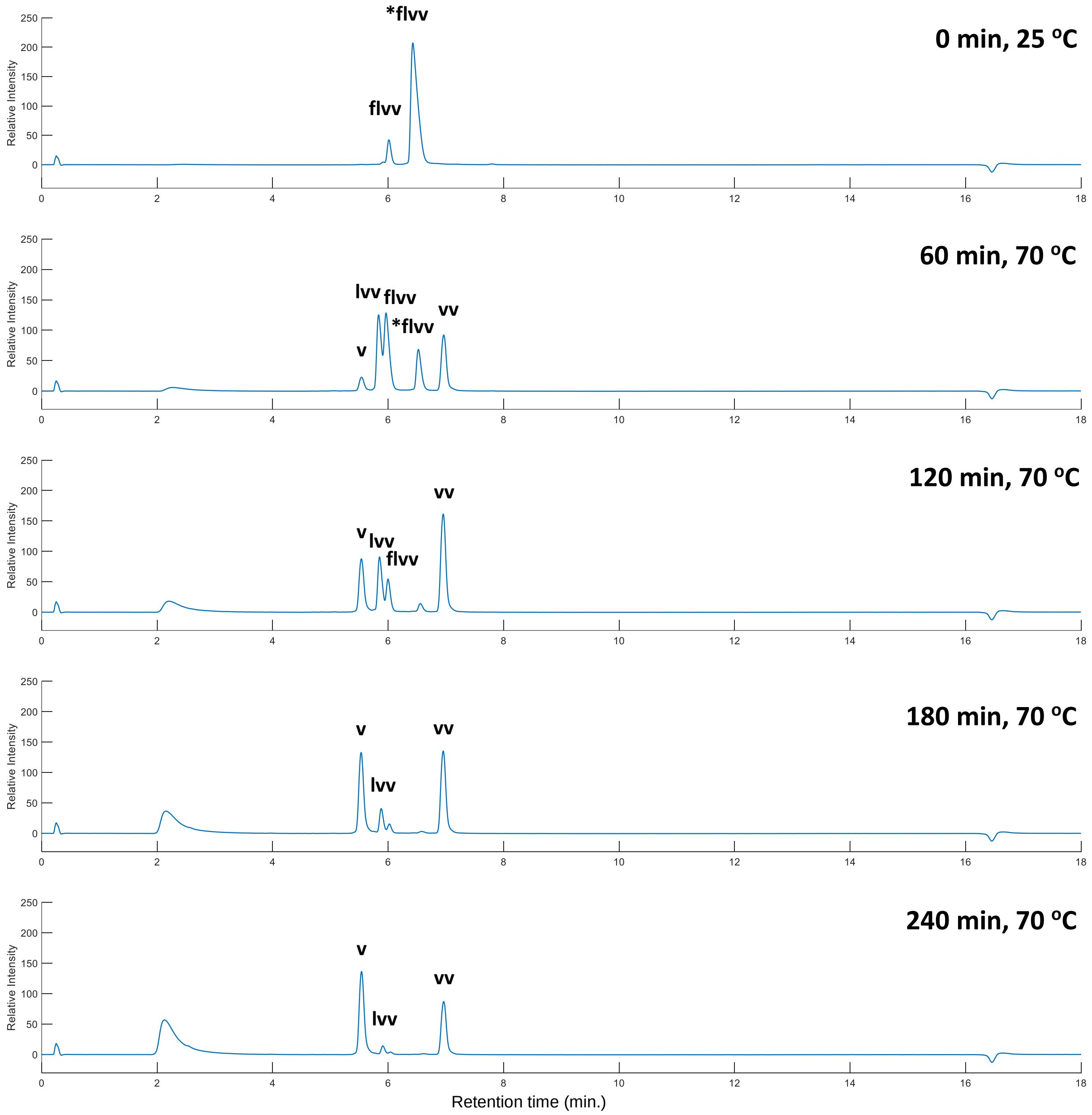

Oligomer Z6_1: *4e5f

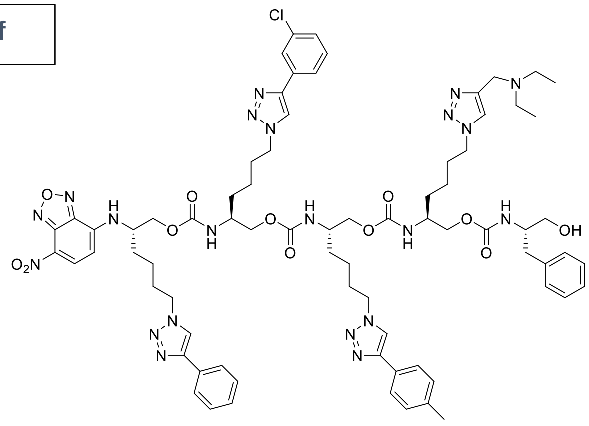

Exact Mass: 1515.71

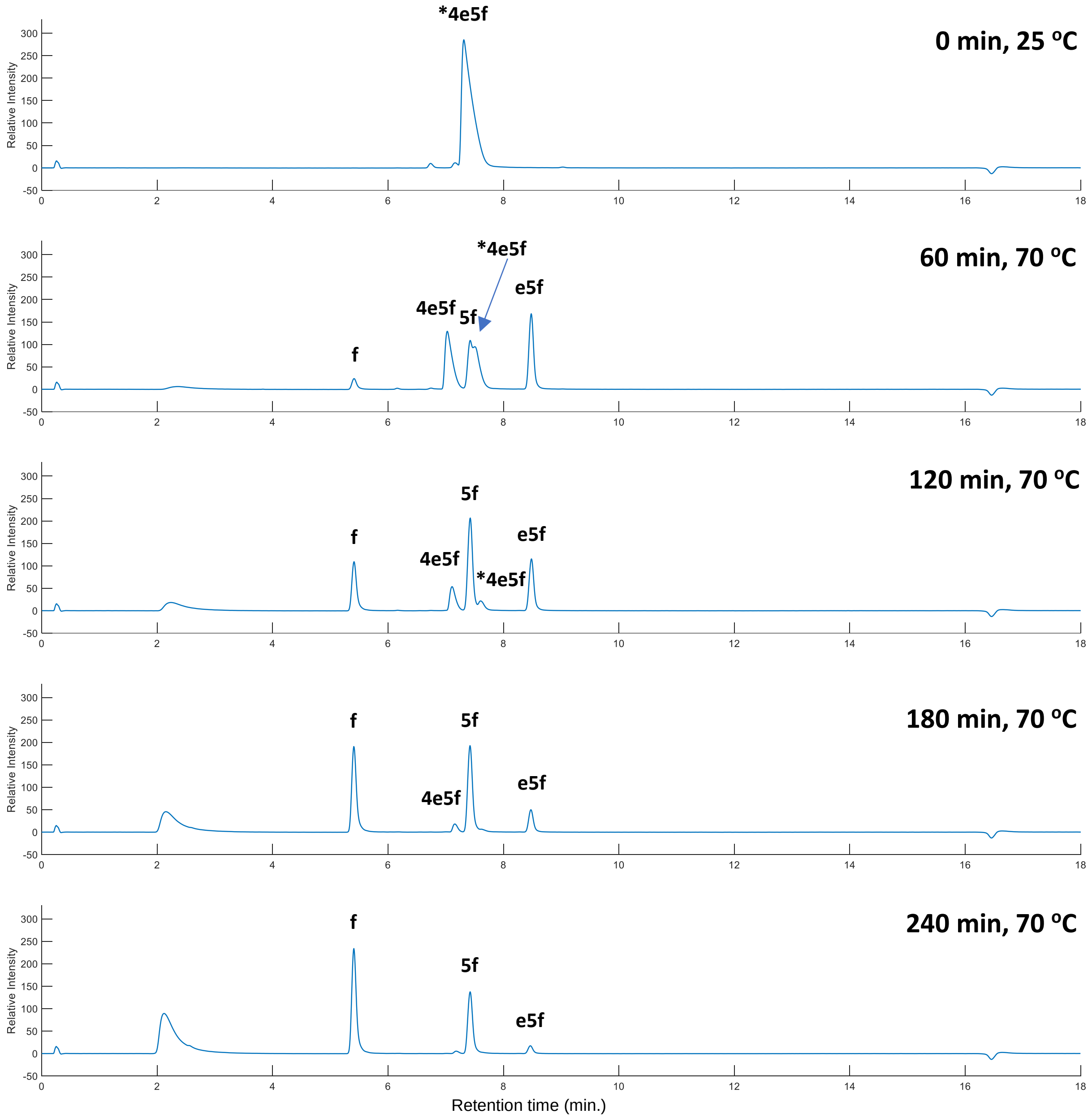

After 60 minutes of sequencing, all the following molecules are observed in the solution and analyzed via High-Res MS.

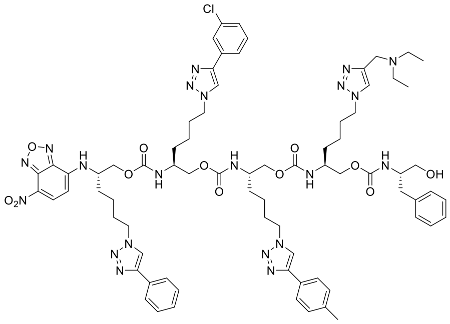

***4e5f** Chemical Formula: $C_{75}H_{94}ClN_{21}O_{12}$
Exact Mass: 1515.71

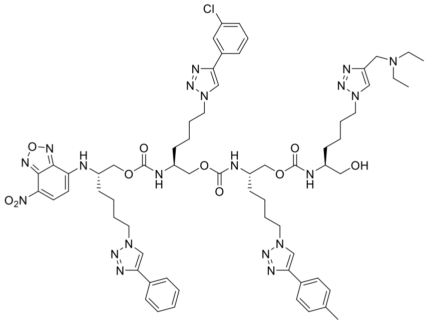

4e5f Chemical Formula: $C_{65}H_{83}ClN_{20}O_{10}$
Exact Mass: 1338.63

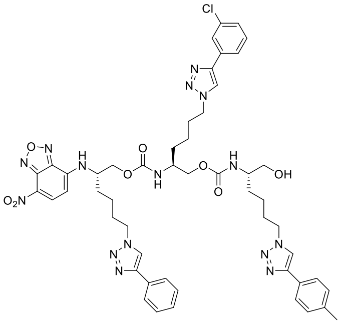

e5f Chemical Formula: $C_{51}H_{58}ClN_{15}O_{8}$
Exact Mass: 1043.43

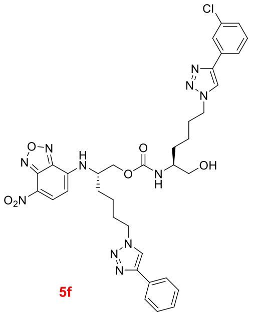

5f

Chemical Formula: $C_{35}H_{38}ClN_{11}O_{6}$
Exact Mass: 743.27

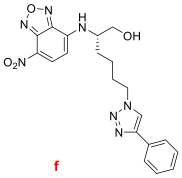

f

Chemical Formula: $C_{20}H_{21}N_{7}O_{4}$
Exact Mass: 423.17

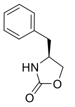

Chemical Formula: $C_{10}H_{11}NO_{2}$
Exact Mass: 177.08
*

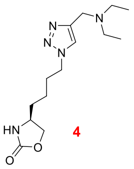

4

Chemical Formula: $C_{14}H_{25}N_{5}O_{2}$
Exact Mass: 295.20

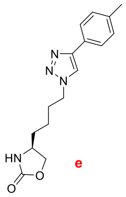

e

Chemical Formula: $C_{16}H_{20}N_{4}O_{2}$
Exact Mass: 300.16

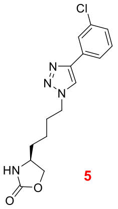

5

Chemical Formula: $C_{15}H_{17}ClN_{4}O_{2}$
Exact Mass: 320.10

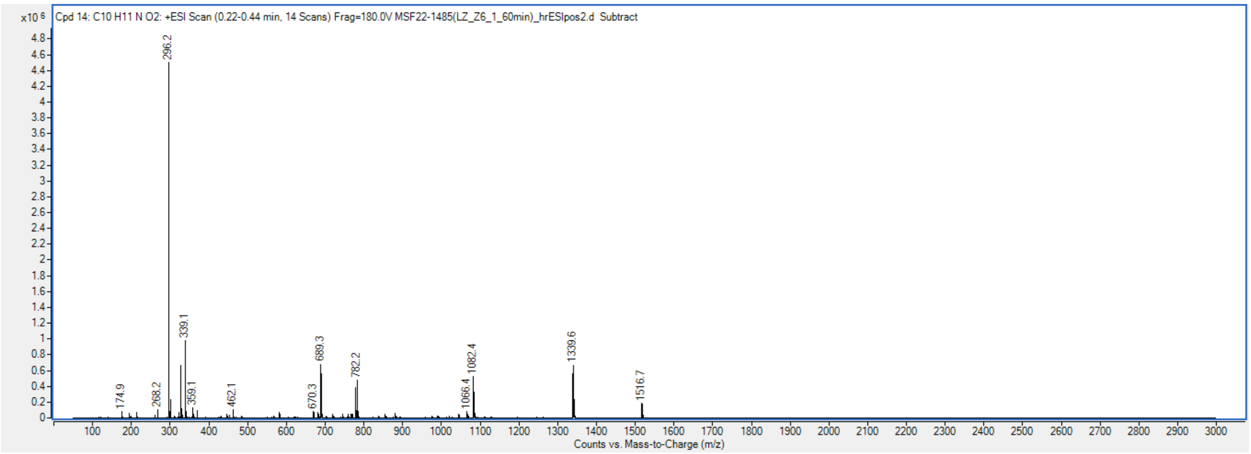

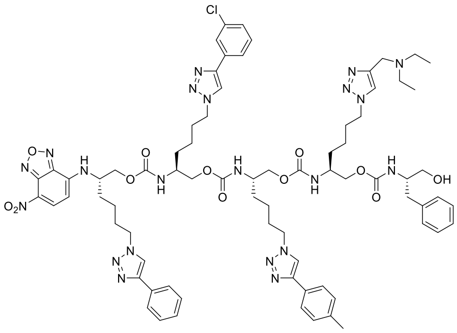

***4e5f** Chemical Formula: $C_{75}H_{94}ClN_{21}O_{12}$
Exact Mass: 1515.71

MS Zoomed Spectrum

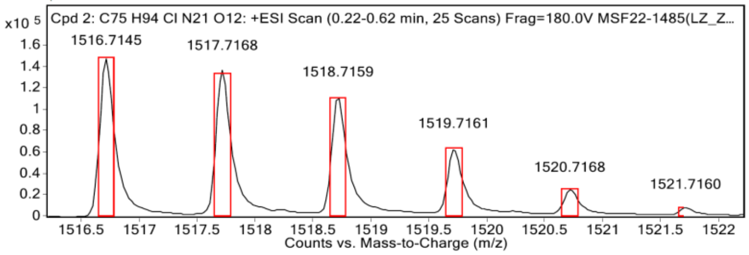

MS Spectrum Peak List

Obs. m/z	Calc. m/z	Charge	Abundance	Formula	Ion Species	Tgt Mass Error (ppm)
296.2077			4027732			
1516.7145	1516.7152	1	147801	C75H94ClN21O12	(M+H)+	0.47
1517.7168	1517.7181	1	136649	C75H94ClN21O12	(M+H)+	0.84
1518.7159	1518.7172	1	111863	C75H94ClN21O12	(M+H)+	0.83
1519.7161	1519.7179	1	63748	C75H94ClN21O12	(M+H)+	1.2
1520.7168	1520.7196	1	22808	C75H94ClN21O12	(M+H)+	1.86
1521.7160	1521.7218	1	6843	C75H94ClN21O12	(M+H)+	3.76
1522.7186	1522.7241	1	1843	C75H94ClN21O12	(M+H)+	3.62
1523.6572	1523.7265	1	550	C75H94ClN21O12	(M+H)+	45.5

--- End Of Report ---

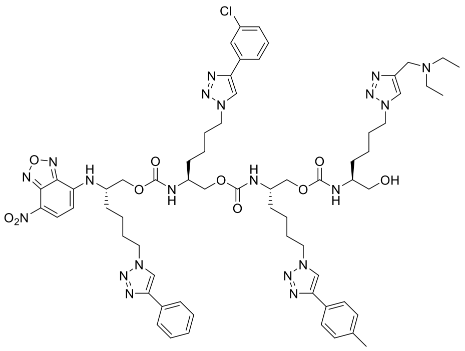

4e5f Chemical Formula: $C_{65}H_{83}ClN_{20}O_{10}$
Exact Mass: 1338.63

MS Zoomed Spectrum

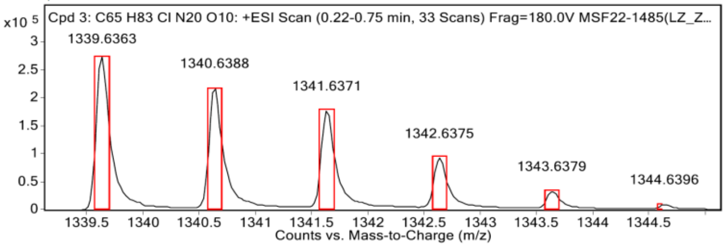

MS Spectrum Peak List

Obs. m/z	Calc. m/z	Charge	Abundance	Formula	Ion Species	Tgt Mass Error (ppm)
296.2077			2549357			
1339.6363	1339.6362	1	274774	C65H83ClN20O10	(M+H)+	-0.06
1340.6388	1340.6390	1	218795	C65H83ClN20O10	(M+H)+	0.16
1341.6371	1341.6376	1	178187	C65H83ClN20O10	(M+H)+	0.36
1342.6375	1342.6384	1	93068	C65H83ClN20O10	(M+H)+	0.66
1343.6379	1343.6402	1	34085	C65H83ClN20O10	(M+H)+	1.69
1344.6396	1344.6424	1	10338	C65H83ClN20O10	(M+H)+	2.12
1345.6400	1345.6447	1	3456	C65H83ClN20O10	(M+H)+	3.52

--- End Of Report ---

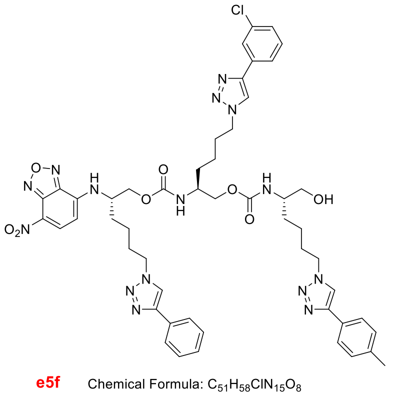

e5f Chemical Formula: $C_{51}H_{58}ClN_{15}O_8$
Exact Mass: 1043.43

MS Zoomed Spectrum

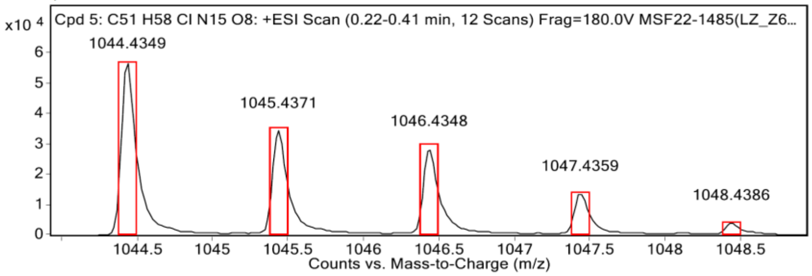

MS Spectrum Peak List

Obs. m/z	Calc. m/z	Charge	Abundance	Formula	Ion Species	Tgt Mass Error (ppm)
296.2077			4116022			
1044.4349	1044.4354	1	57102	C51H58ClN15O8	(M+H)+	0.47
1045.4371	1045.4382	1	34677	C51H58ClN15O8	(M+H)+	1.08
1046.4348	1046.4358	1	28709	C51H58ClN15O8	(M+H)+	0.93
1047.4359	1047.4369	1	13870	C51H58ClN15O8	(M+H)+	0.92
1048.4386	1048.4389	1	4234	C51H58ClN15O8	(M+H)+	0.37
1049.4423	1049.4413	1	1288	C51H58ClN15O8	(M+H)+	-1.03

--- End Of Report ---

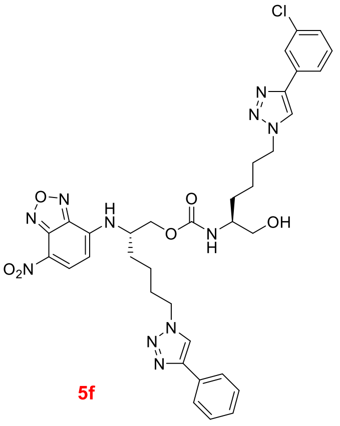

5f

Chemical Formula: $C_{35}H_{38}ClN_{11}O_6$
Exact Mass: 743.27

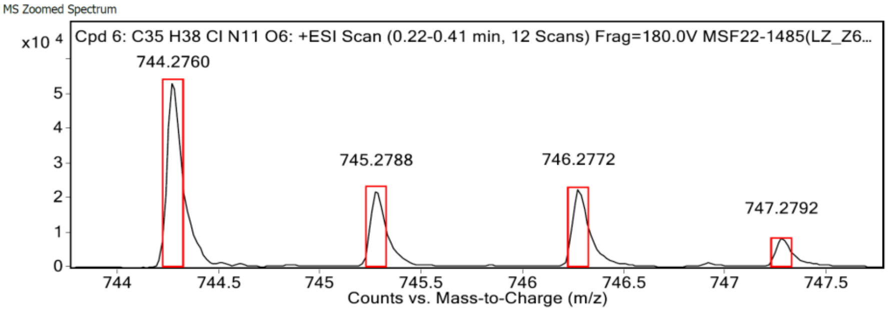

MS Spectrum Peak List

Obs. m/z	Calc. m/z	Charge	Abundance	Formula	Ion Species	Tgt Mass Error (ppm)
296.2077			4130249			
744.2760	744.2768	1	54173	C35H38ClN11O6	(M+H)+	1.12
745.2788	745.2796	1	22515	C35H38ClN11O6	(M+H)+	1.02
746.2772	746.2758	1	22706	C35H38ClN11O6	(M+H)+	-1.78
747.2792	747.2775	1	8634	C35H38ClN11O6	(M+H)+	-2.19
748.2913	748.2798	1	2091	C35H38ClN11O6	(M+H)+	-15.44

--- End Of Report ---

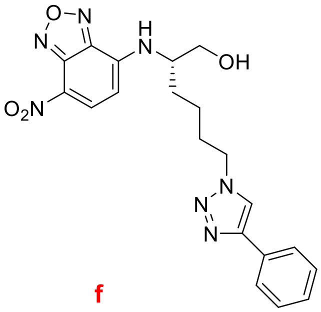

f

Chemical Formula: $C_{20}H_{21}N_7O_4$
Exact Mass: 423.17

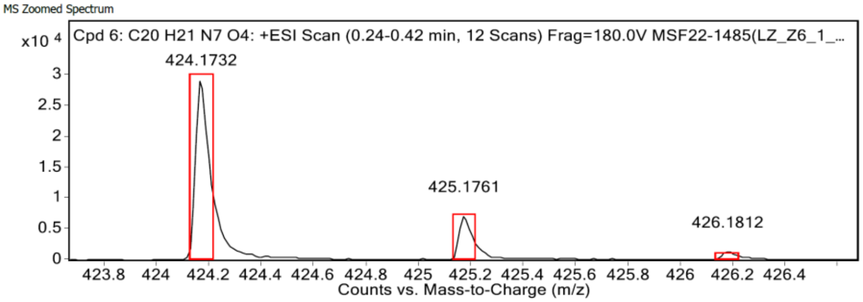

MS Spectrum Peak List

Obs. m/z	Calc. m/z	Charge	Abundance	Formula	Ion Species	Tgt Mass Error (ppm)
296.2074			2614340			
424.1732	424.1728	1	29717	C20H21N7O4	(M+H)+	-1.04
425.1761	425.1755	1	7217	C20H21N7O4	(M+H)+	-1.28
426.1812	426.1780	1	1448	C20H21N7O4	(M+H)+	-7.56
427.2155	427.1803	1	210	C20H21N7O4	(M+H)+	-82.23

--- End Of Report ---

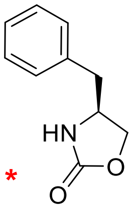

*

Chemical Formula: $C_{10}H_{11}NO_2$
Exact Mass: 177.08

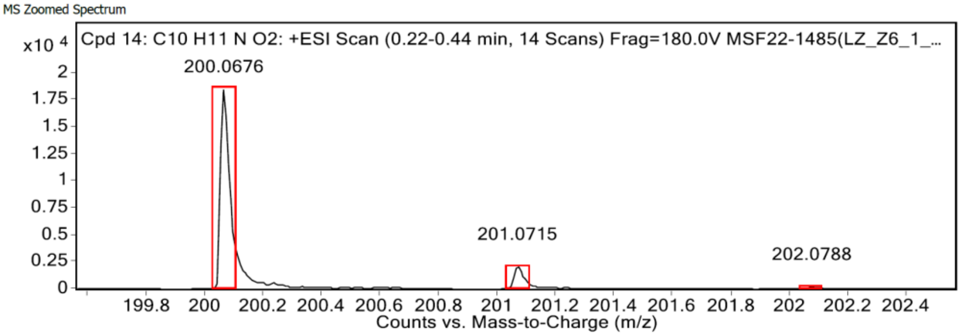

MS Spectrum Peak List

Obs. m/z	Calc. m/z	Charge	Abundance	Formula	Ion Species	Tgt Mass Error (ppm)
200.0676	200.0682	1	18541	C10H11NO2	(M+Na)+	3
201.0715	201.0714	1	2145	C10H11NO2	(M+Na)+	-0.5
202.0788	202.0737	1	287	C10H11NO2	(M+Na)+	-25.33
296.2077			4499905			

--- End Of Report ---

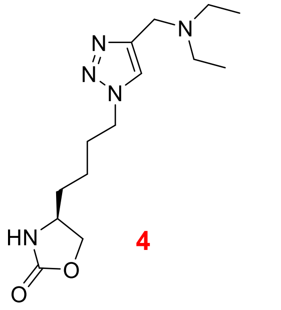

4

Chemical Formula: $C_{14}H_{25}N_5O_2$
Exact Mass: 295.20

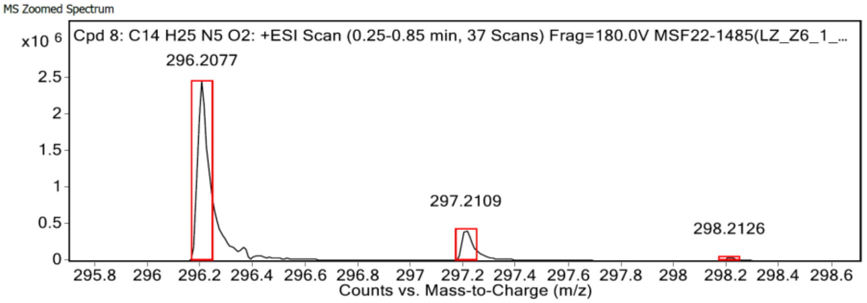

MS Spectrum Peak List

Obs. m/z	Calc. m/z	Charge	Abundance	Formula	Ion Species	Tgt Mass Error (ppm)
296.2077	296.2081	1	2459419	C14H25N5O2	(M+H)+	1.26
297.2109	297.2108	1	422901	C14H25N5O2	(M+H)+	-0.18
298.2126	298.2133	1	44286	C14H25N5O2	(M+H)+	2.37
299.2148	299.2156	1	3356	C14H25N5O2	(M+H)+	2.67

--- End Of Report ---

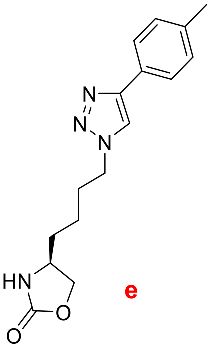

e

Chemical Formula: $C_{16}H_{20}N_4O_2$
Exact Mass: 300.16

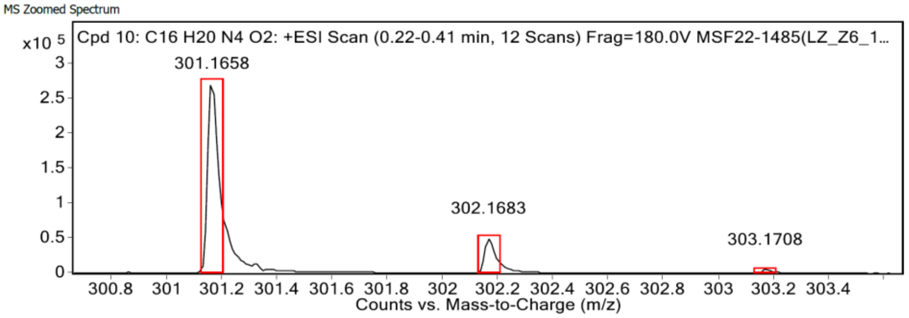

MS Spectrum Peak List

Obs. m/z	Calc. m/z	Charge	Abundance	Formula	Ion Species	Tgt Mass Error (ppm)
296.2077			4192038			
301.1658	301.1659	1	276416	C16H20N4O2	(M+H)+	0.23
302.1683	302.1688	1	50363	C16H20N4O2	(M+H)+	1.67
303.1708	303.1714	1	5711	C16H20N4O2	(M+H)+	1.83
304.1100	304.1739	1	381	C16H20N4O2	(M+H)+	210.05

--- End Of Report ---

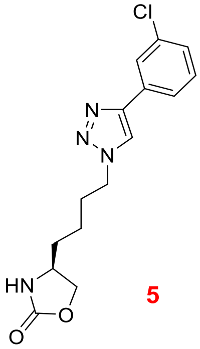

5

Chemical Formula: $C_{15}H_{17}ClN_4O_2$
Exact Mass: 320.10

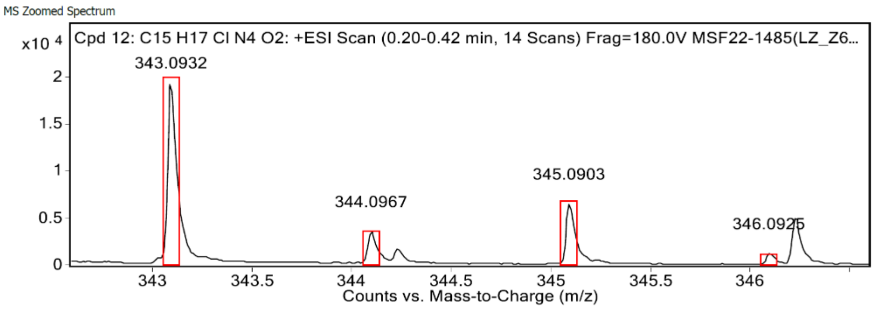

MS Spectrum Peak List

Obs. m/z	Calc. m/z	Charge	Abundance	Formula	Ion Species	Tgt Mass Error (ppm)
296.2074			2117257			
343.0932	343.0932	1	19791	C15H17ClN4O2	(M+Na)+	0.01
344.0967	344.0961	1	3716	C15H17ClN4O2	(M+Na)+	-1.85
345.0903	345.0908	1	6641	C15H17ClN4O2	(M+Na)+	1.29
346.0925	346.0934	1	1370	C15H17ClN4O2	(M+Na)+	2.61

--- End Of Report ---

Oligomer Z6_2: *4e5f

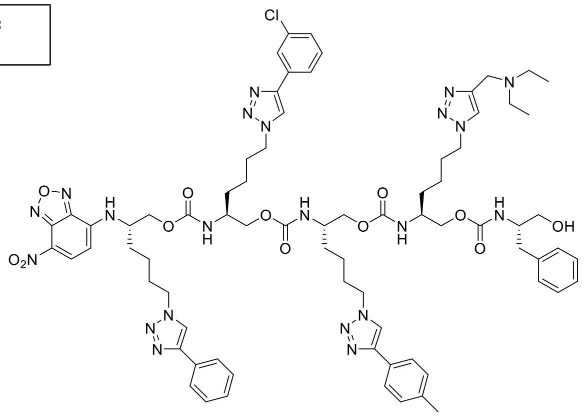

Exact Mass: 1515.71

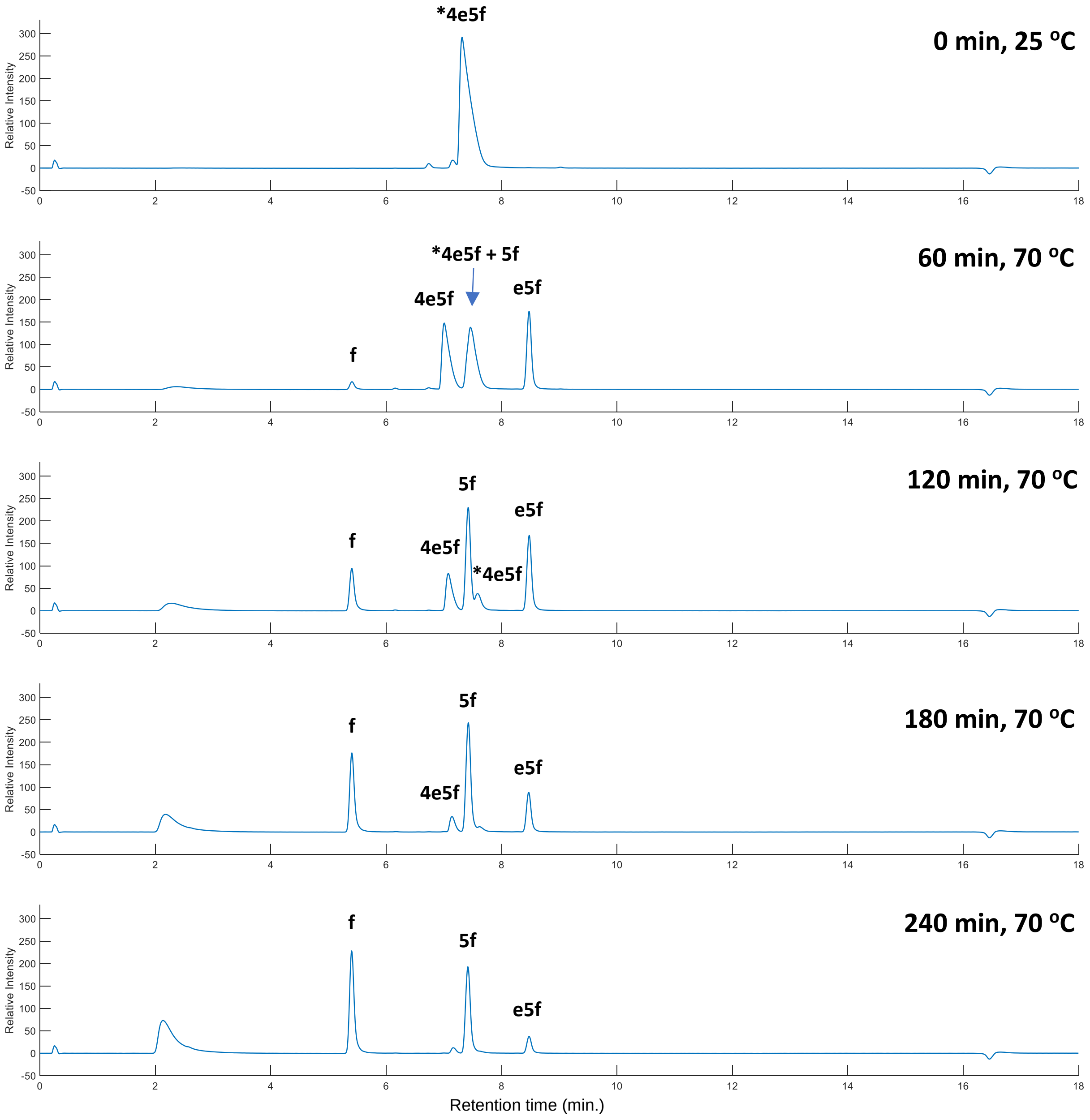

References

1. Dahlhauser, S. D.; Escamilla, P. R.; Vandewalle, A. N.; York, J. T.; Rapagnani, R. M.; Shei, J. S.; Glass, S. A.; Coronado, J. N.; Moor, S. R.; Saunders, D. P.; Anslyn, E. V. *J. Am. Chem. Soc.* **2020**, *142* (6), 2744–2749.

III(c). Sequencing the oligomers with overlapping truncated oligomers of similar polarities in LC

Oligomer 4: #7fd2

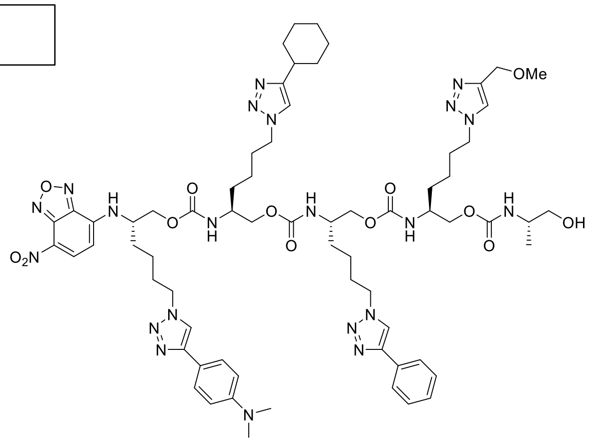

Exact Mass: 1399.73

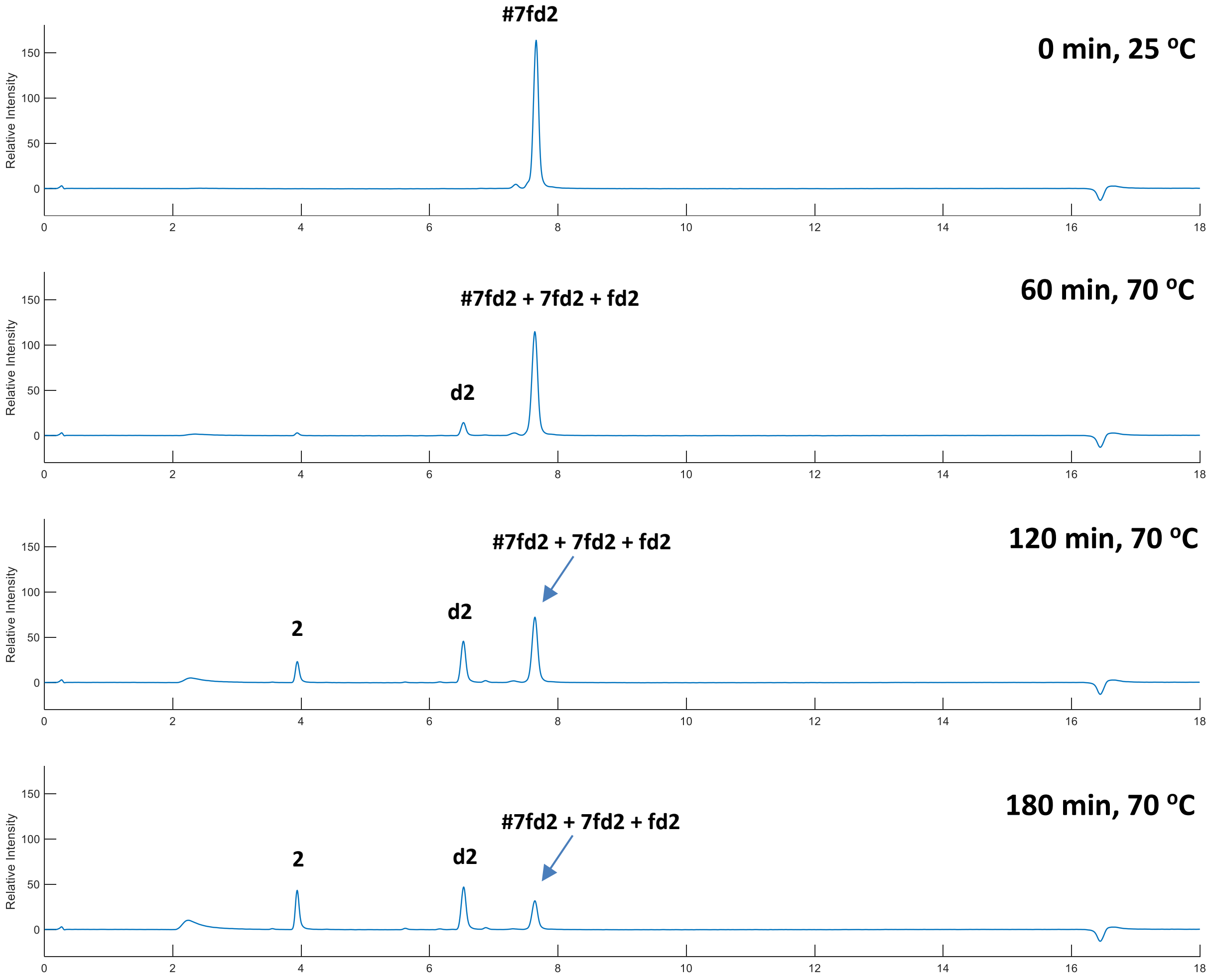

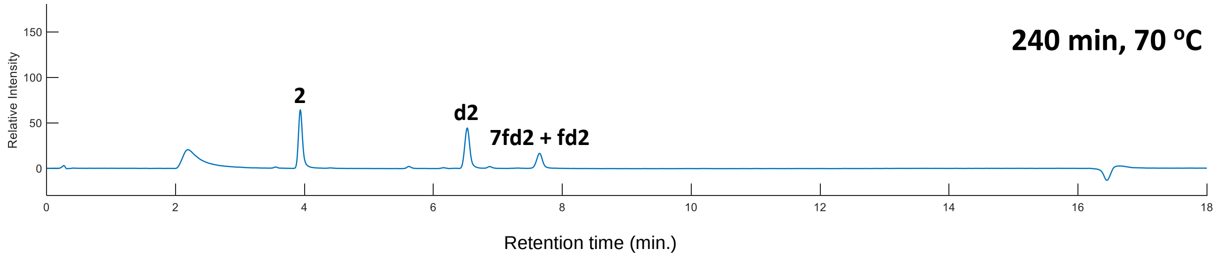

The sequencing of Oligomer 4 (#7fd2) is a scenario where multiple peaks overlap. In this case, it can be difficult to identify what peaks are overlapping, and what is the order in which these peaks show up in LC-MS. The MS data can help in deconvolute that. The process is shown below:

At 0 min, there is only one peak in the LC trace and the corresponding MS for that peak is shown below:

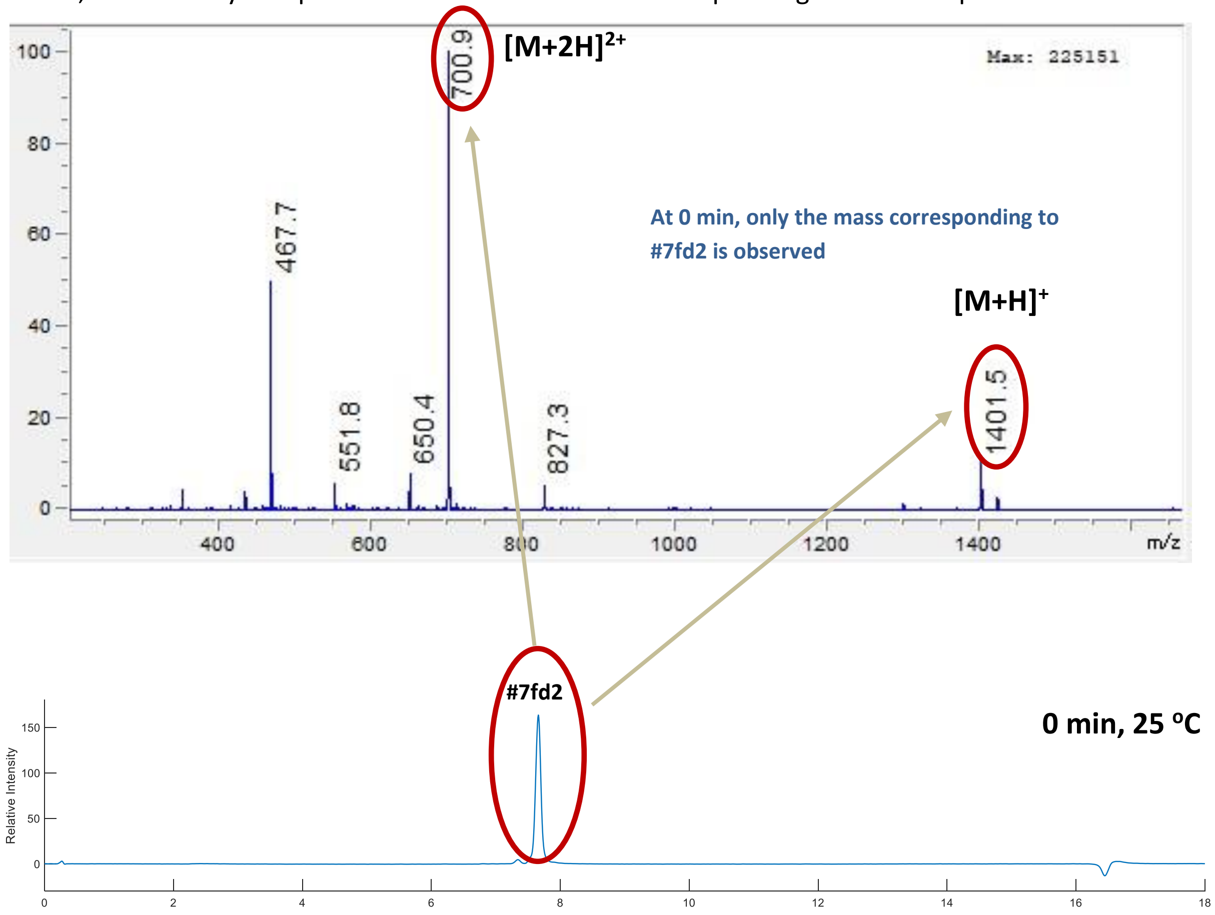

At 60 min, there are two peaks in the LC trace, and the corresponding MS for these peaks are shown below:

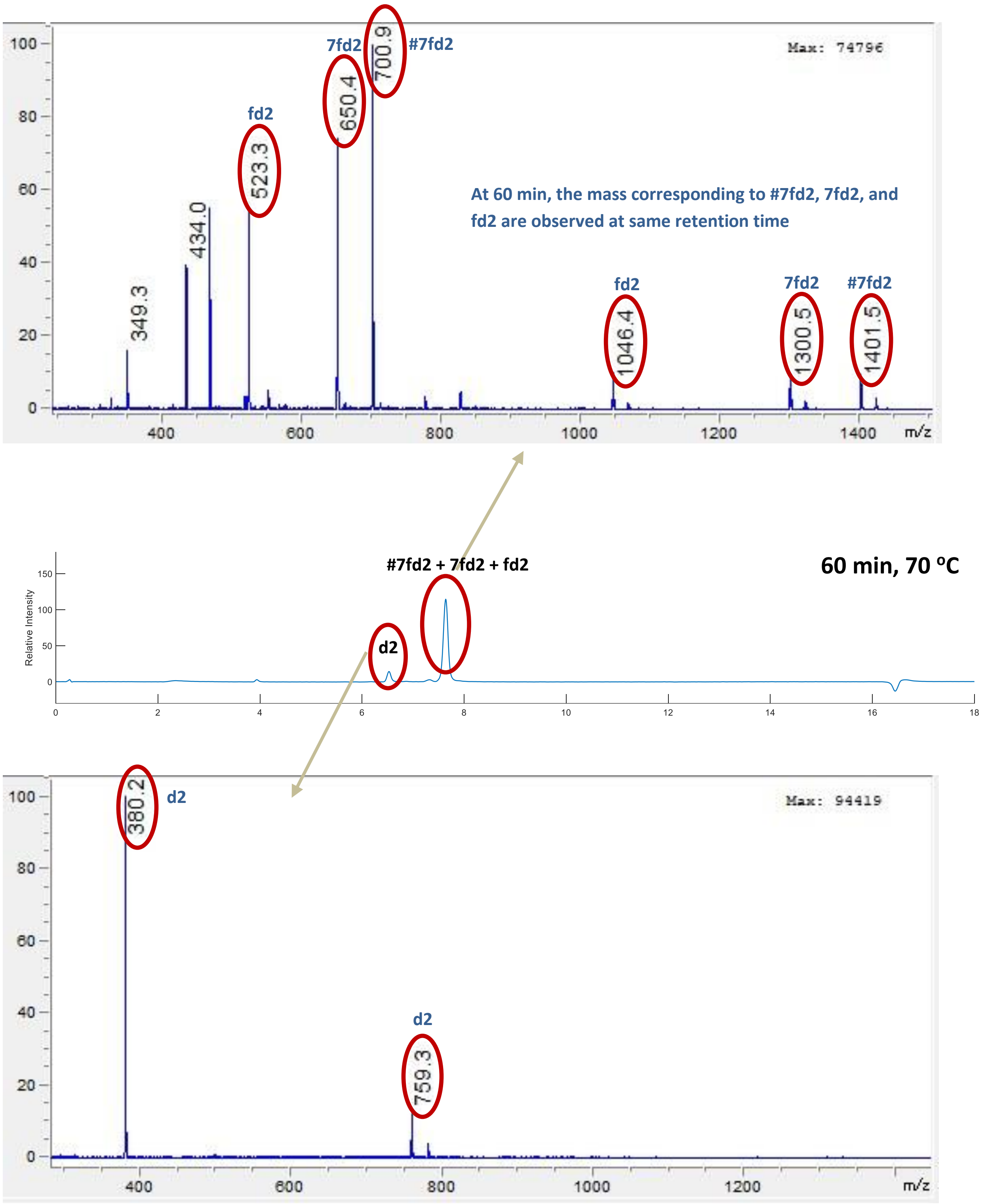

At 120 min, there are three peaks in the LC trace, and the corresponding MS for these peaks are shown below:

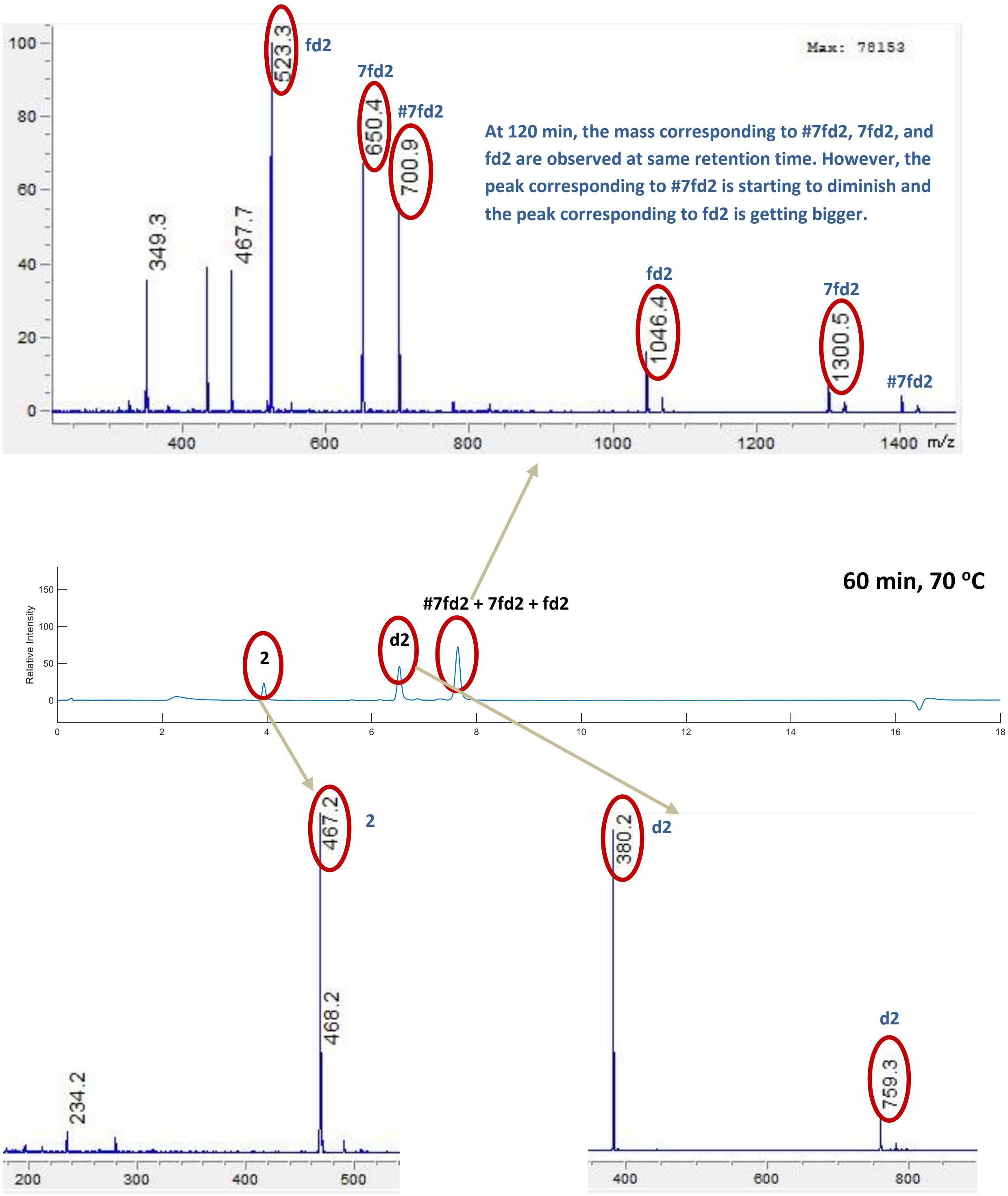

As we keep observing the LC trace and the corresponding MS peaks for the 180 and 240 minutes, we can confidently say what peaks are overlapping and what is the order of their appearance and disappearance. From this observation, the accurate sequence of the given oligomer can be determined.

III(d). The oligomers and corresponding Chinese characters.

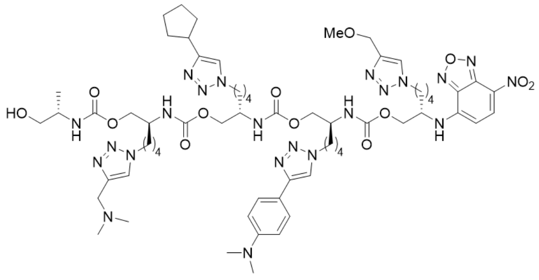

6027

性

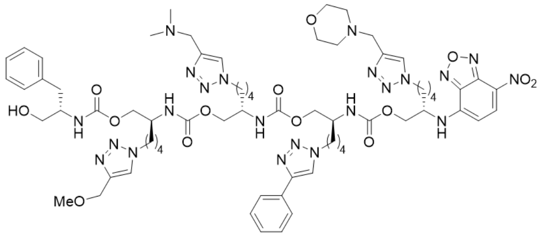

76f8

相

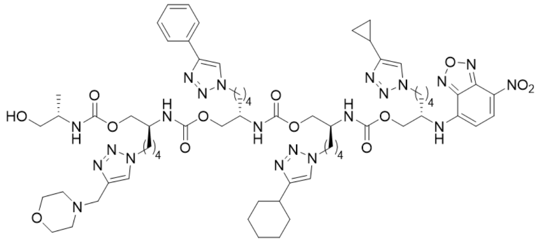

8fd1

近

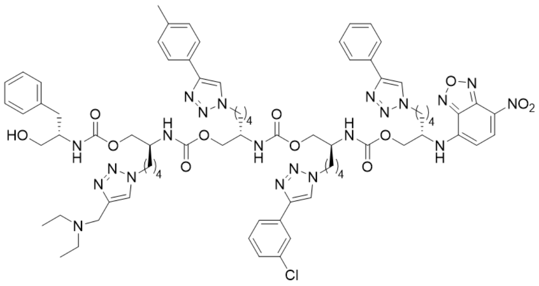

4e5f

也

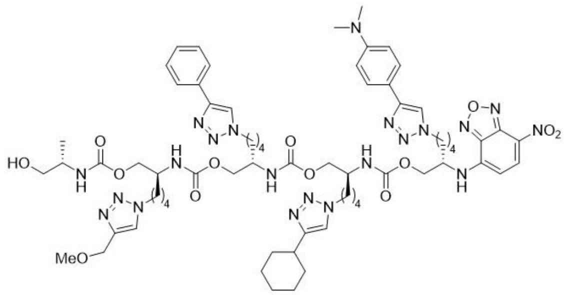

7fd2

習

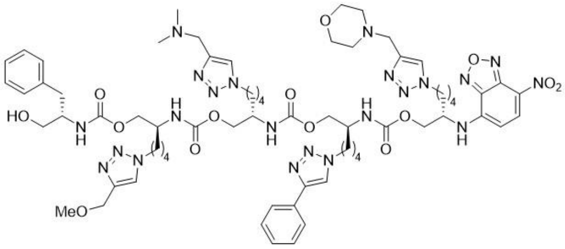

76f8

相

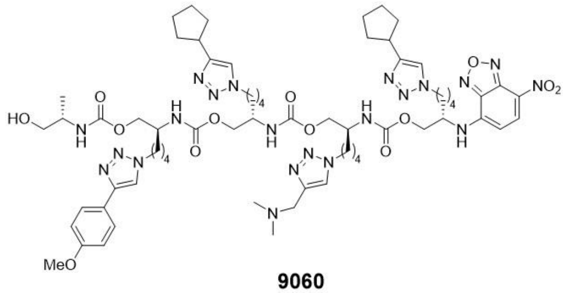

9060

遠

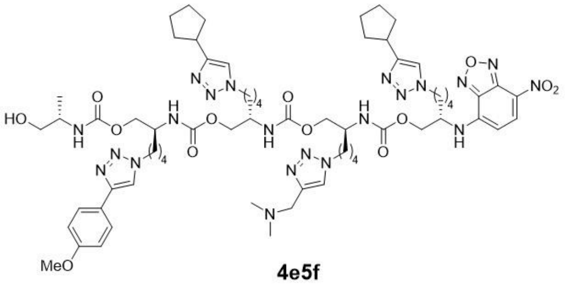

4e5f

也

(1) Unicode encoding

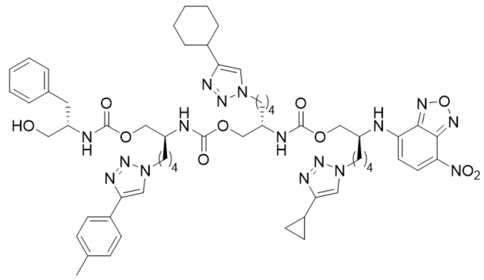

UMC
性

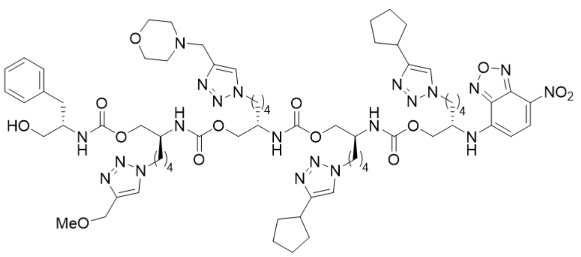

FLVV
相

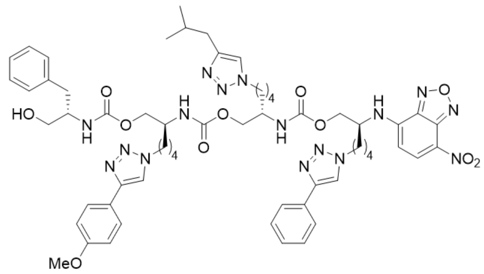

PDW
近

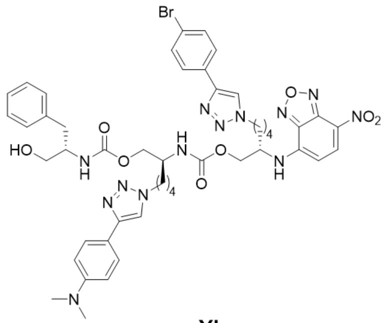

YI
也

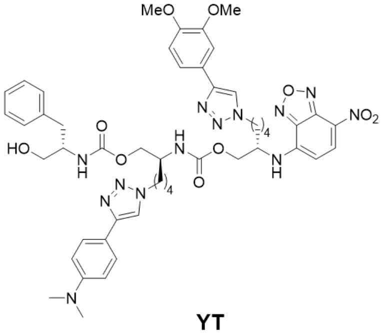

YT
习

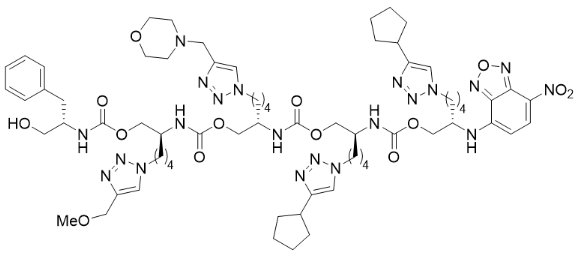

FLVV
相

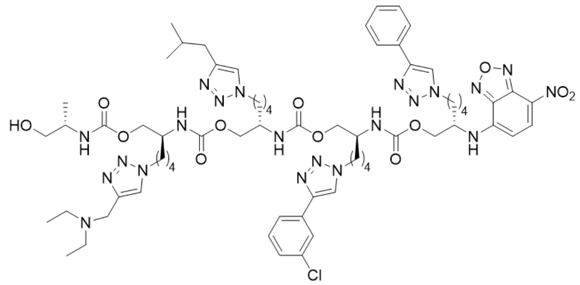

BDRW
远

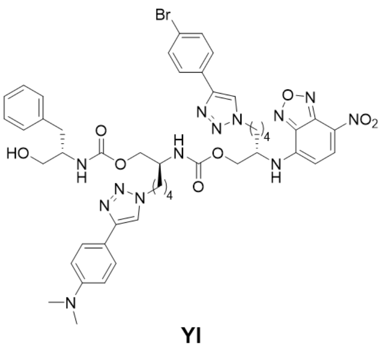

YI
也

(2) Zhengma encoding

III(e). The zoomed in mass spectra of Oligomer Z5 (*flvv) and Z6 (*4e5f).

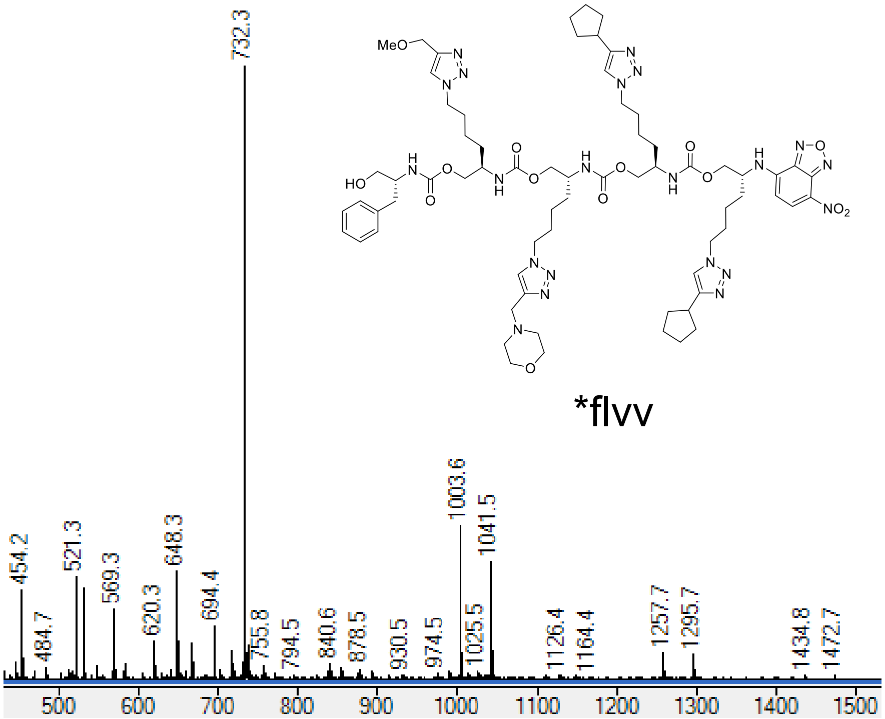

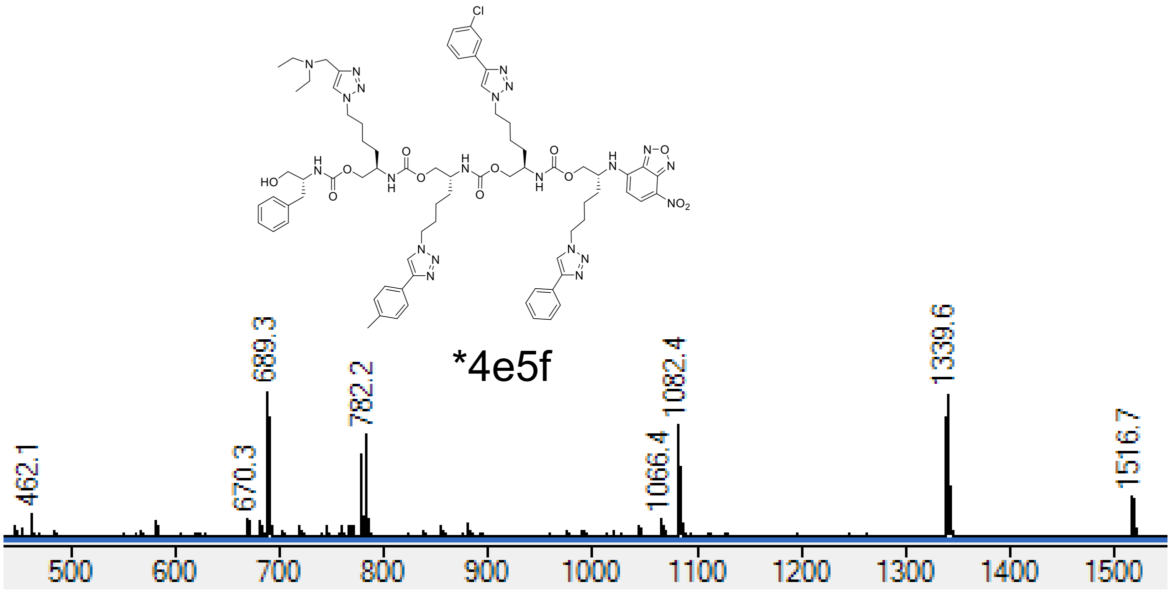

IV. User manual for Python scripts

To use this program, download the zip file, which can be located by clicking on “Code” on the GitHub website.

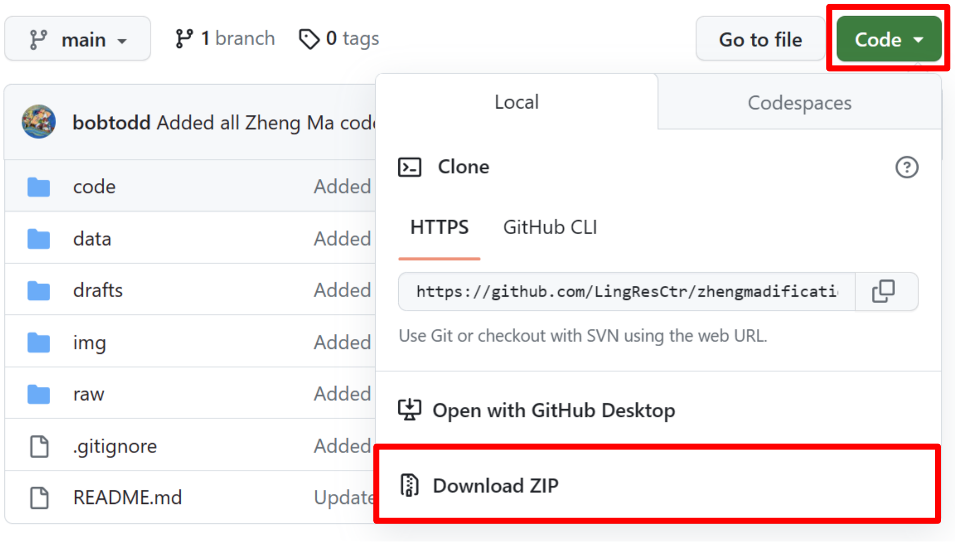

- To convert from Chinese characters to Zhengma codes

1. Go to the “code” folder.

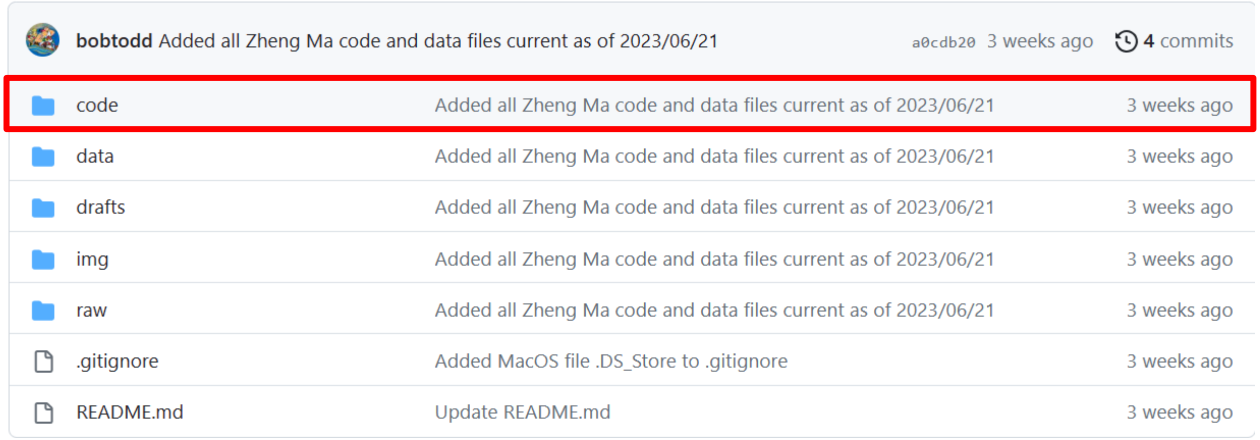

2. Open the “4_converter.ipynb” file.

Name	Last commit message	Last commit date
..		
1_background.ipynb	Added all Zheng Ma code and data files current as of 2023/06/21	3 weeks ago
2_data.ipynb	Added all Zheng Ma code and data files current as of 2023/06/21	3 weeks ago
3_tests.ipynb	Added all Zheng Ma code and data files current as of 2023/06/21	3 weeks ago
4_converter.ipynb	Added all Zheng Ma code and data files current as of 2023/06/21	3 weeks ago
zm_helpers.py	Added all Zheng Ma code and data files current as of 2023/06/21	3 weeks ago

3. Load and run the codes 1-4 shown below.

```
In [1]:
# If running in Google Colab
#from google.colab import drive
#drive.mount('/content/gdrive')
#
#path_prefix = "/content/gdrive/My Drive/Colab Notebooks/zhengma/raw/"
#data_prefix = '/content/gdrive/My Drive/Colab Notebooks/zhengma/data/'
```

```
In [2]:
# If running on local system
path_prefix = "../raw/"
data_prefix = '../data/'
```

```
In [3]:
import pickle

# Load pickle
with open(data_prefix + 'df_zm_merged.pkl', 'rb') as pickle_file:
    df_zm_merged = pickle.load(pickle_file)
```

```
In [4]:
def characters_to_codes_simplistic(cjk_string, zm_dataframe, db_column='RIME Characters', zm_column='ZM Codes'):
    # Input:
    #   string of CJK characters
    #   database of Zheng Ma codes as a pandas DataFrame
    #   name of column to check for characters
    #   name of column containing Zheng Ma codes
    # Output:
    #   list (dictionary?) of Zheng Ma codes
    #     - In case of multiple code correspondences, choose the longest

    characters = cjk_string.strip().replace(' ', '')

    codes = []

    for character in characters:
        # Find any rows in the desired column that have the desired character
        # Take the ZM codes in those rows as a list
        possible_codes = zm_dataframe[zm_dataframe[db_column] == character][zm_column].tolist()

        # Choose the **longest code** in that list of ZM codes
        max_code = max(possible_codes, key=len) if possible_codes else None
        # There could be several, so order alphabetically and pick the first
        desired_codes = [c for c in possible_codes if len(c) == len(max_code)] if max_code else None
        desired_code = sorted(desired_codes)[0] if desired_codes else 'N/A: no match'
        codes.append([character, desired_code])

    return codes
```

4. In code line 5, enter the Chinese characters to be encoded, encased in quotation marks as shown in the red box. The example below reads “Zhengma Method” in Chinese.

```
In [5]: new_test_string1 = '郑码输入法'
```

5. Run code 6 to obtain the Zhengma codes that correlate to the Chinese characters.

```
In [6]: new_test_codes_output1 = characters_to_codes_simplistic(new_test_string1, df_zm_merged)
        print(new_test_codes_output1)

        [['郑', 'uagy'], ['码', 'gxvv'], ['输', 'heqk'], ['入', 'oda'], ['法', 'vbzs']]
```

# - To convert from Zhengma codes to Chinese characters

1. Go to the “code” folder.

**bobtodd** Added all Zheng Ma code and data files current as of 2023/06/21	a0cdb20 3 weeks ago	4 commits
code	Added all Zheng Ma code and data files current as of 2023/06/21	3 weeks ago
data	Added all Zheng Ma code and data files current as of 2023/06/21	3 weeks ago
drafts	Added all Zheng Ma code and data files current as of 2023/06/21	3 weeks ago
img	Added all Zheng Ma code and data files current as of 2023/06/21	3 weeks ago
raw	Added all Zheng Ma code and data files current as of 2023/06/21	3 weeks ago
.gitignore	Added MacOS file .DS_Store to .gitignore	3 weeks ago
README.md	Update README.md	3 weeks ago

2. Open the “4_converter.ipynb” file.

Name	Last commit message	Last commit date
..		
1_background.ipynb	Added all Zheng Ma code and data files current as of 2023/06/21	3 weeks ago
2_data.ipynb	Added all Zheng Ma code and data files current as of 2023/06/21	3 weeks ago
3_tests.ipynb	Added all Zheng Ma code and data files current as of 2023/06/21	3 weeks ago
4_converter.ipynb	Added all Zheng Ma code and data files current as of 2023/06/21	3 weeks ago
zm_helpers.py	Added all Zheng Ma code and data files current as of 2023/06/21	3 weeks ago

3. Load and run the codes 1-3 and 8 shown below.

```
In [1]: # If running in Google Colab
        #from google.colab import drive
        #drive.mount('/content/gdrive')
        #
        #path_prefix = "/content/gdrive/My Drive/Colab Notebooks/zhengma/raw/"
        #data_prefix = '/content/gdrive/My Drive/Colab Notebooks/zhengma/data/'
```

```
In [2]: # If running on local system
        path_prefix = "../raw/"
        data_prefix = '../data/'
```

```
In [3]: import pickle

        # Load pickle
        with open(data_prefix + 'df_zm_merged.pkl', 'rb') as pickle_file:
            df_zm_merged = pickle.load(pickle_file)
```

```
In [8]: def codes_to_characters_simplistic(code_list, zm_dataframe, db_column='RIME Characters', zm_column='ZM Codes'):
            # Input:
            #   list of ZM codes
            #   database of Zheng Ma codes as a pandas DataFrame
            #   name of column to check for characters
            #   name of column containing Zheng Ma codes
            # Output:
            #   string of CJK characters
            #     - In case of multiple character correspondences for a code, choose...

            cjk_string = ''

            for code in code_list:
                # Make sure the code is a valid ZM code:
                #   - fewer than 5 letters
                #   - no spaces
                if ' ' not in code:
                    if len(code) < 5:
                        # Get the characters for that code
                        possible_characters = zm_dataframe[zm_dataframe[zm_column] == code][db_column].tolist()
                        # Remove any empty strings
                        viable_characters = [x for x in possible_characters if (len(x) > 0)]
                        # Add the smallest string (hopefully 1 character)
                        # ... watch out: there might be more than one minimum...
                        # ... what does min() do?  return the first it finds in the list?
                        cjk_string += min(viable_characters, key=len)
                    else:
                        print('Code too long: {}'.format(code))
                else:
                    print('Code should not contain spaces: {}'.format(code))

            return cjk_string
```

4. In code line 10, enter the Zhengma codes to be decoded back to Chinese characters as shown in the red box below. Each Zhengma code should be encased in quotation marks as well as separated from the other Zhengma codes by commas. Then, all Zhengma codes should be encased in a pair of brackets. Running this code will return the Chinese characters correlating to the Zhengma codes.

```
In [10]: new_test_string_output1 = codes_to_characters_simplistic(['uagy','gxvv', 'heqk', 'oda', 'vbzs'], df_zm_merged)
         print(new_test_string_output1)

         郑码输入法
```
